# Organocatalytic Formal (3+2+1)‐Cycloaddition of Allenoates, Michael Acceptors, and Carbaldehydes

**DOI:** 10.1002/chem.71222

**Published:** 2026-06-04

**Authors:** David Naderer, Alexander Pötscher, Katharina Eder, Uwe Monkowius, Markus Himmelsbach, Mario Waser

**Affiliations:** ^1^ Institute of Organic Chemistry Johannes Kepler University Linz Linz Austria; ^2^ School of Education Chemistry Johannes Kepler University Linz Linz Austria; ^3^ Institute of Analytical Chemistry Johannes Kepler University Linz Linz Austria

**Keywords:** allenoates, cycloadditions, Lewis bases, organocatalysis, spirocyclic compounds

## Abstract

Employing secondary amines as covalently interacting Lewis basic organocatalysts allows for the activation of electron‐deficient allenes (allenoates) for unprecedented formal (3+2+1)‐cycloadditions with Michael acceptors and aromatic carbaldehydes. This strategy is proposed to proceed via nucleophilic addition of the catalyst to the allenoate's β‐carbon first, followed by γ‐addition to the Michael acceptor, a subsequent aldol‐type step, and the final ring closure and catalyst liberation. Hereby, direct access to various highly functionalized spirocyclic cyclohexanone‐derivatives (17 examples) in good to excellent yields (up to 98%) and a first proof‐of‐concept for an asymmetric variant (around 75:25 er) by using chiral secondary amine catalysts has been obtained as well.

## Graphical abstract

The use of secondary amine Lewis base organocatalysts allows for the unprecedented activation of allenoates for formal (3+2+1)‐cycloadditions with Michael acceptors and aromatic carbaldehydes.

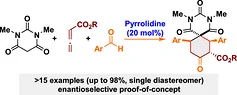

## 1 Introduction

**SCHEME 1:**
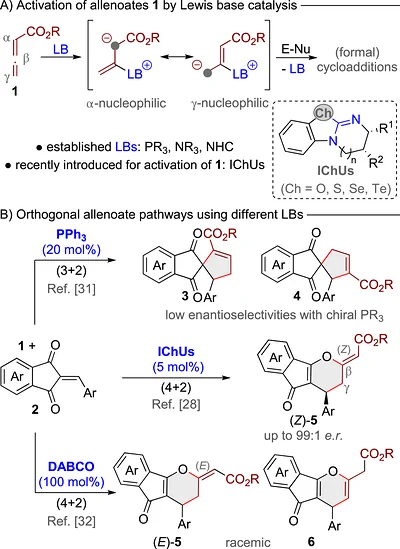
(A) General activation of allenoates **1** using Lewis bases (LBs) and (B) the orthogonal reactivity of allenoates depending on the used LB scaffold, exemplified for the reaction of **1** and **2**.

Electron‐deficient allenes, such as allenoates **1**, are readily accessible building blocks that can undergo a broad variety of unique transformations, either in the absence or presence of a catalyst [1, 2, 3, 4, 5, 6]. With respect to catalyzed processes, especially the use of (chiral) nucleophilic Lewis base (LB) organocatalysts [7, 8, 9] enables a multitude of complex (chiral) target generating approaches [1, 2, 3, 4, 5, 6, 7, 8, 9, 10, 11, 12, 13, 14, 15, 16, 17, 18, 19, 20, 21, 22]. Activation hereby proceeds via addition of the LB to the sp‐hybridized β‐carbon of the allenoate first, thus resulting in the formation of a highly reactive resonance‐stabilized betaine species (Scheme 1A). Depending on the nature of the LB catalyst, as well as the substitution of the allenoate, either the α‐ or the γ‐carbon may be the most nucleophilic position. These betaine species can then react with different acceptor molecules, mainly dipolar cycloaddition partners (E‐Nu), such as carbonyl derivatives or Michael‐type acceptors. This allows for a broad variety of different structural complexity generating (hetero)‐cycloaddition processes [1, 2, 3, 4, 5, 6, 10, 11, 12, 13, 14, 15, 16, 17, 18, 19, 20, 21, 22].] With respect to the used catalyst classes, the field is mainly dominated by (chiral) tertiary phosphines [9, 10, 11, 12, 13, 14] and, to a lesser extent, by tertiary amines [15, 16, 17, 18, 19] and N‐heterocyclic carbenes [20, 21, 22]. Remarkably, the nature of the used catalyst has a pronounced effect on the reactivity of the betaines, allowing for orthogonal reaction pathways when changing the catalyst motif. Over the last few years, our group has successfully established isochalcogenoureas (IChUs) [23, 24, 25, 26] as versatile catalysts for allenoate‐based asymmetric cycloadditions [27, 28, 29, 30]. Remarkably, throughout all our studies carried out so far, the use of these catalysts generally resulted in (4+2)‐heterocycloadditions giving dihydropyranes with a (*Z*)‐configurated exocyclic double bond, independent of the nature of the used allene or Michael acceptor. Accordingly, this strategy represents a powerful complementary concept compared to established tert. phosphine‐ or tert. amine‐catalyzed processes, as exemplified for the orthogonal results of the reaction between allenoates **1** and acceptors **2** in the presence of these different catalyst classes (Scheme 1B) [28, 31, 32].

**SCHEME 2:**
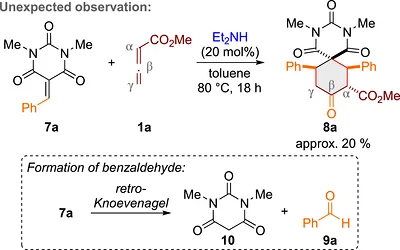
First observation of the formation of spiro‐compound **8a** during the reaction of Michael acceptor **7a** and allenoate **1a** in the presence of diethylamine.

Based on the unique potential of allenoates to enter different reaction pathways depending on the used LB catalyst, we are now becoming interested in testing alternative classes of Lewis bases for allenoate cycloadditions. While tert. amines have been commonly employed [15, 16, 17, 18, 19], sec. amines (R_2_NH) have, to the best of our knowledge, not been properly established for allenoate activation. Thus, we were wondering if it may be possible to enable new allenoate transformations by using sec. amines as catalysts. In the first test reactions, we screened some simple amines for the reaction of the barbiturate‐based acceptor **7a** with allenoate **1a**. Hereby, we made an unexpected observation when applying diethylamine. As outlined in Scheme 2, we could identify the spirocyclic compound **8a** as a distinct reaction product, hereby (besides vast amounts of unidentified side‐products; structural assignment as well as determination of the relative configuration were unambiguously possible by NMR analysis and single‐crystal x‐ray diffraction [33]). Our rationale for the formation of **8a** is that **7a** partially undergoes a retro‐Knoevenagel reaction, thereby forming benzaldehyde (**9a**) first. This aldehyde then serves as a C1 building block in a formal sec. amine‐catalyzed three‐component (3+2+1)‐cycloaddition sequence with the allenoate **1a** (C3 synthon, activated by the amine) and compound **7a** (C2 partner) (vide infra).

Based on this surprising outcome and the unprecedented reaction pathway observed in these first experiments, we therefore set out to investigate this transformation systematically. As outlined in this contribution, the use of simple secondary amines actually allows for novel allenoate‐based cycloaddition pathways, thus opening new chemical space by just changing the catalyst motif (to compare: compounds **7** also follow the (*Z*)‐selective (4+2)‐cycloaddition pathway depicted in Scheme 1B under IChU catalysis instead [29]).

## 2 Results and Discussion

### 2.1 Optimization of Reaction Conditions

We started our investigations by testing a few classical sec. amines for the reaction of allenoate **1a**, Michael acceptor **7a,** and benzaldehyde **9a** first (Table 1, entries 1–4). Interestingly, the basicity of the amine seems to play an important role here. While the stronger bases Et_2_NH, pyrrolidine, and piperidine (with p*K*_a_ (H_2_O) of the conjugated acids around 11 [34]) led to product **8a** formation, the roughly three orders of magnitude less basic morpholine [34] did not allow for any noteworthy product formation (also, the less basic tert. amine DABCO did not give any product; entry 5). This observation may be rationalized by mechanistically crucial protonation/deprotonation events and the generation and stabilization of anionic key intermediates herein (vide infra, Scheme 4). The better performance of pyrrolidine (entry 3) as compared to Et_2_NH (entry 1) may be explained by its higher nucleophilicity [35, 36]. However, high nucleophilicity of the amine seems to be only one important aspect, as piperidine, which is only slightly less nucleophilic in aprotic solvents as compared to pyrrolidine [36], was found to be less well‐suited (entry 2), resulting in the formation of significant amounts of unidentified side‐products. Further optimization of reaction conditions using pyrrolidine then showed that the reaction is benefiting from the use of more polar solvents but is, in general rather tolerant to different ones (entries 6–10), as long as the reaction temperature is not reduced below 60°C (entries 11 and 12).

**TABLE 1:** Optimization of reaction conditions for the synthesis of spiro‐compound **8a**.

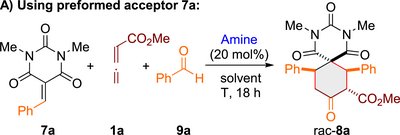				

Entry^a^	Amine	Solvent	T (°C)	Yield (%)^b^
1	Et_2_NH	toluene	80	41
2	piperidine	toluene	80	19
3	pyrrolidine	toluene	80	61
4	morpholine	toluene	80	< 5%
5	DABCO	toluene	80	0
6	pyrrolidine	tBuOH	80	81
7	pyrrolidine	CPME	80	84
8	pyrrolidine	EtOAc	80	67
9	pyrrolidine	CH_3_CN	80	41
10	Pyrrolidine	DCE	80	82
11	Pyrrolidine	DCE	60	79
12	Pyrrolidine	DCE	40	14
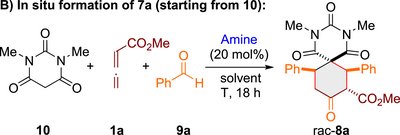				

Entry^c^	Amine	Solvent	T (°C)	Yield (%)^b^
13	Pyrrolidine	DCE	80	98^d^
14	Pyrrolidine	tBuOH	80	91
15	Pyrrolidine	CPME	80	60
16	Pyrrolidine	DCE	60	77
17	Pyrrolidine	DCE	40	78
18	Pyrrolidine (10%)	DCE	80	37

^a^ All reactions given in entries 1–12 were carried out using 0.05 mmol **7a**, 0.1 mmol allenoate **1a**, and 0.06 mmol **9a** under the indicated conditions (DCE = 1,2‐dichloroethane; CPME = cyclopentylmethylether).

^b^ NMR yields determined using mesitylene as an internal standard (apart from entry 13, which gives the isolated yield).

^c^ All reactions given in entries 13–17 were carried out using 0.05 mmol **10**, 0.1 mmol allenoate **1a**, and 0.11 mmol **9a** under the indicated conditions.

^d^ Isolated yield on 1 mmol scale.

Having shown that this amine‐catalyzed formal (3+2+1)‐cycloaddition process is indeed a robust process when using **1a**, **7a**, and **9a** (entries 1–12), we were also wondering if we could maybe start directly from barbituric acid derivative **10** instead of its arylidene‐derivative **7a** (entries 13–17). We rationalized that **10** should undergo a rapid Knoevenagel condensation with benzaldehyde **9a** under the reaction conditions, providing **7a** in situ, thus avoiding the need of synthesizing it upfront. Gratifyingly, when reacting **10** with allenoate **1a** and 2 equiv. of benzaldehyde **9a** in the presence of pyrrolidine, this strategy worked very well, giving product **8a** in almost quantitative isolated yield when using DCE as the solvent (entry 13). Other solvents were again well‐tolerated (entries 13–15), and interestingly, this protocol also delivered the product **8a** in still reasonable yields at lower T (entries 16, 17). Unfortunately, lowering the catalyst loading to 10 mol% led to a strongly reduced yield (entry 18).

It should be stated that in all cases where yields were low, significant amounts of unreacted arylidene‐barbiturate **7a** could be detected, thus underscoring that formation of this species is a fast process as compared to the actual cycloaddition sequence (the same observation was made when investigating the application scope of this process).

### 2.2 Mechanistic Considerations

**SCHEME 3:**
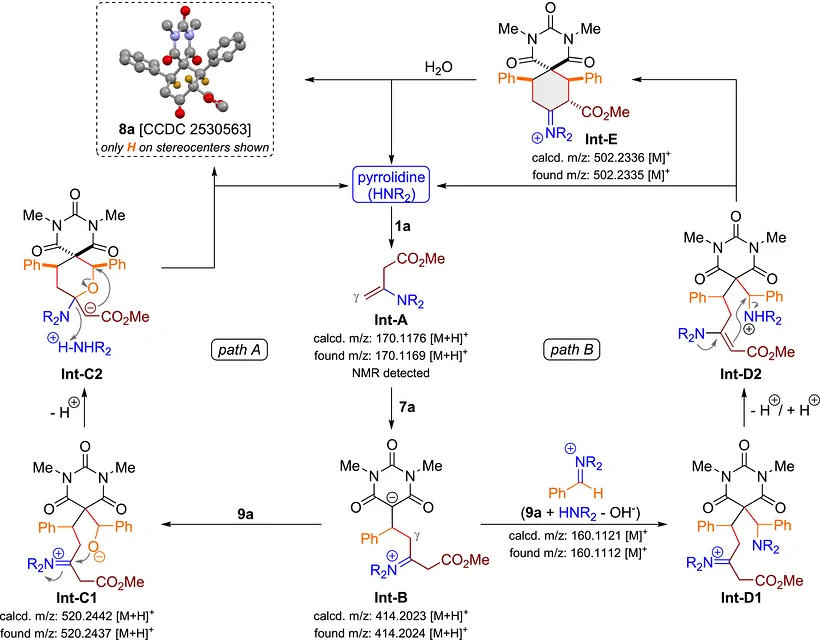
Proposed mechanistic scenario.

We propose that the formation of product **8a** can be explained by either of the two mechanistic pathways depicted in Scheme 3 (*we gratefully acknowledge very convincing and detailed suggestions by one of the initial reviewers*!). First, the pyrrolidine catalyst undergoes β‐addition to the allenoate **1a**, which, upon protonation (e.g., by the barbituric acid **10**), gives **Int‐A** (NMR and HRMS detected). This enamine‐activated allenoate adds to the (in situ formed) Michael acceptor **7a** via its γ‐carbon, resulting in the formation of **Int‐B** (HRMS detected). **Int‐B** then either (path A) reacts with benzaldehyde (**9a**) in an aldol manner first (**Int‐C1**), followed by an intramolecular hemiaminal formation (**Int‐C2**) and subsequent α‐deprotonation, rearrangement, and catalyst release (species with *m/z* values corresponding to **Int‐C** were detected by HRMS). Alternatively (path B), **Int‐B** may also add to the in situ‐generated imine of aldehyde **9a** and pyrrolidine (HRMS detected) in a Mannich reaction. This would lead to **Int‐D1** first, which could then react to **Int‐E** via **Int‐D2**. While **Int‐D** could not be detected by HRMS (hydrolysis would actually result in the formation of species with the same m/z values as observed for **Int‐C**), **Int‐E** was again observed during our HRMS analysis of the reaction mixture. Overall, based on these observations, we speculate that path B may thus be the more likely one. The high preference for one single diastereoisomer with the two phenyl groups adopting a cis‐configuration and the ester being trans to the phenyls can be best rationalized by the fact that this relative configuration allows for a stable “all‐equatorial” orientation of the two phenyl groups and the ester functionality, which was also confirmed by single crystal x‐ray diffraction of **8a** [33].

### 2.3 Application Scope

**SCHEME 4:**
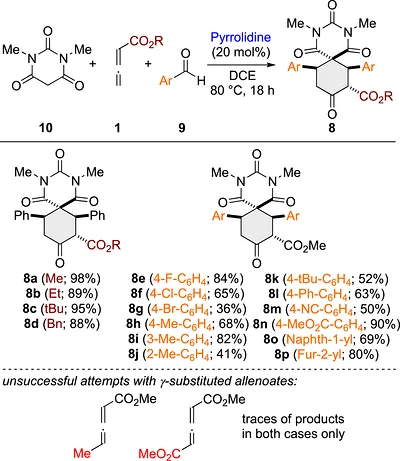
Application scope for the syntheses of spiro‐cyclohexane products **8**.

Having identified high‐yielding conditions for the direct synthesis of **8a** starting from barbituric acid **10**, we next examined the generality of this strategy. As outlined in Scheme 4, a variety of different allenoates **1** and aromatic aldehydes **9** were, in general well tolerated, delivering products **8** in moderate to excellent isolated yields. While different ester groups had literally no influence on the performance (compare products **8a**–**d**), the nature of the aldehyde had some impact, and in the lower yielding cases (like **8g**, **8j**, or **8m**), we detected significant amounts of unreacted arylidene‐barbiturates **7** (which were formed in situ from **10** and **9**). This observation suggests that the cycloaddition sequence is the limiting part of the overall process, not the formation of compounds **7**. It should also be stated that no noteworthy quantities of any other diastereomers were detected, even in the lower‐yielding experiments.

Attempting the use of γ‐substituted allenoates unfortunately failed, as we observed only traces of products in these cases (besides large amounts of unreacted arylidene‐barbiturates **7**).

**SCHEME 5:**
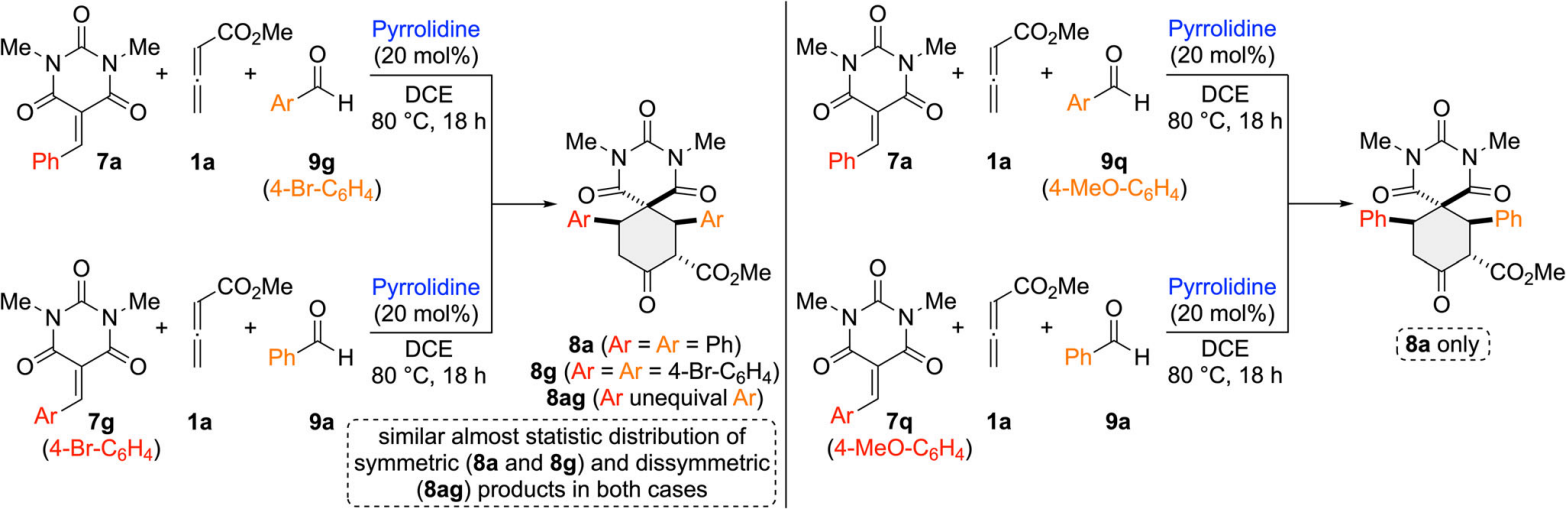
Attempts to access products **8** with two different aryl substituents.

To extend this methodology towards products **8** containing two different aryl‐substituents, we next investigated the reaction of allenoate **1a** with the preformed acceptor **7a** and the Br‐substituted benzaldehyde **9g** under the optimized conditions (Scheme 5, upper left entry). Interestingly, we hereby observed formation of the “homo”‐aromatic products **8a** and **8g** besides the actually targeted “hetero”‐aryl products **8ag** (both possible regioisomers, namely the one with the phenyl ring in the “red” position and the Br‐phenyl group in the “orange” position, as well as the “inverted” one were formed). Alternatively, using starting material **7** **g** and benzaldehyde **9a** also yielded the same mixture of products with a similar more or less statistic distribution of the four possible products (in both experiments one of the two “hetero”‐products was formed more selectively than the other but due to the fact that the “homo”‐products made up for at least 50% of the overall amount we did not investigate this in more detail anymore). These results clearly demonstrate the lability of the used arylidene‐barbiturates **7** and their tendency to readily undergo the retro‐Knoevenagel reaction under these reaction conditions, thus leading to a random installation of the electronically similar aldehydes **9a** and **9g**. On the other hand, attempts to incorporate a more electron‐rich aryl group, such as p‐MeO‐C_6_H_4,_ besides the phenyl substituent failed (Scheme 5, left side). Both attempts, either employing aldehyde **9q** besides **7a** or testing acceptor **7q** with aldehyde **9a**, always resulted in exclusive formation of the diphenyl product **8a**, underscoring again the reversibility of the Knoevenagel step and the significantly different reactivities of these two carbaldehydes.

**SCHEME 6:**
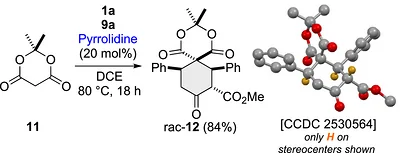
Analogous utilization of Meldrum's acid (11) for this cycloaddition process.

In an attempt to expand the applicability of this methodology, we also tested the use of Meldrum's acid (**11**) instead of barbituric acid **10**. Gratifyingly, this alternative starting material could be used in strict analogy under identical reaction conditions, delivering the spirocyclic cycloaddition product **12** in high yield as a single diastereoisomer (Scheme 6) [33]. Unfortunately, however, using simple malonates (or analogous bissulfones) instead of the cyclic active methylene‐containing compounds **11** (or **10**) did not allow for any product formation.

### 2.4 Enantioselective Proof‐of‐Concept

**SCHEME 7:**
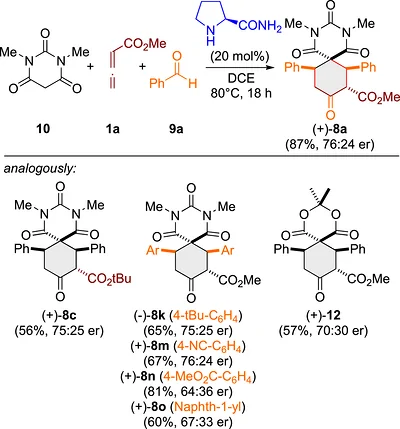
Enantioselective proof‐of‐concept.

Finally, we also wondered if we could render these transformations enantioselective by using chiral pyrrolidine derivatives as catalysts. After testing a variety of proline‐based secondary amines, we could identify proline amide as an easily available catalyst allowing for the synthesis of **8a** in good yield and a moderate enantioselectivity of 76:24 er (Scheme 7). Unfortunately, further optimizations were found to be not fruitful (further details can be found in the online Supporting Information) and, as outlined in the lower part of Scheme 7, also other starting material combinations did not allow for any improved enantioselectivities. Nevertheless, these results provide the first proof‐of‐principle that an enantioselective variant is feasible.

## 3 Conclusion

We herein report an operationally simple method for formal (3+2+1)‐cycloadditions of allenoates, Michael acceptors, and benzaldehydes delivering highly functionalized spirocyclic cyclohexanone derivatives. The key to success is the use of secondary amines, especially pyrrolidine, as catalysts for the covalent activation of allenoates. Interestingly, these organic (Lewis) bases have so far not been reported for allenoate activation, but, as demonstrated herein, actually allow for unprecedented formal cycloaddition reactions. Using chiral pyrrolidine derivatives also allowed for some moderate levels of enantioselectivities, although further improvements will be necessary here. Mechanistically, the sec. amine catalyst undergoes β‐addition to the allenoate first, thus providing a highly reactive enamine intermediate. This first step is in analogy to established allenoate‐activating Lewis bases such as tert. phosphines, tert. amines, NHCs, or IChUs. However, the herein reported use of sec. amines then allows for an orthogonal reaction pathway, thus expanding the already extensive and diverse range of applications for allenoates.

## Conflicts of Interest

The authors declare no conflicts of interest.

## Supporting information




**Supporting File**: The authors have cited additional references within the Supporting Information [37, 38, 39, 40].

## Data Availability

The data that support the findings of this study are available in the Supporting Information of this article.
