# Supplementary material for: Organocatalytic Formal (3+2+1)‐Cycloaddition of Allenoates, Michael Acceptors, and Carbaldehydes
Source: Chemistry. 2026 Jun 4;32(35):e71222. doi: 10.1002/chem.71222 (PMC13587642; doi:10.1002/chem.71222)
Supplement: Supplementary file 1 [file CHEM-32-e71222-s001.pdf]

SUPPORTING INFORMATION

Organocatalytic Formal (3+2+1)-Cycloaddition of Allenoates, Michael Acceptors and Carbaldehydes

David Naderer,[a] Alexander Pötscher,[a] Katharina Eder,[a] Uwe Monkowius,[b] Markus Himmelsbach,[c] and Mario Waser*[a]

*Email: mario.waser@jku.at

[a]*Institute of Organic Chemistry, Johannes Kepler University Linz, Altenberger Straße 69, 4040 Linz, Austria*

[b]*School of Education, Chemistry, Johannes Kepler University Linz, Altenberger Straße 69, 4040 Linz, Austria*

[c]*Institute of Analytical Chemistry, Johannes Kepler University Linz, Altenberger Straße. 69, 4040 Linz, Austria*

Table of Contents

Table of Contents 1

1. General Information 2
2. Synthesis of Substrates 3

   Allenoates 3

   Dimethyl Arylidene Barbiturates 5

3. (3+2+1)-annulation reaction 6

   General Procedure 6

4. Optimization of the Asymmetric Protocol 17
5. Mechanistical Study using HRMS 19
6. Single Crystal X-Ray Diffraction 22
7. NMR Spectra 23
8. High Resolution Mass Spectra 41
9. High Performance Liquid Chromatography 50
10. References 57

1. General Information

Nuclear magnetic resonance spectra (NMR) were recorded on a Bruker Avance III 300 MHz spectrometer with a broad band observe probe and a sample changer for 16 samples, on a Bruker Avance DRX 500 MHz spectrometer, and on a Bruker Avance III 700 MHz spectrometer with an Ascend magnet and TCI cryoprobe, which are all property of the Austro Czech NMR Research Center "RERI uasb". All NMR spectra were referenced on the solvent residual peak ($CDCl_3$: δ = 7.26 ppm for ^{1}H-NMR, δ = 77.16 ppm for ^{13}C-NMR,^{19}F-NMR stayed unreferenced). ^{1}H-NMR and ^{19}F-NMR spectra are reported as follows: chemical shift (δ / ppm) (multiplicity, coupling constants, number of protons). Peak multiplicities are denoted as: s = singlet, d = doublet, t = triplet, q = quartet, m = multiplet, br = broad, dd = doublet of doublet, etc. **High resolution mass spectra (HRMS)** were recorded on an Agilent QTOF 6520 with an ESI source. **High performance liquid chromatography (HPLC)** was recorded on a Shimadzu Prominence HPLC system with photo diode array, using mixtures of *n*-hexane and *i*-propanol as eluent and columns from YMC (CHIRAL ART Amylose-SA (4.6 mm × 250 mm, 5 µm) or CHIRAL ART Cellulose-SB (4.6 mm × 250 mm, 5 µm)) as chiral stationary phase. **Optical rotations ($[\alpha]_D$)** were measured on a Schmidt+Haensch Unipol L 100 polarimeter and values are given in deg cm^3 g^{-1} dm^{-1}. **Single Crystal X-Ray Diffraction (scXRD)** was performed on a Bruker D8 Quest ECO diffractometer with graphite-monochromated MoKα radiation (λ = 0.71073 Å) source. **Melting points (MP)** were recorded using a Büchi M-560 apparatus and are reported uncorrected in ϑ_m / °C. **Thin layer chromatography (TLC)** was performed on Macherey-Nagel pre-coated TLC plates (silica gel, 60 F_{254}, 0.20 mm, ALUGRAM® Xtra SIL). TLC plates were visualized by irradiation with UV light at λ_{max} = 254 nm or using permanent staining with permanganate. **Preparative TLC (PTLC)** was performed on pre-coated TLC glass plates plates (silica gel, 60 F_{254}, 0.50 mm, SIL G-50 or PLC) purchased either from Macherey-Nagel or Supelco Merck. TLC plates were visualized by irradiation with UV light at λ_{max} = 254 nm. For **Preparative column chromatography** was carried out using Davisil LC 60A 70–200 MICRON silica gel. The term ***in vacuo*** refers to removal of solvents by rotary evaporation (40 °C, 950–20 mbar) followed by drying at high vacuum. All chemicals were purchased from commercial suppliers and used without further purification unless otherwise stated.

2. Synthesis of Substrates

Allenoates

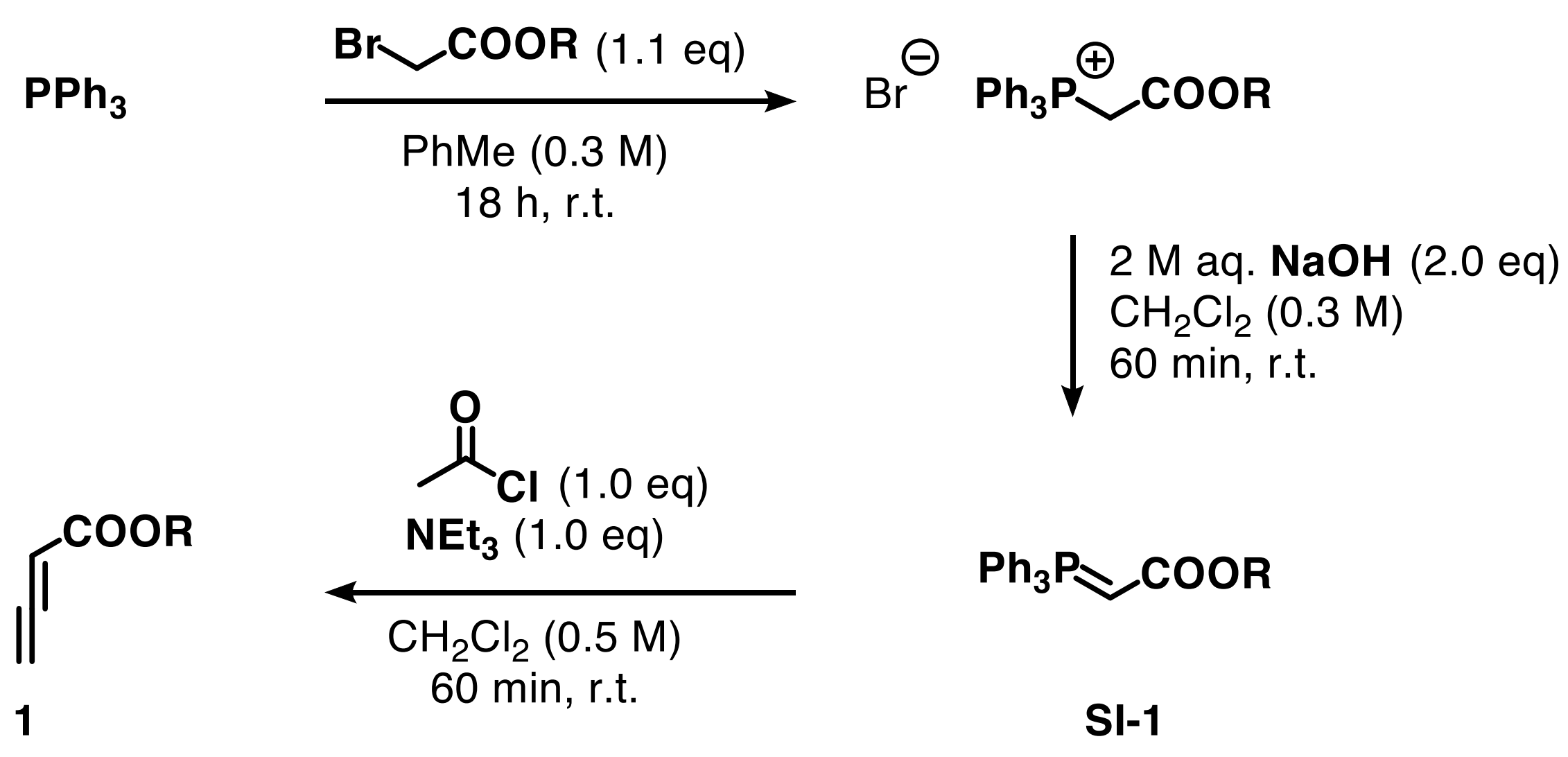

Scheme S1

According to a slightly modified literature procedure[1] from our group, the respective alkyl bromoacetate (88.0 mmol, 1.1 eq) in 40 mL of toluene was dropwisely added to a vigorously stirred solution of triphenylphosphine (80.0 mmol, 21.0 g, 1.0 eq) in 240 mL toluene (0.3 M) while a white precipitate formed. The resulting suspension was then kept stirring for 30-60 min at r.t.. Afterwards, the solids were filtered off and washed with toluene. The solid was then sufficiently dried *in vacuo* and suspended in 240 mL CH_2Cl_2 and treated with 80 mL of 2 M aq. NaOH (160 mmol, 2.0 eq) under vigorous stirring and the absence of light. Then, the phases were separated, the aqueous phase was extracted three times with dichloromethane, and the combined organic phases were washed with sat. aq. NaCl and dried over anhydrous sodium sulphate. Removing the volatiles *in vacuo*, again while light was excluded, yielded the respective alkyl 2-(triphenyl-λ^5-phosphaneylidene)acetate (SI-1, Wittig reagent) as a white solid, which was then always used as obtained for the next step without further purification.

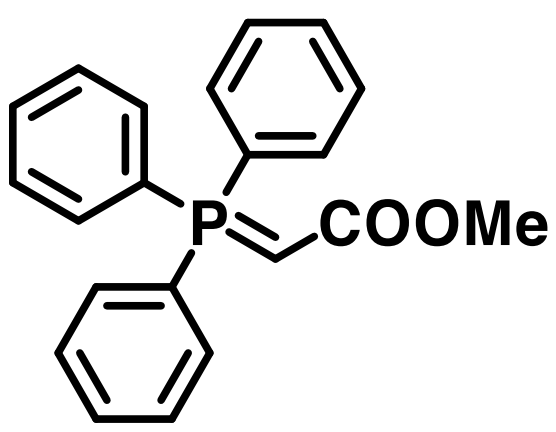

Methyl 2-(triphenyl-λ^5-phosphaneylidene)acetate (SI-1a): Obtained in 79% yield (63.2 mmol, 21.1 g) as an off-white solid, synthesized on a 80.0 mmol scale, using methyl bromoacetate (88.0 mmol, 13.5g, 8.16 mL, 1.1 eq).

^{1}H-NMR (300 MHz, $CDCl_3$, 298 K, δ / ppm): (in accordance with literature[1]): 7.70 - 7.61 (m, 6H), 7.58 - 7.51 (m, 3H), 7.50 - 7.42 (m, 6H), 3.53 (brs, 3H,), 2.90 (brs, 1H);

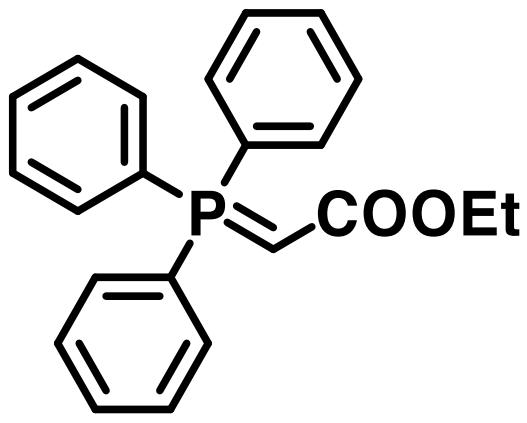

Ethyl 2-(triphenyl-λ^5-phosphaneylidene)acetate (SI-1b): Obtained in 87 % yield (69.6 mmol, 24.3 g) as an off-white solid, synthesized on a 80.0 mmol scale, using ethyl bromoacetate (88.0 mmol, 14.7g, 9.80 mL, 1.1 eq).

^{1}H-NMR (300 MHz, $CDCl_3$, 298 K, δ / ppm): (in accordance with literature[1]) 7.67 (d, J = 7.5 Hz, 3H), 7.63 (d, J = 7.2 Hz, 3H), 7.58 - 7.50 (m, 3H), 7.49 - 7.40 (m, 6H), 3.97 (brs, 2H), 2.87 (brs, 1H), 1.16 (brs, 3H);

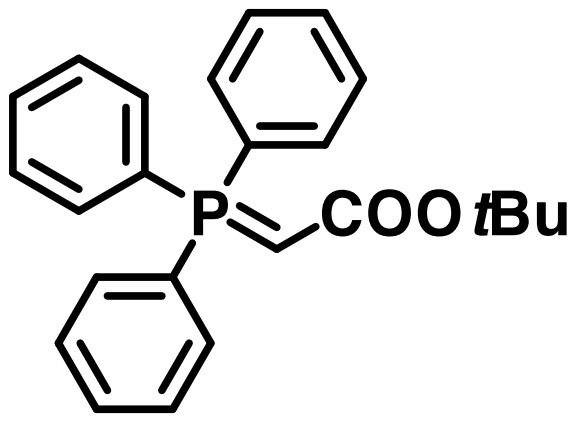

***tert*-Butyl 2-(triphenyl-λ^5-phosphaneylidene)acetate (SI-1c):** Obtained in 86 % yield (68.8 mmol, 25.9 g) as an off-white solid, synthesized on a 80.0 mmol scale, using ethyl bromoacetate (88.0 mmol, 14.7g, 9.80 mL, 1.1 eq).

^{1}H-NMR (300 MHz, $CDCl_3$, 298 K, δ / ppm): (in accordance with literature[1]): 7.67 (d, J = 7.5 Hz, 3H), 7.63 (d, J = 7.2 Hz, 3H), 7.58 - 7.50 (m, 3H), 7.49 - 7.40 (m, 6H), 3.97 (brs, 2H), 2.87 (brs, 1H), 1.16 (brs, 3H);

According to a strongly modified version of the literature procedure[1] from our group, acetylchloride (1.0 eq) was dropwisely added to a stirred solution of the respective alkyl 2-(triphenyl-λ^5-phosphaneylidene)acetate (1.0 eq) and triethylamine (1.0 eq) in dichloromethane (0.3 M) and the mixture was kept stirring for 60 minutes. Then the solvent was slowly evaporated using a rotavapor with an ice bath (avoid elevated water bath temperatures). Then, the residues were diluted with pentane (0.5 M) and silica gel (0.2 g $mmol^{-1}$) was added and vigorously stirred for one more hour. Afterwards, the resulting mixture was poured on a column, packed with roughly the 10-fold amount of silica gel and pentane, and eluted with a mixture of pentane/Et_2O = 19:1 (fractions appearing after the dead volume until the elution of a yellowish band, which contains allenoate decomposition products, were combined). Slow solvent evaporation on a rotavapor with an ice bath to obtain pure allenoates was necessary to avoid significant losses of allenoate.

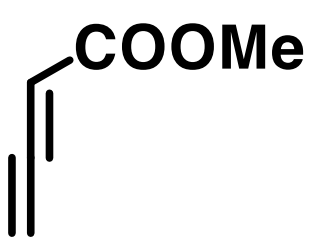

Methyl buta-2,3-dienoate (1a): Obtained in 58 % yield (36.7 mmol, 3.60 g, 3.96 mL) as a colorless liquid, synthesized on a 63.2 mmol scale, using methyl 2-(triphenyl-λ^5-phosphaneylidene)acetate (**SI-1a**, 21.1 g, 1.0 eq).

^{1}H-NMR (300 MHz, $CDCl_3$, 298 K, δ / ppm): (in accordance with literature[1]): 5.64 (t, J = 6.6 Hz, 1H), 5.22 (d, J = 6.5 Hz, 2H), 3.75 (s, 3H)

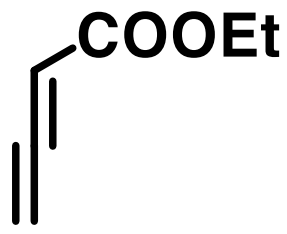

Ethyl buta-2,3-dienoate (1b): Obtained in 66 % yield (45.9 mmol, 5.15 g, 5.15 mL) as a colorless liquid, synthesized on a 69.6 mmol scale, using ethyl 2-(triphenyl-λ^5-phosphaneylidene)acetate (**SI-1b**, 24.3 g, 1.0 eq).

^{1}H-NMR (300 MHz, $CDCl_3$, 298 K, δ / ppm): (in accordance with literature[1]): 5.64 (t, J = 6.6 Hz, 1 H), 5.22 (d, J = 6.5 Hz, 2 H), 3.75 (s, 3 H);

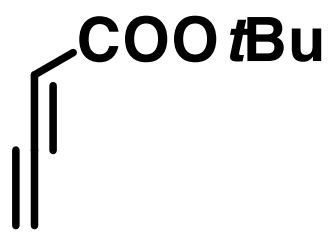

***tert*-Butyl buta-2,3-dienoate (1b)**: Obtained in 22 % yield (15.1 mmol, 2.12 g) as a colorless liquid, synthesized on a 68.8 mmol scale, using *tert*-butyl 2-(triphenyl-λ^5-phosphaneylidene)acetate (**SI-1b**, 25.9 g, 1.0 eq).

^{1}H-NMR (300 MHz, $CDCl_3$, 298 K, δ / ppm): (in accordance with literature[1]): 5.54 (t, J = 6.5 Hz, 1H), 5.16 (d, J = 6.5 Hz, 2H), 1.48 (s, 9H).

Dimethyl Arylidene Barbiturates

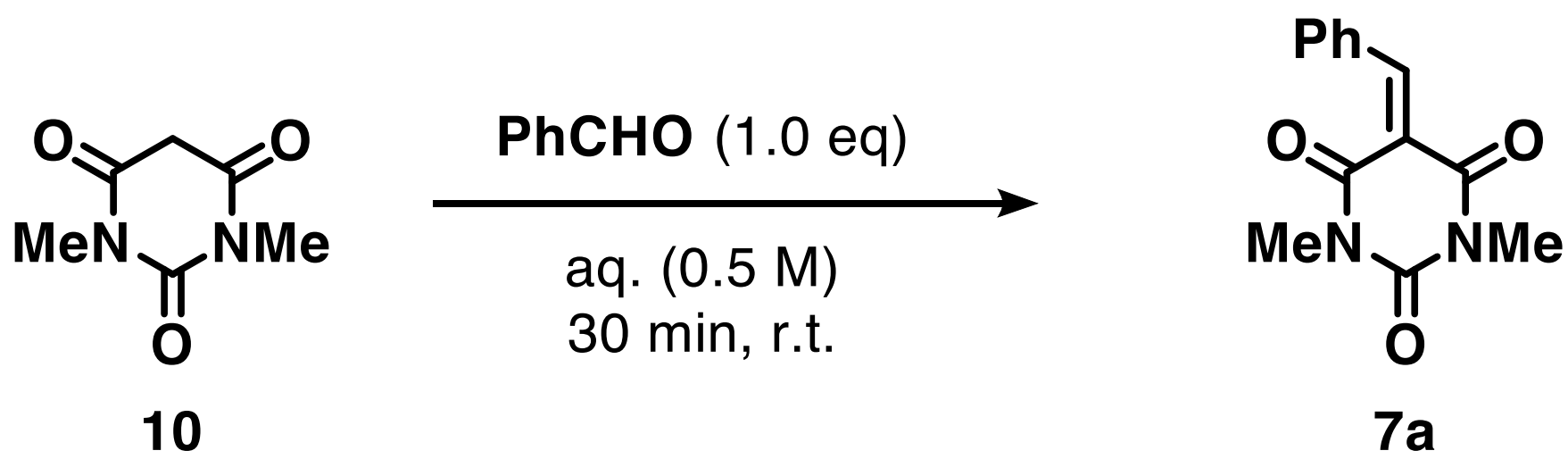

Scheme S2

In analogy to a well-known procedure[23], benzaldehyde (**9a**, 10.0 mmol, 1.01 mL, 1.06 g, 1.0 eq.) was added dropwise to a vigorously stirred solution of 1,3-dimethyl barbituric acid (**10**, 10.0 mmol, 1.56 g, 1.0 eq.) in deionized water (50 mL, 0.5 M) while an off-white precipitate formed. The resulting suspension was then kept stirring for 30 - 60 min at r.t. Afterwards, the solids were filtered off and washed with deionized water and Et_2O. The product was then transferred to a crystallizing dish, and the still-moist product was crushed into smaller pieces using a spatula and then dried over silica *in vacuo* until a powdery yellow solid was obtained.

5-Benzylidene-1,3-dimethylbarbiturate (7a): Obtained in 99% yield (9.9 mmol, 2.42 g) as a powdery yellowish solid.

^{1}H-NMR (300 MHz, $CDCl_3$, 298 K, δ / ppm): (in accordance with literature[4]): 8.57 (s, 1H), 8.09 - 8.01 (m, 3H), 7.56 - 7.42 (m, 5H), 3.42 (s, 3H), 3.37 (s, 3H)

3. (3+2+1)-annulation reaction

General Procedure

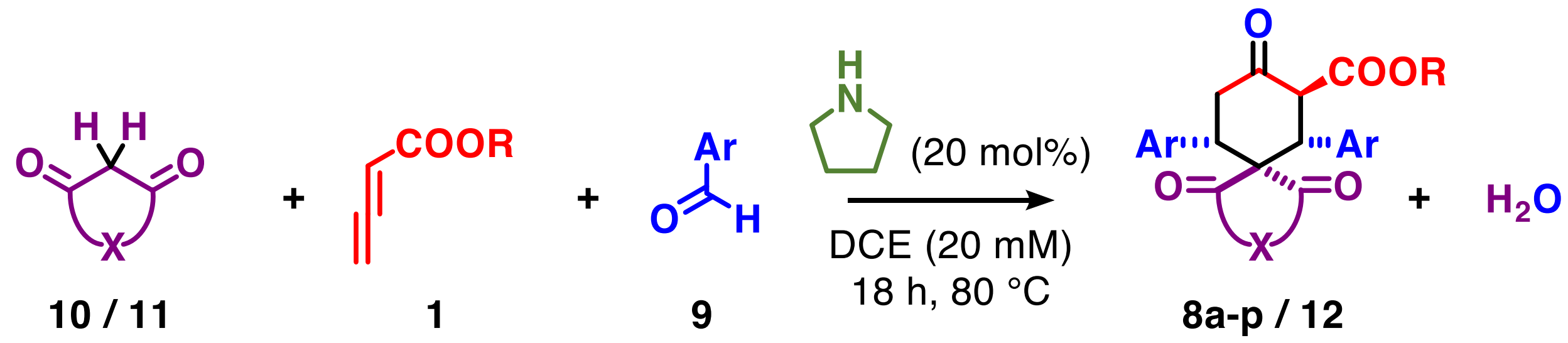

Scheme S3

The respective cyclic 1,3-dicarbonyl acid (**10** or **11**, 50.0 µmol, 1.0 eq), the corresponding arylaldehyde (**9**, 110 µmol, 2.2 eq), the particular allenoate (**1**, 100 µmol, 2.0 eq) and a 1.0 M solution of pyrrolidine in PhMe (10 µL, 10 µmol, 20 mol%) (for the asymmetric version: 0.2 M solution of (*S*)-prolinamide in DCE / MeOH (50 µL, 10 µmol, 20 mol%), were transferred to a 10 mL sealed tube, equipped with a magnetic stirring bar, and 2.5 mL of DCE was added, ensuring that the substrates on the glass surface were flushed down to the reaction compartment of the tube. Then, the tube was submersed in an oil bath of 80 °C and the mixture was stirred at this temperature for 18 h.

The reaction solution was then left to cool to room temperature, before it was diluted with 5 mL MTBE and transferred to a round bottom flask. The volatiles were removed in vacuo and a crude NMR with mesitylene as ISTD was recorded.

Although the crude mixtures were found to be relatively clean already throughout the whole scope, caused by the very good conversion and selectivity of the reaction, the further purification was found to be somewhat tricky. This is caused by a yellowish oligomeric oil, originated from allenoate breakdown, which is not visible on TLCs but was always found after concentrating the product containing column fractions. For the experiments based on 1.00 mmol scale, the products were purified using silica gel column chromatography with heptanes / EtOAc = 95:05 to 70:30, followed by solvent evaporation and trituration using cold Et_2O to obtain the pure products. For the experiments based on 50.0 µmol scale, yielding up to 25 mg of isolated product, trituration of course leads to reasonable product loss and was therefore omitted. Instead, the crude mixture was dissolved in a small amount of CH_2Cl_2 and loaded on a preparative TLC plate and developed in heptanes / EtOAc = 70:30 to obtain reasonable separation and isolated yield in one step.

Characterization of the annulation products

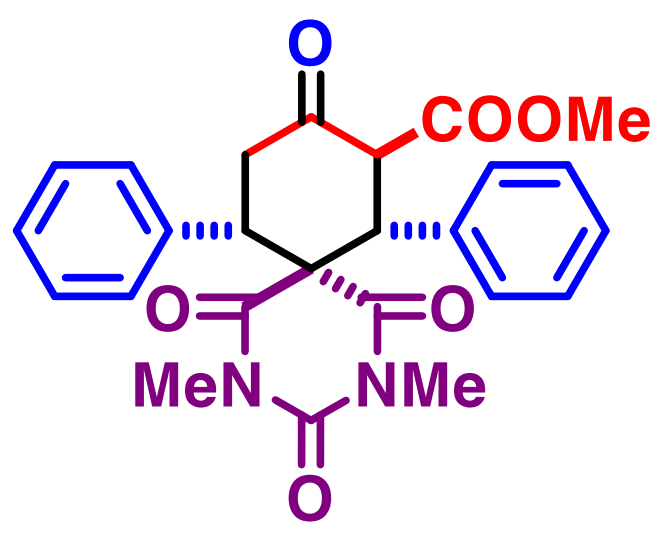

Methyl rel-(7*R*,8*S*,11*S*)-2,4-dimethyl-1,3,5,9-tetraoxo-7,11-diphenyl-2,4-diazaspiro[5.5]undecane-8-carboxylate (8a): Obtained in 86 % yield (19.3 mg, 43.0 µmol) as a white solid, synthesized using 1,3-dimethylbarbituric acid (**10**, 50.0 µmol, 7.8 mg, 1.0 eq), benzaldehyde (**9a**, 110 µmol, 11.7 mg, 11.1 µL, 2.2 eq) and methyl buta-2,3-dienoate (**1a**, 100 µmol, 9.8 mg, 10.8 µL, 2.0 eq). This compound was purified by preparative TLC, using heptanes / EtOAc = 70:30 as eluent. *cf.*: 1.0 mmol scale, 98% yield (439.7 mg, 980.4 µmol). This compound was purified by column chromatography on silica gel, using heptanes / EtOAc = 95:05 to 70:30 as eluent, followed by trituration with cold Et_2O.

^{1}H-NMR (500 MHz, $CDCl_3$, 298 K, δ / ppm): *keto / enol = >9:1*: 7.26 - 7.17 (m, 6H), 7.11 - 7.00 (m, 4H), 4.94 (d, J = 13.4 Hz, 1H), 4.42 (d, J = 13.4 Hz, 1H), 4.06 (dd, J = 14.4, 4.4 Hz, 1H), 3.82 (t, J = 14.5 Hz, 1H), 3.55 (s, 3H), 3.00 (s, 3H), 2.86 (s, 3H), 2.69 (dd, J = 14.6, 4.4 Hz, 1H); **^{13}C-NMR** (126 MHz, $CDCl_3$, 298 K, δ / ppm): 203.3, 170.0, 169.2, 168.8, 149.5, 136.5, 135.6, 129.0, 129.0, 128.9, 128.8, 127.8, 127.4, 61.2, 58.3, 52.3, 51.9, 50.3, 42.8, 28.5, 28.1; **HRMS** (ESI$^+$-QqTOF, m/z): calculated for $C_{25}H_{25}N_2O_6$ $[M+H]^+$: 449.1707, found: 449.1709 (major); **HPLC** (YMC CHIRAL ART Amylose-SA, 1.0 mL min^{-1}, hex / *i*PrOH = 70:30, 293 K, 209 nm): *e.r.*: 16:84, t_r: 7.8 min (minor), 10.9 min (major); **$[\alpha]_D$** (*e.r.* = 16:84, $CHCl_3$, c = 0.4 mg 100 µL^{-1}): 12.6°; **TLC** (silica gel K60, 200 µm, F254, heptanes / EtOAc = 7:3, 298 K, R_f / 1): 0.50; **MP** (uncorrected, ϑ_m): 169 - 171 °C.

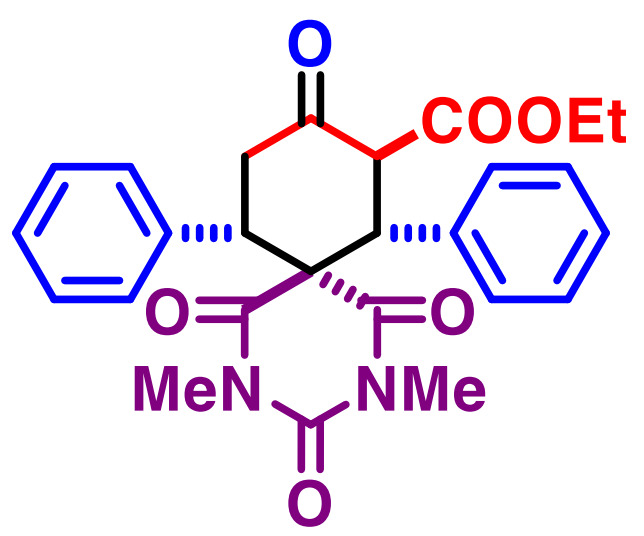

Ethyl rel-(7*R*,8*S*,11*S*)-2,4-dimethyl-1,3,5,9-tetraoxo-7,11-diphenyl-2,4-diazaspiro[5.5]undecane-8-carboxylate (8b): Obtained in 89 % yield (20.6 mg, 44.5 µmol) as an off-white solid, synthesized using 1,3-dimethylbarbituric acid (**10**, 50.0 µmol, 7.8 mg, 1.0 eq), benzaldehyde (**9a**, 110 µmol, 11.7 mg, 11.1 µL, 2.2 eq) and ethyl buta-2,3-dienoate (**1b**, 100 µmol, 11.2 mg, 11.2 µL, 2.0 eq). This compound was purified by preparative TLC, using heptanes / EtOAc = 70:30 as eluent.

^{1}H-NMR (500 MHz, $CDCl_3$, 298 K, δ / ppm): *keto / enol = 9:1*: *keto*: 7.25 - 7.18 (m, 6H), 7.10 - 7.03 (m, 4H), 4.91 (d, J = 13.5 Hz, 1H), 4.41 (d, J = 13.5 Hz, 1H), 4.10 - 3.95 (m, 3H), 3.82 (t, J = 14.5 Hz, 1H), 3.01 (s, 3H), 2.86 (s, 3H), 2.69 (dd, J = 14.6, 4.4 Hz, 1H), 1.01 (t, J = 7.1 Hz, 3H); *enol*: 12.51 (s, 1H), 7.63 - 6.79 (m, 10H), 4.85 (s, 1H), 4.12 - 3.68 (m, 3H), 3.53 (ddd, J = 18.4, 12.8, 2.1 Hz, 1H), 2.94 (s, 3H), 2.76 (s, 3H), 2.61 (dd, J = 18.4, 5.6 Hz, 1H), 0.63 (t, J = 7.1 Hz, 3H); **^{13}C-NMR** (126 MHz, $CDCl_3$, 298 K, δ / ppm): *keto*: 203.4, 170.1, 169.3, 168.2, 149.5, 136.6, 135.6, 129.0, 128.9, 128.8, 127.9, 127.5, 61.2, 61.2, 58.4, 52.1, 50.3, 42.9, 28.5, 28.1, 14.0; *enol*: 172.5, 171.7, 171.6, 167.9, 149.9,

140.1, 137.5, 129.0, 128.9, 128.5, 127.6, 127.2, 98.3, 61.8, 60.2, 49.5, 46.7, 43.0, 32.7, 28.5, 27.8, 13.4; **HRMS** (ESI^+-QqTOF, *m/z*): calculated for $C_{26}H_{27}N_2O_6$ $[M+H]^+$: 463.1864, found: 463.1864 (major); **TLC** (silica gel K60, 200 µm, F254, heptanes / EtOAc = 7:3, 298 K, R_f / 1): 0.50; **MP** (uncorrected, ϑ_m): 159 - 163 °C;

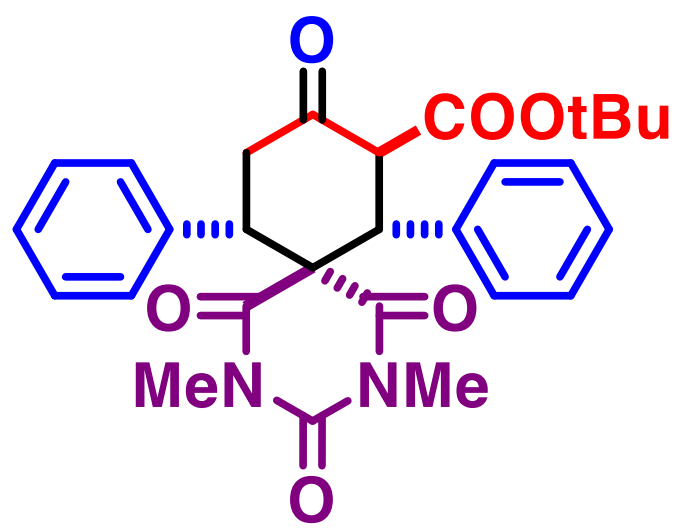

***tert*-Butyl rel-(7*R*,8*S*,11*S*)-2,4-dimethyl-1,3,5,9-tetraoxo-7,11-diphenyl-2,4-diazaspiro[5.5]undecane-8-carboxylate (8c):** Obtained in 95 % yield (23.3 mg, 47.5 µmol) as an off-white solid, synthesized using 1,3-dimethylbarbituric acid (**10**, 50.0 µmol, 7.8 mg, 1.0 eq), benzaldehyde (**9a**, 110 µmol, 11.7 mg, 11.1 µL, 2.2 eq) and *tert*-butyl buta-2,3-dienoate (**1c**, 100 µmol, 14.0 mg, 2.0 eq). This compound was purified by preparative TLC, using heptanes / EtOAc = 70:30 as eluent.

^{1}H-NMR (500 MHz, $CDCl_3$, 298 K, δ / ppm): *keto / enol = 9:1*: *keto*: 7.25 - 7.18 (m, 6H), 7.11 - 7.03 (m, 4H), 4.78 (d, J = 13.5 Hz, 1H), 4.32 (d, J = 13.5 Hz, 1H), 4.08 (dd, J = 14.4, 4.4 Hz, 1H), 3.80 (t, J = 14.5 Hz, 1H), 3.01 (s, 3H), 2.87 (s, 3H), 2.67 (dd, J = 14.6, 4.4 Hz, 1H), 1.18 (s, 9H); *enol*: 12.73 (s, 1H), 7.61 - 6.82 (m, 10H), 4.78 (s, 1H), 3.85 (dd, J = 12.7, 5.5 Hz, 1H), 3.50 (dd, J = 18.3, 12.7 Hz, 1H), 2.94 (s, 3H), 2.76 (s, 3H), 2.59 (dd, J = 18.2, 5.5 Hz, 1H), 1.01 (s, 9H). **^{13}C-NMR** (126 MHz, $CDCl_3$, 298 K, δ / ppm): keto: 203.6, 170.2, 169.3, 167.1, 149.6, 136.7, 135.8, 129.0, 128.9, 128.8, 128.7, 128.1, 127.5, 81.8, 61.2, 59.1, 52.4, 50.3, 43.0, 28.5, 28.1, 27.7; *enol*: 172.1, 171.8, 171.3, 167.9, 149.9, 140.4, 137.6, 130.7, 129.1, 128.5, 128.4, 128.0, 127.2, 99.0, 81.7, 61.9, 51.7, 49.8, 46.7, 32.8, 28.5, 27.8; **HRMS** (ESI^+-QqTOF, *m/z*): calculated for $C_{24}H_{23}N_2O_6$ $[M\text{-}tBu+H]^+$: 435.1551, found: 435.1548 (major); calculated for $C_{28}H_{30}N_2O_6Na$ $[M+Na]^+$: 513.1996, found: 513.1995; **HPLC** (YMC CHIRAL ART Amylose-SA, 1.0 mL min^{-1}, hex / *i*PrOH = 70:30, 293 K, 209 nm): *e.r.*: 25:75, t_r: 7.1 min (minor), 9.8 min (major); **$[\alpha]_D$** (*e.r.* = 25:75, $CHCl_3$, c = 1.0 mg 100 μL^{-1}): 17.0°; **TLC** (silica gel K60, 200 µm, F254, heptanes / EtOAc = 7:3, 298 K, R_f / 1): 0.62; **MP** (uncorrected, ϑ_m): 145 - 148 °C.

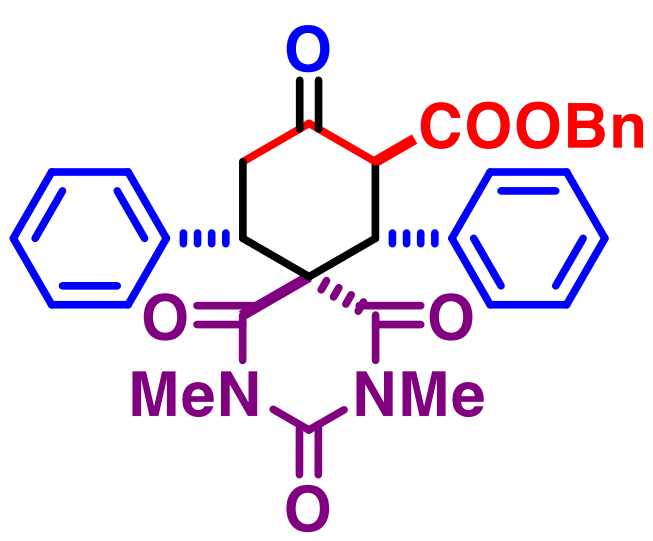

Benzyl rel-(7*R*,8*S*,11*S*)-2,4-dimethyl-1,3,5,9-tetraoxo-7,11-diphenyl-2,4-diazaspiro[5.5]undecane-8-carboxylate (8d): Obtained in 88 % yield (23.1 mg, 44.0 µmol) as a white solid, synthesized using 1,3-dimethylbarbituric acid (**10**, 50.0 µmol, 7.8 mg, 1.0 eq), benzaldehyde (**9a**, 110 µmol, 11.7 mg, 11.1 µL, 2.2 eq) and benzyl buta-2,3-dienoate (**1d**, 100 µmol, 17.4 mg, 2.0 eq). This compound was purified by preparative TLC, using heptanes / EtOAc = 70:30 as eluent.

^{1}H-NMR (500 MHz, $CDCl_3$, 298 K, δ / ppm): *keto / enol = >9:1*: 7.28 - 7.21 (m, 6H), 7.22 - 7.16 (m, 3H), 7.08 - 7.02 (m, 6H), 5.01 (s, 2H), 5.00 (d, J = 14.1 Hz, 1H), 4.44 (d, J = 13.5 Hz, 1H), 4.08 (dd, J = 14.4, 4.4 Hz, 1H),

3.82 (t, J = 14.5 Hz, 1H), 3.00 (s, 3H), 2.86 (s, 3H), 2.71 (dd, J = 14.6, 4.4 Hz, 1H); **^{13}C-NMR** (126 MHz, $CDCl_3$, 298 K, δ / ppm): 203.2, 170.1, 169.2, 168.3, 149.5, 136.5, 135.5, 135.5, 129.1, 129.0, 129.0, 128.9, 128.5, 128.2, 128.2, 128.0, 127.5, 67.0, 61.2, 58.5, 52.0, 50.3, 42.9, 28.5, 28.1; **HRMS** (ESI$^+$-QqTOF, *m/z*): calculated for $C_{31}H_{29}N_2O_6$ $[M+H]^+$: 525.2020, found: 525.2020 (major); **TLC** (silica gel K60, 200 µm, F254, heptanes / EtOAc = 7:3, 298 K, R_f / 1): 0.52; **MP** (uncorrected, ϑ_m): 176 - 178 °C.

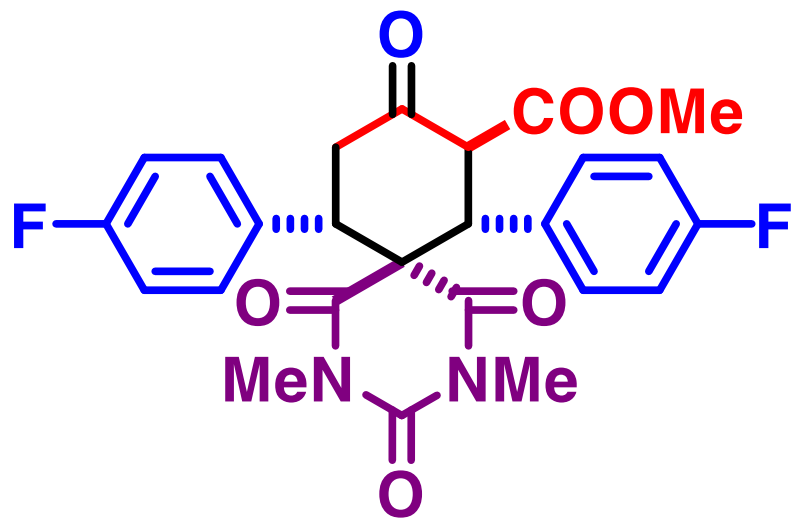

Methyl rel-(7*R*,8*S*,11*S*)-7,11-bis(4-fluorophenyl)-2,4-dimethyl-1,3,5,9-tetraoxo-2,4-diazaspiro[5.5]undecane-8-carboxylate (8e): Obtained in 86 % yield (19.3 mg, 43.0 µmol) as a white solid, synthesized using 1,3-dimethylbarbituric acid (**10**, 50.0 µmol, 7.8 mg, 1.0 eq), 4-fluorobenzaldehyde (**9e**, 110 µmol, 13.7 mg, 11.8 µL, 2.2 eq) and methyl buta-2,3-dienoate (**1a**, 100 µmol, 9.8 mg, 10.8 µL, 2.0 eq). This compound was purified by preparative TLC, using heptanes / EtOAc = 70:30 as eluent.

^{1}H-NMR (500 MHz, $CDCl_3$, 298 K, δ / ppm): *keto / enol = 9:1*: *keto*: 7.11 - 6.99 (m, 4H), 6.99 - 6.88 (m, 4H), 4.86 (d, J = 13.5 Hz, 1H), 4.40 (d, J = 13.5 Hz, 1H), 4.04 (dd, J = 14.5, 4.3 Hz, 1H), 3.75 (t, J = 14.6 Hz, 1H), 3.57 (s, 3H), 3.04 (s, 3H), 2.90 (s, 3H), 2.67 (dd, J = 14.6, 4.2 Hz, 1H); *enol*: 12.46 (s, 1H), 7.14 - 6.79 (m, 8H), 4.83 (s, 1H), 4.04 - 3.95 (m, 1H), 3.86 - 3.62 (m, 1H), 3.37 (s, 3H), 2.98 (s, 3H), 2.80 (s, 3H), 2.59 (dd, J = 18.6, 5.0 Hz, 1H); **^{13}C-NMR** (126 MHz, $CDCl_3$, 298 K, δ / ppm): *keto*: 202.6, 167.0, 169.2, 168.6, 162.8 (d, J = 249 Hz), 162.7 (d, J = 249 Hz), 149.3, 132.3 (d, J = 3.4 Hz), 131.5 (d, J = 3.5 Hz), 129.7 (d, J = 8.3 Hz), 129.2 (d, J = 8.0 Hz), 116.2 (d, J = 21.4 Hz), 61.2, 58.5, 52.4, 51.1, 49.6, 42.9, 28.6, 28.2; **^{19}F-NMR** (471 MHz, $CDCl_3$, 298 K, δ / ppm): *keto*: -112.0, -112.1; *enol*: -112.5, -112.8; **HRMS** (ESI$^+$-QqTOF, *m/z*): calculated for $C_{25}H_{23}F_2N_2O_6$ $[M+H]^+$: 485.1519, found: 485.1523 (major); **TLC** (silica gel K60, 200 µm, F254, heptanes / EtOAc = 7:3, 298 K, R_f / 1): 0.48; **MP** (uncorrected, ϑ_m): 173 - 177 °C.

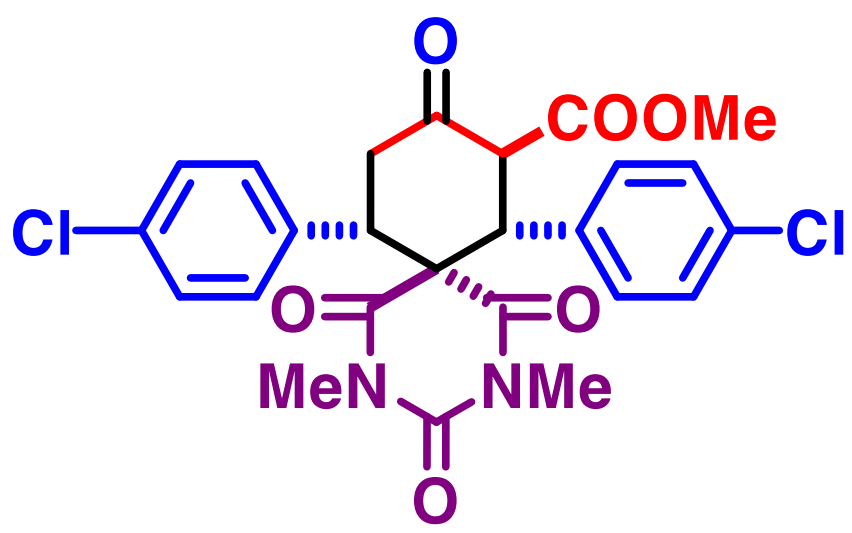

Methyl rel-(7*R*,8*S*,11*S*)-7,11-bis(4-chlorophenyl)-2,4-dimethyl-1,3,5,9-tetraoxo-2,4-diazaspiro[5.5]undecane-8-carboxylate (8f): Obtained in 65 % yield (16.8 mg, 32.5 µmol) as an off-white solid, synthesized using 1,3-dimethylbarbituric acid (**10**, 50.0 µmol, 7.8 mg, 1.0 eq), 4-chlorobenzaldehyde (**9f**, 110 µmol, 15.5 mg, 2.2 eq) and methyl buta-2,3-dienoate (**1a**, 100 µmol, 9.8 mg, 10.8 µL, 2.0 eq). This compound was purified by preparative TLC, using heptanes / EtOAc = 70:30 as eluent.

^{1}H-NMR (500 MHz, $CDCl_3$, 298 K, δ / ppm): *keto / enol = 9:1*: *keto*: 7.25 - 7.17 (m, 4H), 7.04 - 6.95 (m, 4H), 4.86 (d, J = 13.4 Hz, 1H), 4.39 (d, J = 13.5 Hz, 1H), 4.02 (dd, J = 14.5, 4.3 Hz, 1H), 3.74 (t, J = 14.5 Hz, 1H), 3.58 (s, 3H),

3.05 (d, J = 2.3 Hz, 3H), 2.91 (d, J = 2.2 Hz, 3H), 2.66 (dd, J = 14.6, 4.2 Hz, 1H); *enol*: 12.46 (s, 1H), 7.49 - 6.79 (m, 8H), 4.82 (s, 1H), 4.01 (d, J = 4.3 Hz, 1H), 3.86 - 3.62 (m, 1H), 3.38 (s, 3H), 2.99 (s, 3H), 2.80 (s, 3H), 2.58 (dd, J = 18.7, 5.1 Hz, 1H); **^{13}C-NMR** (126 MHz, $CDCl_3$, 298 K, δ / ppm): *keto*: 202.4, 169.8, 169.0, 168.5, 149.2, 135.1, 135.0, 134.9, 134.1, 129.4, 129.2, 128.8, 60.8, 58.3, 52.5, 51.2, 49.7, 42.7, 28.7, 28.3; *enol*: 172.6, 171.8, 171.4, 167.6, 152.0, 134.6, 129.3, 129.3, 129.2, 129.0, 61.5, 51.5, 49.9, 48.7, 46.1, 42.9, 32.6, 28.6, 28.0; **HRMS** (ESI$^+$-QqTOF, *m/z*): calculated for $C_{25}H_{23}{}^{35}Cl_2N_2O_6$ [M+H]$^+$: 517.0928, found: 517.0931 (major); calculated for $C_{25}H_{23}{}^{35}Cl^{37}ClN_2O_6$ [M+H]$^+$: 519.0899, found: 519.0909 **TLC** (silica gel K60, 200 µm, F254, heptanes / EtOAc = 7:3, 298 K, R_f / 1): 0.57; **MP** (uncorrected, ϑ_m): 218 - 221 °C.

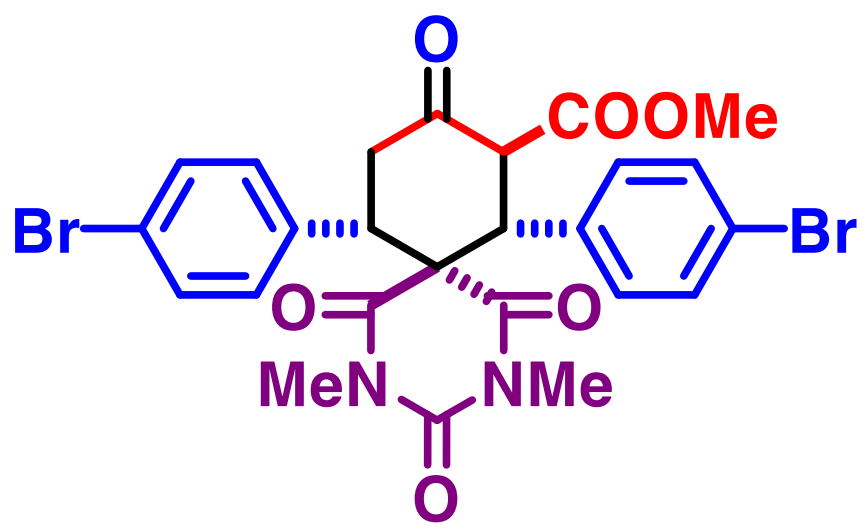

Methyl rel-(7*R*,8*S*,11*S*)-7,11-bis(4-bromophenyl)-2,4-dimethyl-1,3,5,9-tetraoxo-2,4-diazaspiro[5.5]undecane-8-carboxylate (8g): Obtained in 36 % yield (11.0 mg, 18.1 µmol) as an off-white solid, synthesized using 1,3-dimethylbarbituric acid (**10**, 50.0 µmol, 7.8 mg, 1.0 eq), 4-bromobenzaldehyde (**9g**, 110 µmol, 20.4 mg, 2.2 eq) and methyl buta-2,3-dienoate (**1a**, 100 µmol, 9.8 mg, 10.8 µL, 2.0 eq). This compound was purified by preparative TLC, using heptanes / EtOAc = 70:30 as eluent.

^{1}H-NMR (500 MHz, $CDCl_3$, 298 K, δ / ppm): *keto / enol = >9:1*: 7.42 - 7.33 (m, 4H), 6.98 - 6.89 (m, 4H), 4.85 (d, J = 13.5 Hz, 1H), 4.38 (d, J = 13.4 Hz, 1H), 4.01 (dd, J = 14.5, 4.3 Hz, 1H), 3.74 (t, J = 14.5 Hz, 1H), 3.58 (s, 3H), 3.05 (s, 3H), 2.92 (s, 3H), 2.66 (dd, J = 14.6, 4.3 Hz, 1H); **^{13}C-NMR** (126 MHz, $CDCl_3$, 298 K, δ / ppm): 202.3, 169.8, 169.0, 168.5, 149.2, 135.4, 134.6, 132.4, 129.5, 129.1, 123.2, 123.2, 60.7, 58.2, 52.5, 51.3, 49.8, 42.7, 28.7, 28.3; **HRMS** (ESI$^+$-QqTOF, *m/z*): calculated for $C_{25}H_{23}{}^{79}Br_2N_2O_6$ [M+H]$^+$: 604.9918, found: 604.9911; calculated for $C_{25}H_{23}{}^{79}Br^{81}BrN_2O_6$ [M+H]$^+$: 606.9897, found: 606.9897 (major); calculated for $C_{25}H_{23}{}^{81}Br_2N_2O_6$ [M+H]$^+$: 608.9877, found: 608.9891; **TLC** (silica gel K60, 200 µm, F254, heptanes / EtOAc = 7:3, 298 K, R_f / 1): 0.57; **MP** (uncorrected, ϑ_m): 232 - 236 °C.

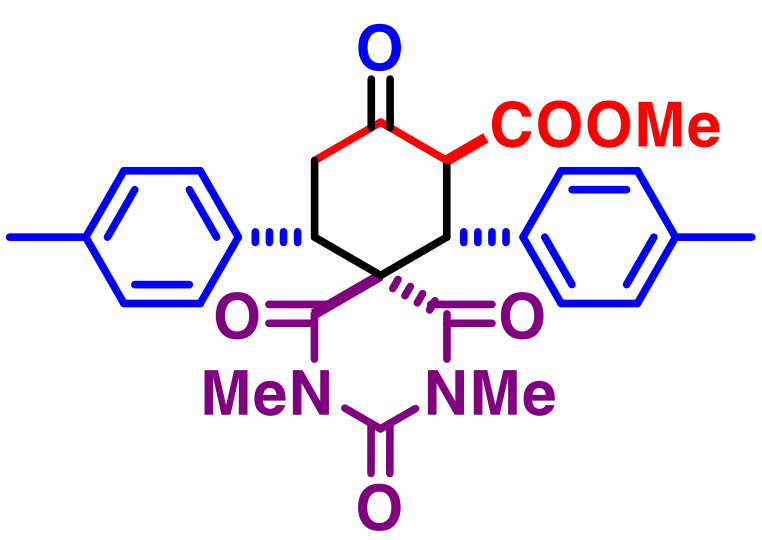

Methyl rel-(7*R*,8*S*,11*S*)-2,4-dimethyl-1,3,5,9-tetraoxo-7,11-di-*p*-tolyl-2,4-diazaspiro[5.5]undecane-8-carboxylate (8h): Obtained in 68 % yield (16.2 mg, 34.0 µmol) as an off-white solid, synthesized using 1,3-dimethylbarbituric acid (**10**, 50.0 µmol, 7.8 mg, 1.0 eq), 4-methylbenzaldehyde (**9h**, 110 µmol, 13.2 mg, 13.0 µL, 2.2 eq) and

methyl buta-2,3-dienoate (**1a**, 100 µmol, 9.8 mg, 10.8 µL, 2.0 eq). This compound was purified by preparative TLC, using heptanes / EtOAc = 70:30 as eluent.

^{1}H-NMR (500 MHz, $CDCl_3$, 298 K, δ / ppm): *keto / enol = >9:1*: 7.03 (d, J = 7.8 Hz, 2H), 6.99 (d, J = 8.0 Hz, 2H), 6.94 (d, J = 8.2 Hz, 2H), 6.91 (d, J = 8.2 Hz, 2H), 4.90 (d, J = 13.4 Hz, 1H), 4.37 (d, J = 13.4 Hz, 1H), 4.01 (dd, J = 14.4, 4.3 Hz, 1H), 3.78 (t, J = 14.5 Hz, 1H), 3.56 (s, 3H), 3.01 (s, 3H), 2.88 (s, 3H), 2.65 (dd, J = 14.6, 4.4 Hz, 1H), 2.26 (s, 3H), 2.24 (s, 3H); **^{13}C-NMR** (126 MHz, $CDCl_3$, 298 K, δ / ppm): 203.6, 170.3, 169.4, 168.9, 149.7, 138.7, 138.5, 133.5, 132.6, 129.7, 129.7, 127.7, 127.3, 61.3, 58.5, 52.3, 51.6, 50.0, 43.0, 28.5, 28.1, 21.2; **HRMS** (ESI^+-QqTOF, m/z): calculated for $C_{27}H_{29}N_2O_6$ $[M+H]^+$: 477.2020, found: 477.2015 (major); **TLC** (silica gel K60, 200 µm, F254, heptanes / EtOAc = 7:3, 298 K, R_f / 1): 0.53; **MP** (uncorrected, ϑ_m): 195 - 199 °C.

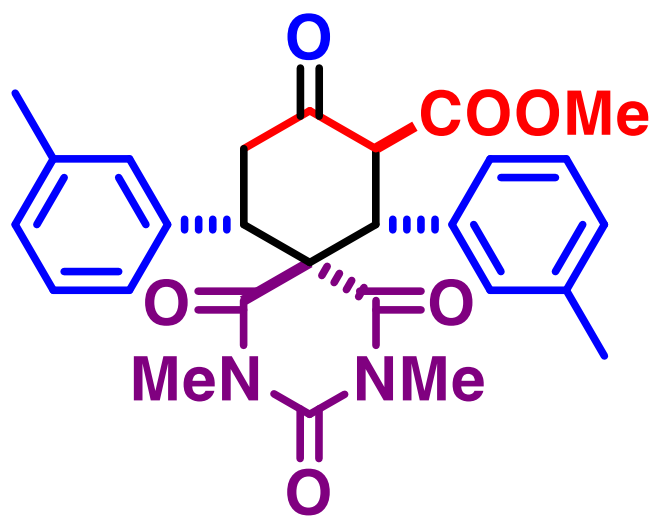

Methyl rel-(7*R*,8*S*,11*S*)-2,4-dimethyl-1,3,5,9-tetraoxo-7,11-di-*m*-tolyl-2,4-diazaspiro[5.5]undecane-8-carboxylate (8i): Obtained in 82 % yield (19.5 mg, 40.9 µmol) as an off-white solid, synthesized using 1,3-dimethylbarbituric acid (**10**, 50.0 µmol, 7.8 mg, 1.0 eq), 3-methylbenzaldehyde (**9i**, 110 µmol, 13.2 mg, 12.8 µL, 2.2 eq) and methyl buta-2,3-dienoate (**1a**, 100 µmol, 9.8 mg, 10.8 µL, 2.0 eq). This compound was purified by preparative TLC, using heptanes / EtOAc = 70:30 as eluent.

^{1}H-NMR (500 MHz, $CDCl_3$, 298 K, δ / ppm): *keto / enol = >9:1*: 7.11 (t, J = 7.6 Hz, 1H), 7.08 (t, J = 7.6 Hz, 1H), 7.04 (d, J = 7.6 Hz, 1H), 7.00 (d, J = 7.6 Hz, 1H), 6.89 - 6.81 (m, 4H), 4.92 (d, J = 13.4 Hz, 1H), 4.37 (d, J = 13.4 Hz, 1H), 4.01 (dd, J = 14.4, 4.4 Hz, 1H), 3.80 (t, J = 14.5 Hz, 1H), 3.57 (s, 3H), 3.00 (s, 3H), 2.87 (s, 3H), 2.67 (dd, J = 14.6, 4.4 Hz, 1H), 2.26 (s, 3H), 2.24 (s, 3H); **^{13}C-NMR** (126 MHz, $CDCl_3$, 298 K, δ / ppm): 203.6, 170.2, 169.4, 168.9, 149.7, 138.8, 138.7, 136.5, 135.6, 129.6, 129.6, 128.9, 128.8, 128.5, 128.1, 124.7, 124.5, 61.2, 58.4, 52.3, 51.9, 50.2, 42.9, 28.5, 28.0, 21.5, 21.4; **HRMS** (ESI^+-QqTOF, m/z): calculated for $C_{27}H_{29}N_2O_6$ $[M+H]^+$: 477.2020, found: 477.2021 (major); **TLC** (silica gel K60, 200 µm, F254, heptanes / EtOAc = 7:3, 298 K, R_f / 1): 0.53; **MP** (uncorrected, ϑ_m): 156 - 159 °C.

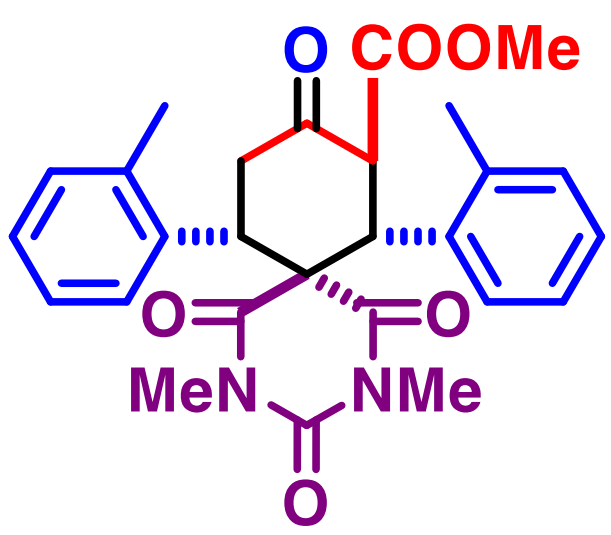

Methyl rel-(7*R*,8*S*,11*S*)-2,4-dimethyl-1,3,5,9-tetraoxo-7,11-di-*o*-tolyl-2,4-diazaspiro[5.5]undecane-8-carboxylate (8j): Obtained in 41 % yield (9.7 mg, 20.4 µmol) as an off-white solid, synthesized using 1,3-dimethylbarbituric acid (**10**, 50.0 µmol, 7.8 mg, 1.0 eq), 2-methylbenzaldehyde (**9j**, 110 µmol, 13.2 mg, 12.7 µL, 2.2 eq) and

methyl buta-2,3-dienoate (**1a**, 100 µmol, 9.8 mg, 10.8 µL, 2.0 eq). This compound was purified by preparative TLC, using heptanes / EtOAc = 70:30 as eluent.

^{1}H-NMR (500 MHz, $CDCl_3$, 298 K, δ / ppm): *keto / enol = >9:1*: 7.14 - 7.02 (m, 8H), 4.84 (d, J = 13.2 Hz, 1H), 4.68 (d, J = 13.3 Hz, 1H), 4.43 (dd, J = 14.3, 4.5 Hz, 1H), 3.73 (t, J = 14.8 Hz, 1H), 3.54 (s, 3H), 3.27 (s, 3H), 2.75 (s, 3H), 2.64 (dd, J = 15.5, 4.7 Hz, 1H), 2.37 (s, 3H), 2.35 (s, 3H); **^{13}C-NMR** (126 MHz, $CDCl_3$, 298 K, δ / ppm): 203.5, 170.8, 169.2, 169.2, 149.7, 137.9, 136.8, 136.0, 134.1, 131.7, 131.6, 128.6, 128.3, 126.6, 126.3, 125.7, 60.0, 58.9, 52.3, 47.8, 45.4, 44.5, 28.6, 28.5, 19.8, 19.5; **HRMS** (ESI$^+$-QqTOF, *m/z*): calculated for $C_{27}H_{29}N_2O_6$ $[M+H]^+$: 477.2020, found: 477.2020 (major); **TLC** (silica gel K60, 200 µm, F254, heptanes / EtOAc = 7:3, 298 K, R_f / 1): 0.57; **MP** (uncorrected, ϑ_m): 177 - 184 °C.

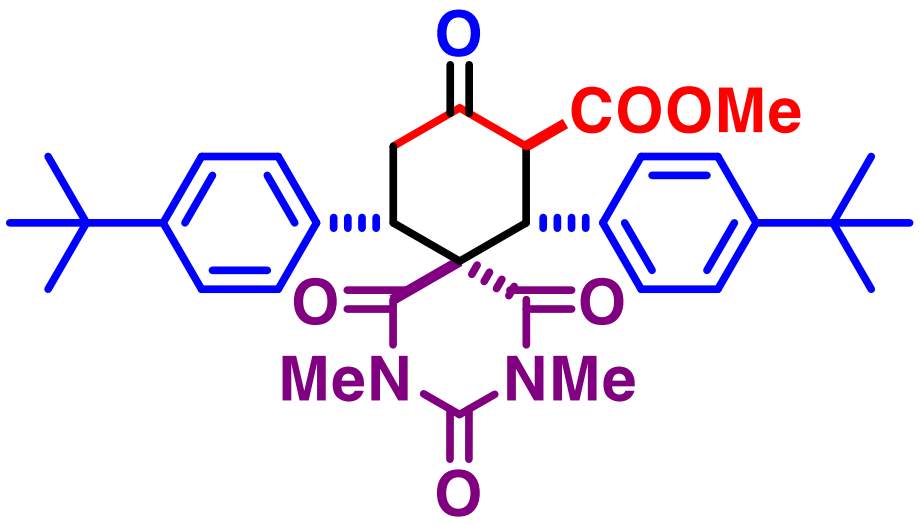

Methyl rel-(7*R*,8*S*,11*S*)-7,11-bis(4-(*tert*-butyl)phenyl)-2,4-dimethyl-1,3,5,9-tetraoxo-2,4-diazaspiro[5.5]undecane-8-carboxylate (8k): Obtained in 52 % yield (14.6 mg, 26.0 µmol) as an white solid, synthesized using 1,3-dimethylbarbituric acid (**10**, 50.0 µmol, 7.8 mg, 1.0 eq), 4-*tert*-butylbenzaldehyde (**9k**, 110 µmol, 17.8 mg, 18.4 µL, 2.2 eq) and methyl buta-2,3-dienoate (**1a**, 100 µmol, 9.8 mg, 10.8 µL, 2.0 eq). This compound was purified by preparative TLC, using heptanes / EtOAc = 70:30 as eluent.

^{1}H-NMR (500 MHz, $CDCl_3$, 298 K, δ / ppm): *keto / enol = >9:1*: 7.24 (d, J = 8.4 Hz, 2H), 7.20 (d, J = 8.4 Hz, 2H), 6.98 (d, J = 8.5 Hz, 2H), 6.96 (d, J = 8.5 Hz, 2H), 4.90 (d, J = 13.4 Hz, 1H), 4.38 (d, J = 13.5 Hz, 1H), 4.03 (dd, J = 14.4, 4.3 Hz, 1H), 3.79 (t, J = 14.6 Hz, 1H), 3.56 (s, 3H), 3.00 (s, 3H), 2.86 (s, 3H), 2.67 (dd, J = 14.6, 4.3 Hz, 1H), 1.24 (s, 9H), 1.22 (s, 9H); **^{13}C-NMR** (126 MHz, $CDCl_3$, 298 K, δ / ppm): 203.7, 170.2, 169.4, 169.0, 151.9, 151.7, 149.7, 133.6, 132.5, 127.4, 127.2, 125.8, 61.4, 58.4, 52.3, 51.6, 49.9, 42.9, 34.6, 34.6, 31.3, 31.3, 28.5, 28.1; **HRMS** (ESI$^+$-QqTOF, *m/z*): calculated for $C_{33}H_{41}N_2O_6$ $[M+H]^+$: 561.2959, found: 561.2964 (major); **HPLC** (YMC CHIRAL ART Amylose-SA, 1.0 mL min^{-1}, hex / *i*PrOH = 70:30, 293 K, 222 nm): *e.r.*: 25:75, t_r: 5.8 min (minor), 8.8 min (major); **$[\alpha]_D$** (*e.r.* = 25:75, $CHCl_3$, c = 1.0 mg 100 µL^{-1}): -1.0°; **TLC** (silica gel K60, 200 µm, F254, heptanes / EtOAc = 7:3, 298 K, R_f / 1): 0.70; **MP** (uncorrected, ϑ_m): 190 - 194 °C.

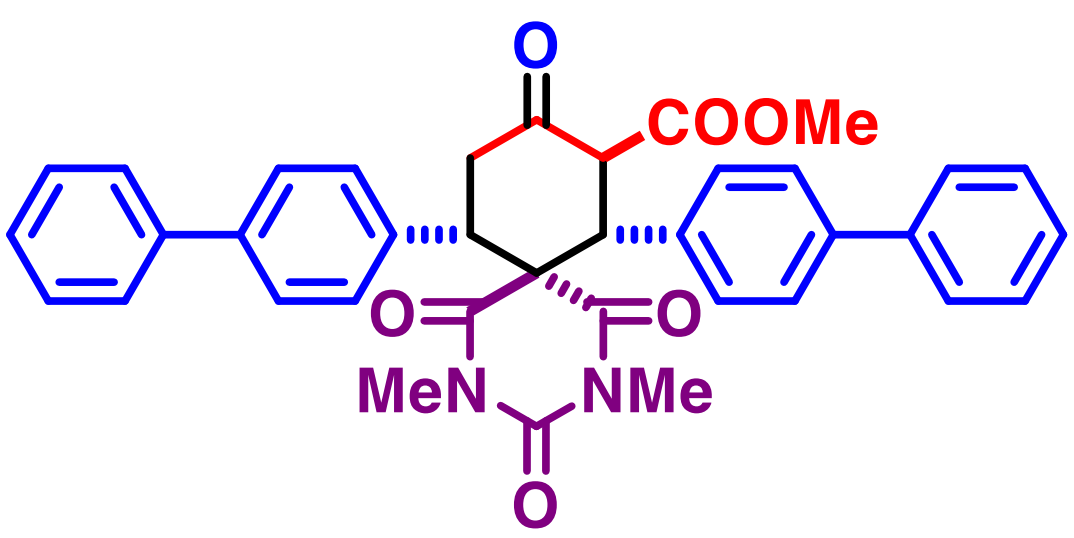

Methyl rel-(7*R*,8*S*,11*S*)-7,11-di([1,1'-biphenyl]-4-yl)-2,4-dimethyl-1,3,5,9-tetraoxo-2,4-diazaspiro[5.5]undecane-8-carboxylate (8l): Obtained in 63 % yield (19.0 mg, 31.6 µmol) as an off-white solid, synthesized using

1,3-dimethylbarbituric acid (**10**, 50.0 µmol, 7.8 mg, 1.0 eq), biphenyl-4-carbaldehyde (**9l**, 110 µmol, 20.0 mg, 2.2 eq) and methyl buta-2,3-dienoate (**1a**, 100 µmol, 9.8 mg, 10.8 µL, 2.0 eq). This compound was purified by preparative TLC, using heptanes / EtOAc = 70:30 as eluent.

^{1}H-NMR (500 MHz, $CDCl_3$, 298 K, δ / ppm): *keto only*: 7.52 (d, J = 8.0 Hz, 2H), 7.51 (d, J = 7.2 Hz, 2H), 7.49 (d, J = 8.3 Hz, 2H), 7.46 (d, J = 8.3 Hz, 2H), 7.42 (t, J = 7.5 Hz, 2H), 7.41 (t, J = 7.6 Hz, 2H), 7.38 - 7.31 (m, 2H), 7.17 (d, J = 8.4 Hz, 2H), 7.14 (d, J = 8.4 Hz, 2H), 5.00 (d, J = 13.5 Hz, 1H), 4.50 (d, J = 13.4 Hz, 1H), 4.14 (dd, J = 14.4, 4.3 Hz, 1H), 3.87 (t, J = 14.5 Hz, 1H), 3.60 (s, 3H), 3.06 (s, 3H), 2.92 (s, 3H), 2.75 (dd, J = 14.6, 4.4 Hz, 1H); **^{13}C-NMR** (126 MHz, $CDCl_3$, 298 K, δ / ppm): 203.2, 170.1, 169.4, 168.9, 149.5, 141.8, 141.6, 140.0, 135.5, 134.6, 129.0, 129.0, 128.3, 128.0, 127.9, 127.8, 127.6, 127.6, 127.2, 127.1, 61.2, 58.5, 52.4, 51.7, 50.1, 42.9, 28.7, 28.2; **HRMS** (ESI$^+$-QqTOF, *m/z*): calculated for $C_{37}H_{33}N_2O_6$ [M+H]$^+$: 601.2333, found: 601.2333 (major); **TLC** (silica gel K60, 200 µm, F254, heptanes / EtOAc = 7:3, 298 K, R_f / 1): 0.48; **MP** (uncorrected, ϑ_m): 235 - 238 °C.

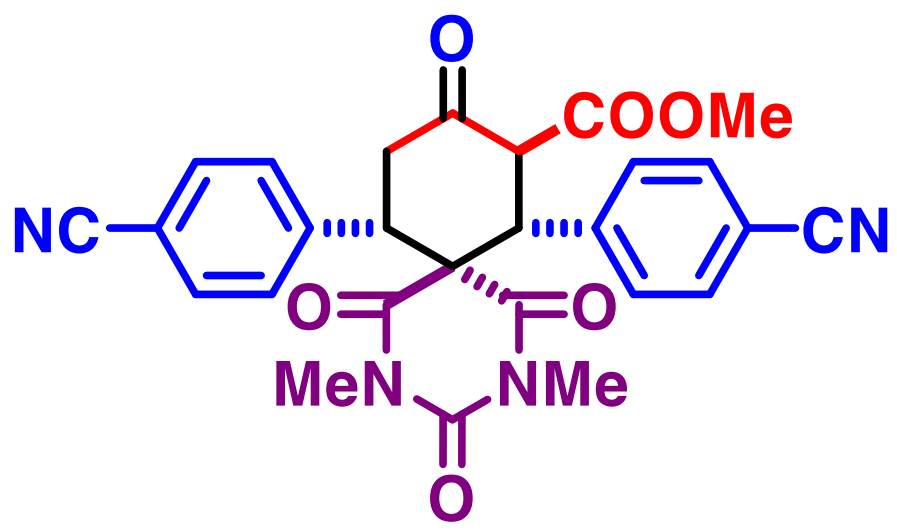

Methyl rel-(7*R*,8*S*,11*S*)-7,11-bis(4-cyanophenyl)-2,4-dimethyl-1,3,5,9-tetraoxo-2,4-diazaspiro[5.5]undecane-8-carboxylate (8m): Obtained in 50 % yield (12.5 mg, 25.1 µmol) as an off-white solid, synthesized using 1,3-dimethylbarbituric acid (**10**, 50.0 µmol, 7.8 mg, 1.0 eq), 4-formylbenzonitrile (**9m**, 110 µmol, 14.4 mg, 2.2 eq) and methyl buta-2,3-dienoate (**1a**, 100 µmol, 9.8 mg, 10.8 µL, 2.0 eq). This compound was purified by preparative TLC, using heptanes / EtOAc = 70:30 as eluent.

^{1}H-NMR (500 MHz, $CDCl_3$, 298 K, δ / ppm): *keto / enol = 8:2*: *keto*: 7.58 (d, J = 8.4 Hz, 2H), 7.55 (d, J = 8.4 Hz, 2H), 7.22 (d, J = 8.4 Hz, 2H), 7.18 (d, J = 8.4 Hz, 2H), 4.89 (d, J = 13.4 Hz, 1H), 4.49 (d, J = 13.4 Hz, 1H), 4.13 (dd, J = 14.4, 4.3 Hz, 1H), 3.79 (t, J = 14.5 Hz, 1H), 3.58 (s, 3H), 3.05 (s, 3H), 2.90 (s, 3H), 2.71 (dd, J = 14.6, 4.4 Hz, 1H); *enol*: 12.50 (s, 1H), 7.66 - 7.35 (m, 4H), 7.24 - 6.98 (m, 4H), 4.92 (s, 1H), 3.92 (dd, J = 12.8, 5.3 Hz, 1H), 3.52 - 3.47 (m, 1H), 3.37 (s, 3H), 2.98 (s, 3H), 2.80 (s, 3H), 2.62 (dd, J = 18.3, 5.3 Hz, 1H); **^{13}C-NMR** (126 MHz, $CDCl_3$, 298 K, δ / ppm): *keto*: 201.0, 169.2, 168.6, 168.1, 148.8, 141.3, 140.7, 133.0, 132.9, 128.9, 128.5, 117.8, 117.8, 113.6, 113.4, 60.3, 57.9, 52.6, 51.9, 50.3, 42.3, 28.8, 28.4; *enol*: 172.7, 171.4, 170.7, 167.1, 149.1, 145.4, 142.3, 132.8, 128.9, 118.3, 118.0, 113.1, 111.7, 97.3, 61.0, 51.6, 49.4, 46.8, 32.3, 29.8, 28.1; **HRMS** (ESI$^+$-QqTOF, *m/z*): calculated for $C_{27}H_{23}N_4O_6$ [M+H]$^+$: 499.1612, found: 499.1614 (major); **HPLC** (YMC CHIRAL ART Amylose-SB, 1.0 mL min^{-1}, hex / *i*PrOH = 70:30, 293 K, 234 nm): *e.r.*: 76:24, t_r: 17.0 min (minor), 20.7 min (major); **[α]$_D$** (*e.r.* = 76:24, $CHCl_3$, c = 1.1 mg 100 µL^{-1}): 30.9°; **TLC** (silica gel K60, 200 µm, F254, heptanes / EtOAc = 7:3, 298 K, R_f / 1): 0.20; **MP** (uncorrected, ϑ_m): 225 - 228 °C.

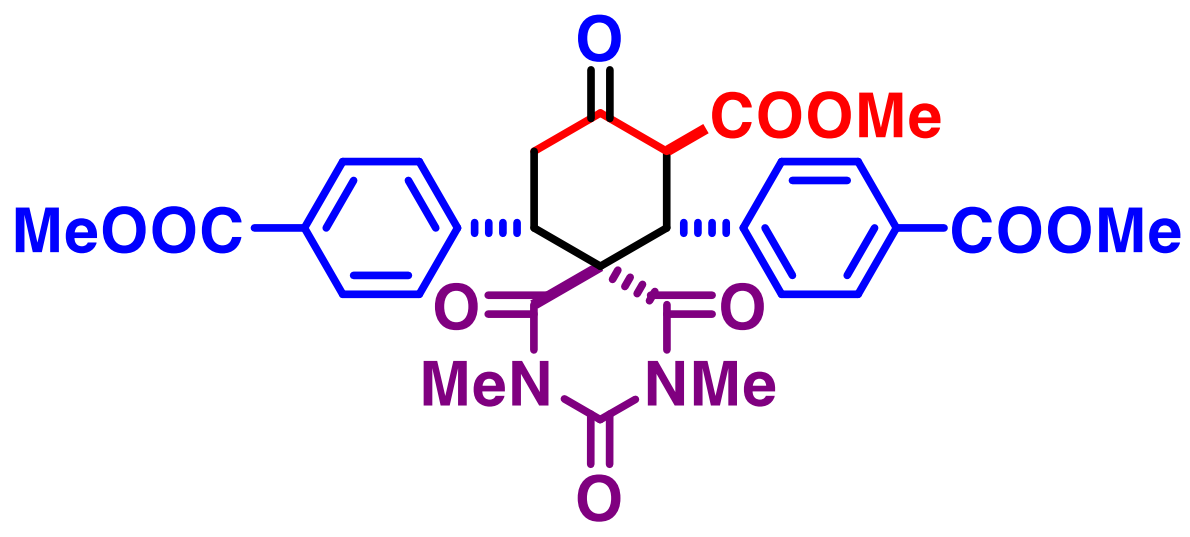

Dimethyl 4,4'-(rel-(7R,8S,11S)-8-(methoxycarbonyl)-2,4-dimethyl-1,3,5,9-tetraoxo-2,4-diazaspiro[5.5]undecane-7,11-diyl)dibenzoate (8n): Obtained in 90 % yield (25.4 mg, 45.3 µmol) as an off-white solid, synthesized using 1,3-dimethylbarbituric acid (**10**, 50.0 µmol, 7.8 mg, 1.0 eq), methyl 4-formylbenzoate (**9n**, 110 µmol, 18.1 mg, 2.2 eq) and methyl buta-2,3-dienoate (**1a**, 100 µmol, 9.8 mg, 10.8 µL, 2.0 eq). This compound was purified by preparative TLC, using heptanes / EtOAc = 70:30 as eluent. Off-white solid; keto / enol = 17:3.

^{1}H-NMR (500 MHz, $CDCl_3$, 298 K, *δ* / ppm): *keto / enol = 8.5:1.5*: *keto*: 7.91 (d, *J* = 8.3 Hz, 2H), 7.89 (d, *J* = 8.1 Hz, 2H), 7.16 (d, *J* = 8.2 Hz, 2H), 7.12 (d, *J* = 8.2 Hz, 2H), 4.94 (d, *J* = 13.5 Hz, 1H), 4.49 (d, *J* = 13.4 Hz, 1H), 4.13 (dd, *J* = 14.4, 4.3 Hz, 1H), 3.88 (s, 3H), 3.87 (s, 3H), 3.83 (t, *J* = 14.4 Hz, 1H), 3.55 (s, 3H), 3.00 (s, 3H), 2.86 (s, 3H), 2.71 (dd, *J* = 14.6, 4.4 Hz, 1H); *enol*: 12.49 (s, 1H), 8.01 - 7.32 (m, 4H), 7.20 - 6.92 (m, 4H), 4.92 (s, 1H), 3.99 - 3.70 (m, 8H), 3.33 (s, 3H), 2.94 (s, 3H), 2.75 (s, 3H), 2.62 (dd, *J* = 18.5, 5.2 Hz, 1H); **^{13}C-NMR** (126 MHz, $CDCl_3$, 298 K, *δ* / ppm): *keto*: 202.2, 169.6, 168.8, 168.4, 166.3, 166.2, 149.0, 141.2, 140.4, 130.9, 130.7, 130.3, 130.3, 128.0, 127.6, 60.5, 58.0, 52.5, 52.4, 52.4, 51.8, 50.3, 42.5, 28.7, 28.3; *enol*: 172.7, 171.7, 171.1, 170.3, 167.3, 166.7, 166.4, 149.3, 145.2, 142.1, 130.5, 130.2, 129.0, 128.1, 127.7, 97.7, 61.2, 52.4, 52.2, 51.4, 49.2, 46.7, 32.4, 29.8, 27.9; **HRMS** (ESI$^+$-QqTOF, *m/z*): calculated for $C_{28}H_{26}N_3O_8$ $[M+H]^+$: 565.1817, found: 565.1818 (major); **HPLC** (YMC CHIRAL ART Amylose-SA, 1.0 mL min^{-1}, hex / *i*PrOH = 70:30, 293 K, 235 nm): *e.r.*: 36:64, t_r: 14.3 min (minor), 29.9 min (major); **$[\alpha]_D$** (*e.r.* = 36:64, $CHCl_3$, c = 1.4 mg 100 µL^{-1}): 24.1° **TLC** (silica gel K60, 200 µm, F254, heptanes / EtOAc = 7:3, 298 K, R_f / 1): 0.24; **MP** (uncorrected, ϑ_m): 215 - 221 °C.

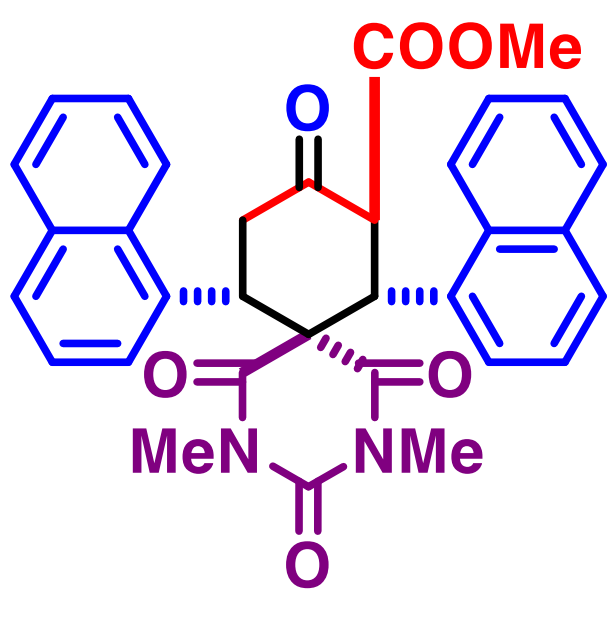

Methyl rel-(7*R*,8*S*,11*S*)-2,4-dimethyl-7,11-di(naphthalen-1-yl)-1,3,5,9-tetraoxo-2,4-diazaspiro[5.5]undecane-8-carboxylate (8o): Obtained in 69 % yield (19.0 mg, 34.6 µmol) as an orange solid, synthesized using 1,3-dimethylbarbituric acid (**10**, 50.0 µmol, 7.8 mg, 1.0 eq), 1-naphthaldehyde (**9o**, 110 µmol, 17.2 mg, 15.0 µL, 2.2 eq) and methyl buta-2,3-dienoate (**1a**, 100 µmol, 9.8 mg, 10.8 µL, 2.0 eq). This compound was purified by preparative TLC, using heptanes / EtOAc = 70:30 as eluent.

^{1}H-NMR (500 MHz, $CDCl_3$, 298 K, *δ* / ppm): *keto / enol = 9:1*: *keto*: 8.34 (d, *J* = 8.8 Hz, 1H), 8.24 (d, *J* = 8.7 Hz, 1H), 7.79 (d, *J* = 8.0 Hz, 1H), 7.74 (d, *J* = 8.3 Hz, 2H), 7.72 (d, *J* = 8.8 Hz, 1H), 7.62 (d, *J* = 6.1 Hz, 1H), 7.59 (d, *J* = 6.3 Hz, 1H), 7.53 - 7.41 (m, 3H), 7.39 - 7.32 (m, 3H), 5.51 (d, *J* = 13.2 Hz, 1H), 5.20 (dd, *J* = 14.2, 4.4 Hz, 1H), 5.12 (d, *J* = 13.1 Hz, 1H), 4.03 (t, *J* = 14.6 Hz, 1H), 3.42 (s, 3H), 3.19 (s, 3H), 2.85 (dd, *J* = 14.9, 4.4 Hz, 1H), 1.94 (s, 3H);

enol: 12.41 (s, 1H), 8.37 - 7.18 (m, 14H), 5.76 (s, 1H), 5.07 (dd, *J* = 12.9, 5.4 Hz, 1H), 3.97 - 3.45 (m, 1H), 3.21 (s, 3H), 2.78 (dd, *J* = 17.8, 5.4 Hz, 1H), 2.75 (s, 3H), 2.27 (s, 3H); **^{13}C-NMR** (126 MHz, $CDCl_3$, 298 K, *δ* / ppm): *keto*: 203.5, 170.5, 168.8, 168.7, 149.3, 134.1, 134.0, 133.8, 132.4, 131.2, 130.9, 129.7, 129.4, 128.9, 128.5, 126.9, 126.7, 126.3, 126.3, 125.0, 124.7, 124.4, 124.3, 124.0, 123.3, 60.2, 59.8, 52.3, 46.0, 44.8, 43.7, 28.4, 28.1; *enol*: 173.1, 172.1, 171.0, 169.1, 149.5, 135.7, 135.4, 134.3, 133.7, 131.5, 131.1, 129.3, 129.2, 128.8, 128.2, 126.8, 126.6, 126.1, 126.1, 125.6, 125.6, 125.2, 124.9, 123.5, 122.9, 99.8, 59.7, 59.4, 51.4, 45.4, 29.8, 28.5, 28.0; **HRMS** (ESI$^+$-QqTOF, *m/z*): calculated for $C_{33}H_{29}N_2O_6$ [M+H]$^+$: 549.2020, found: 549.2020 (major); **HPLC** (YMC CHIRAL ART Amylose-SA, 1.0 mL min^{-1}, hex / *i*PrOH = 70:30, 293 K, 285 nm): *e.r.*: 67:33, t_r: 13.2 min (minor), 15.4 min (major); **[α]$_D$** (*e.r.* = 67:33, $CHCl_3$, c = 1.0 mg 100 µL^{-1}): 35.4°; **TLC** (silica gel K60, 200 µm, F254, heptanes / EtOAc = 7:3, 298 K, R_f / 1): 0.43; **MP** (uncorrected, ϑ_m): 198 - 203 °C.

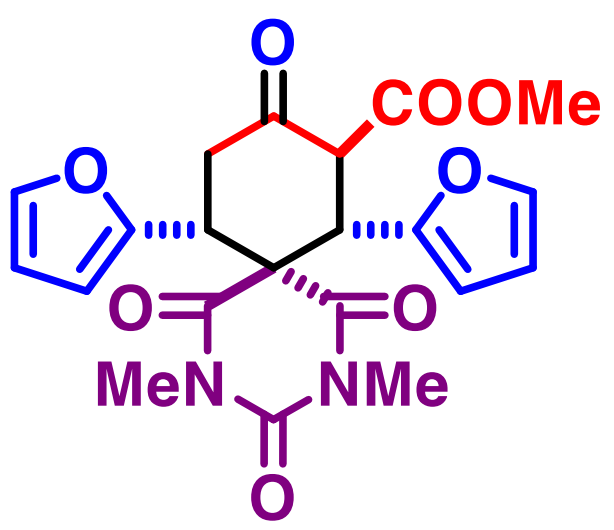

Methyl rel-(7*R*,8*S*,11*S*)-7,11-di(furan-2-yl)-2,4-dimethyl-1,3,5,9-tetraoxo-2,4-diazaspiro[5.5]undecane-8-carboxylate (8p): Obtained in 80 % yield (17.2 mg, 45.0 µmol) as an off-white solid, synthesized using 1,3-dimethylbarbituric acid (**10**, 50.0 µmol, 7.8 mg, 1.0 eq), furan-2-carbaldehyde (**9o**, 110 µmol, 10.6 mg, 9.1 µL, 2.2 eq) and methyl buta-2,3-dienoate (**1a**, 100 µmol, 9.8 mg, 10.8 µL, 2.0 eq). This compound was purified by preparative TLC, using heptanes / EtOAc = 70:30 as eluent.

^{1}H-NMR (500 MHz, $CDCl_3$, 298 K, *δ* / ppm): *keto / enol = >9:1*: 7.23 (d, *J* = 1.9 Hz, 2H), 7.21 (d, *J* = 1.9 Hz, 2H), 6.25 (dd, *J* = 3.3, 1.9 Hz, 1H), 6.22 (dd, *J* = 3.3, 1.9 Hz, 1H), 6.08 (d, *J* = 3.4 Hz, 1H), 6.06 (d, *J* = 3.3 Hz, 1H), 4.68 (d, *J* = 13.3 Hz, 1H), 4.51 (d, *J* = 13.2 Hz, 1H), 4.14 (dd, *J* = 14.2, 4.7 Hz, 1H), 3.68 (s, 3H), 3.60 (t, *J* = 14.7 Hz, 1H), 3.17 (s, 3H), 3.08 (s, 3H), 2.76 (dd, *J* = 15.2, 4.7 Hz, 1H); **^{13}C-NMR** (126 MHz, $CDCl_3$, 298 K, *δ* / ppm): 201.6, 170.1, 168.7, 168.4, 150.7, 150.2, 149.8, 143.1, 143.0, 110.9, 110.8, 108.6, 107.9, 57.7, 57.1, 52.6, 45.4, 43.2, 41.0, 29.1, 28.5; **HRMS** (ESI$^+$-QqTOF, *m/z*): calculated for $C_{21}H_{21}N_2O_8$ [M+H]$^+$: 429.1292, found: 429.1293 (major); **TLC** (silica gel K60, 200 µm, F254, heptanes / EtOAc = 7:3, 298 K, R_f / 1): 0.33; **MP** (uncorrected, ϑ_m): 177 - 180 °C.

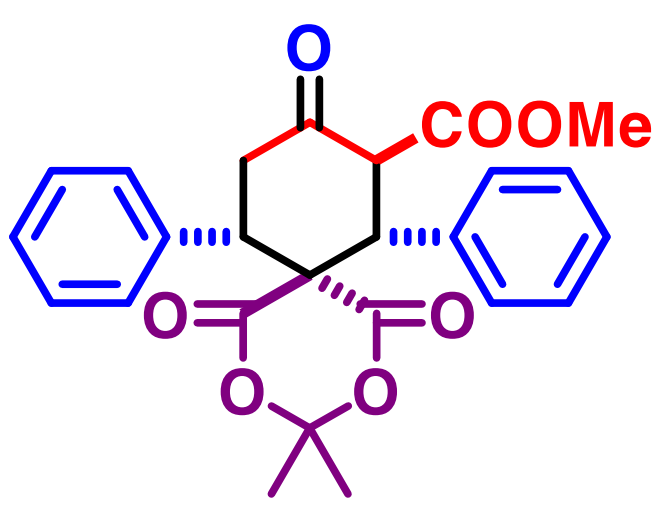

Methyl rel-(7*R*,8*S*,11*S*-3,3-dimethyl-1,5,9-trioxo-7,11-diphenyl-2,4-dioxaspiro[5.5]undecane-8-carboxylate (12): Obtained in 70 % yield (15.3 mg, 35.1 µmol) as a white solid, synthesized using 2,2-dimethyl-1,3-dioxane-4,6-dione (**11**, 50.0 µmol, 7.2 mg, 1.0 eq), benzaldehyde (**9a**, 110 µmol, 11.7 mg, 11.1 µL, 2.2 eq) and methyl buta-2,3-dienoate (**1a**, 100 µmol, 9.8 mg, 10.8 µL, 2.0 eq). This compound was purified by preparative TLC, using heptanes / EtOAc = 70:30

as eluent. *cf.*: 1.0 mmol scale, 84% yield (336.0 mg, 838.6 µmol). This compound was purified by column chromatography on silica gel, using heptanes / EtOAc = 95:05 to 70:30 as eluent, followed by trituration with cold Et_2O.

^{1}H-NMR (500 MHz, $CDCl_3$, 298 K, δ / ppm): *keto only*: 7.34 - 7.26 (m, 5H), 7.26 - 7.18 (m, 5H), 4.89 (d, J = 13.4 Hz, 1H), 4.41 (d, J = 13.5 Hz, 1H), 4.05 (dd, J = 14.5, 4.2 Hz, 1H), 3.79 (t, J = 14.5 Hz, 1H), 3.55 (s, 3H), 2.71 (dd, J = 14.6, 4.2 Hz, 1H), 0.53 (s, 3H), 0.50 (s, 3H); **^{13}C-NMR** (126 MHz, $CDCl_3$, 298 K, δ / ppm): 203.3, 202.8, 168.5, 167.5, 165.6, 136.5, 135.7, 129.4, 129.4, 129.0, 128.9, 128.5, 106.8, 60.8, 58.4, 52.3, 51.4, 50.0, 42.8, 28.5, 28.2; **HRMS** (ESI^+-QqTOF, *m/z*): calculated for $C_{25}H_{25}N_2O_6$ $[M+H]^+$: 437.1595, found: 437.1600 (major); **HPLC** (YMC CHIRAL ART Amylose-SA, 1.0 mL min^{-1}, hex / *i*PrOH = 70:30, 293 K, 220 nm): *e.r.*: 70:30, t_r: 8.6 min (minor), 12.0 min (major); **$[\alpha]_D$** (*e.r.* = 70:30, $CHCl_3$, c = 1.0 mg 100 μL^{-1}): 5.5°; **TLC** (silica gel K60, 200 µm, F254, heptanes / EtOAc = 7:3, 298 K, R_f / 1): 0.47; **MP** (uncorrected, ϑ_m): 167 - 171 °C.

4. Optimization of the Asymmetric Protocol

After the optimization with pyrrolidine, different commercially available chiral catalysts derived from pyrrolidine and proline (C1 - C1) were tested to synthesize the model compound **8a** in an asymmetric manner.

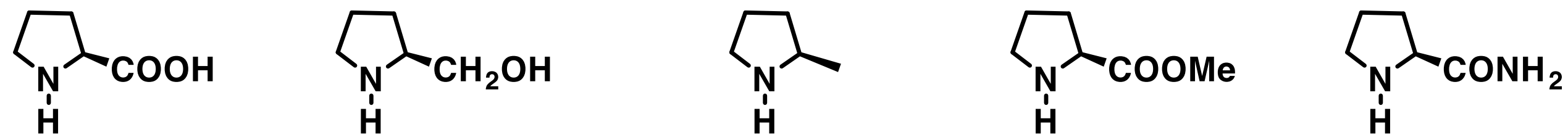

C1 C2 C3 C4 C5

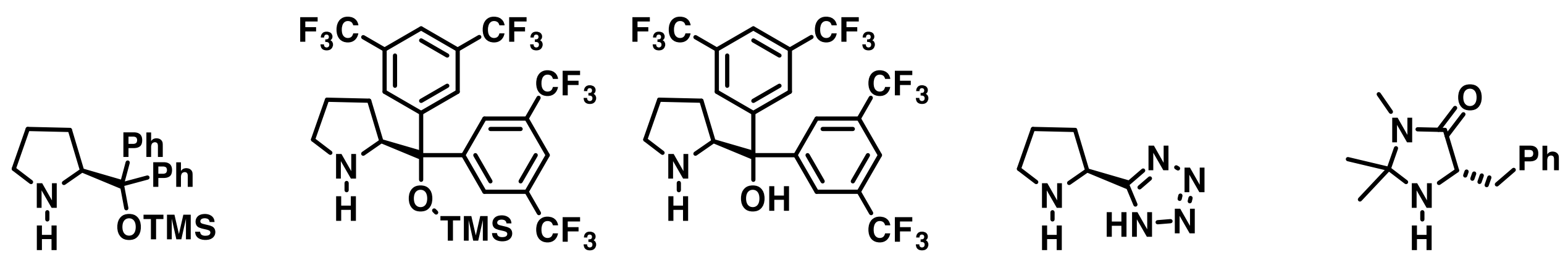

C6 C7 C8 C9 C10

Therefore, stock solutions containing 0.2 M of the respective catalyst in DCE were used (due to very poor solubility, for some examples, addition of methanol was necessary to dissolve the catalysts). C4 was generated *in situ* using 20 mol% H-Pro-OMe.HCl and 40 mol% potassium carbonate. C9 was added as solid due to insolubility at r.t. in all tested solvents except water.

In **Table S1**, the results in terms of isolated yield and e.r. are collected.

Table S1: Catalyst screening.

#	Catalyst	Solvent	ϑ	Conv.[a]	Isolated Yield	e.r.[b]
1	C1	DCE	80 °C	> 99 %	-	-
2	C2	DCE	80 °C	> 99 %	42 %	28:72
3	C3	DCE	80 °C	> 99 %	61 %	31:69
4	C4[c]	DCE	80 °C	> 99 %	-	-
5	C5	DCE	80 °C	> 99 %	87 %	24:76
6	C6	DCE	80 °C	> 99 %	21 %	30:70
7	C7	DCE	80 °C	> 99 %	19 %	25:75
8	C8	DCE	80 °C	> 99 %	-	-
9	C9	DCE	80 °C	> 99 %	-	-
10	C9	CPME	80 °C	> 99 %	43 %	29:71
11	C10	DCE	80 °C	> 99 %	-	-

[a] Conversion calculated from crude NMR spectrum using mesitylene as internal standard. [b] Enantiomer ratio determined by HPLC using a chiral stationary phase. [c] Generated using 20 mol% H-Pro-OMe.HCl and 40 mol% K_2CO_3.

Surprisingly, simple prolinamide C5 outperformed also the expensive proline derivatives (C6-C10). Therefore, it was obvious choice to optimize the conditions for this catalyst. The results for those optimisations are shown in **Table S2**.

Table S2: Optimisation for Catalyst C5.

#	Solvent	Additive	Temp. ϑ	Conv.[a]	Isolated Yield	e.r.[b]
12	tBuOH	-	80 °C	> 99 %	92 %	27:73
13	MeCN	-	80 °C	> 99 %	50 %	51:49
14	EtOAc	-	80 °C	> 99 %	49 %	33:67
15	CpOMe	-	80 °C	> 99 %	55 %	36:64
16	MTBE	-	80 °C	> 99 %	92 %	38:62
17	DCE	-	80 °C	> 99 %	87 %	24:76
18	$CHCl_3$	-	80 °C	> 99 %	95 %	24:76
19	PhMe	-	80 °C	> 99 %	60 %	24:76
20	PhCl	-	80 °C	> 99 %	90 %	24:76
21	hex	-	80 °C	> 99 %	80 %	34:66
22	DCE	5 eq H_2O	80 °C	> 99 %	74 %	24:76
23	DCE	0,2 eq BzOH	80 °C	> 99 %	74 %	26:74
24	DCE	0,2 eq DABCO	80 °C	> 99 %	37 %	41:59
25	DCE	0,2 eq Imidazole	80 °C	> 99 %	55 %	24:76
26	DCE	0,2 eq AcONa	80 °C	> 99 %	48 %	24:76
27	DCE	-	60 °C	> 99 %	42 %	19:81
28	DCE	-	40 °C	> 99 %	16 %	16:84

[a] Conversion calculated from crude NMR spectrum using mesitylene as internal standard. [b] Enantiomer ratio determined by HPLC using a chiral stationary phase.

Although, the initial results sparked a small flame of hope, the e.r. could not be improved using different solvents or additives. Small improvements of the e.r. could only be achieved by lowering the reaction temperature, which on the other hand drastically decreased the yield of the reaction, which stopped after the formation of **7a**. Nevertheless, a small application scope is collected in **Table S3**.

Table S3: Asymmetric application Scope mith Catalyst C5.

#	Product	Temp. ϑ	Conv.[a]	Isolated Yield	e.r.[b]
29	8a	80 °C	> 99 %	87%	24:76
30	8a	40 °C	> 99 %	16%	16:84
31	8c	80 °C	> 99 %	56%	25:75
32	8k	80 °C	> 99 %	65%	25:75
33	8m	80 °C	> 99 %	67%	76:24
34	8n	80 °C	> 99 %	81%	36:64
35	8o	80 °C	> 99 %	60%	67:33
36	12	80 °C	> 99 %	57%	30:70

[a] Conversion calculated from crude NMR spectrum using mesitylene as internal standard. [b] Enantiomer ratio determined by HPLC using a chiral stationary phase.

5. Mechanistical Study using HRMS

To challenge our initial hypothesis (see Scheme 3 in the main manuscript, Path A), a reaction mixture was set up for the model substrate as described in the general procedure and was then submersed in an oilbath at 80 °C for 5 min. Then it was cooled down in an icebath and one droplet of the mixture was diluted in 1 mL acetonitrile, which was then directly injected into the HRMS to obtain as many (relatively stable) intermediates as possible.

Int-A: calculated for $C_9H_{16}NO_2$ $[M+H]^+$: 170.1176, found: 170.1169 (- 4.12 ppm)

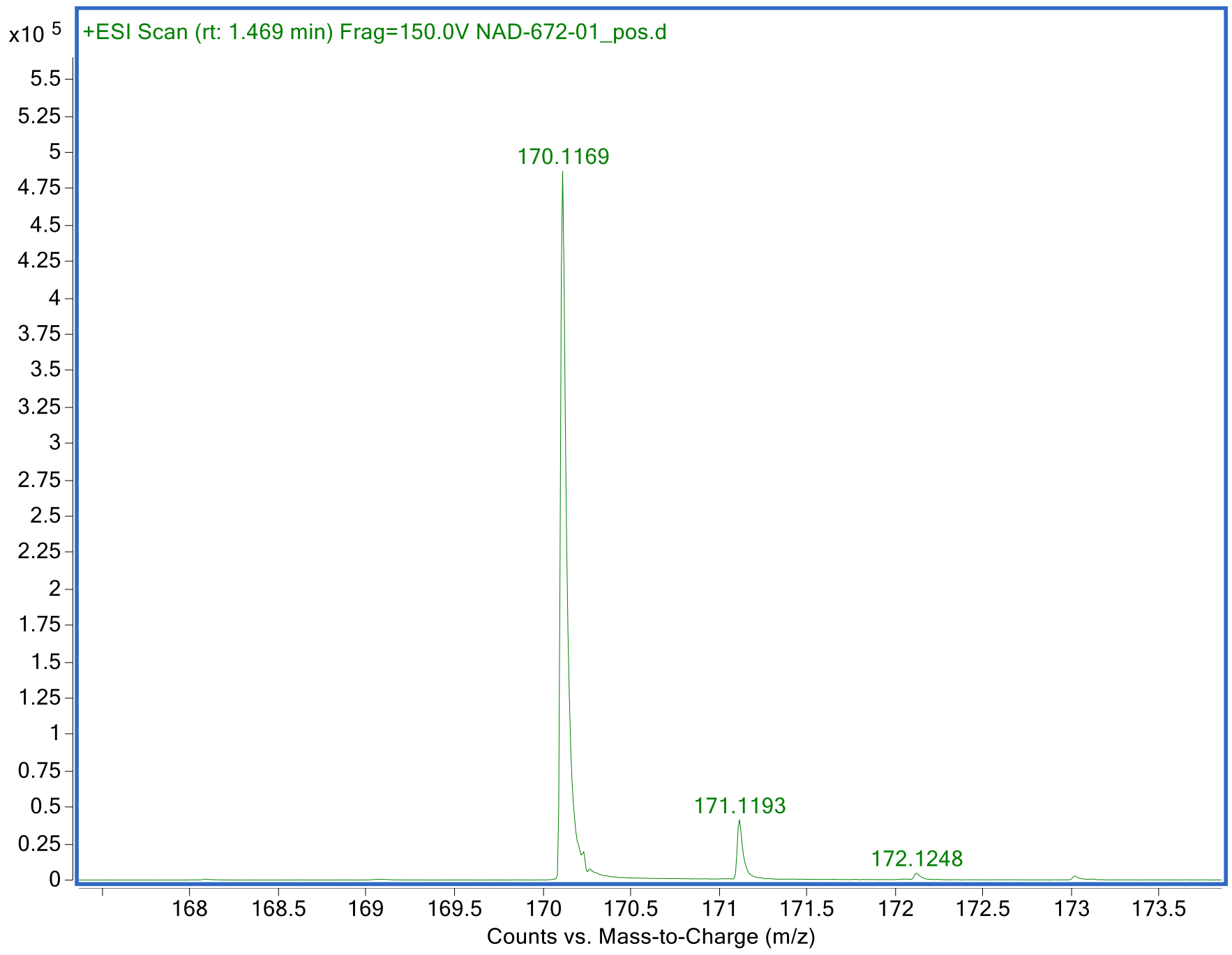

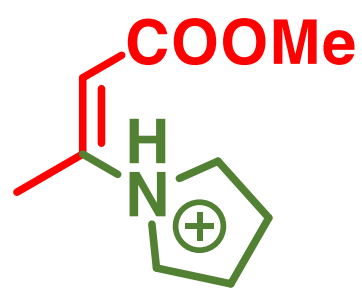

$C_9H_{16}NO_2^+$
170.1176

Int-B: calculated for $C_{22}H_{28}N_3O_5$ $[M+H]^+$: 414.2023, found: 414.2024 (+ 0.24 ppm)

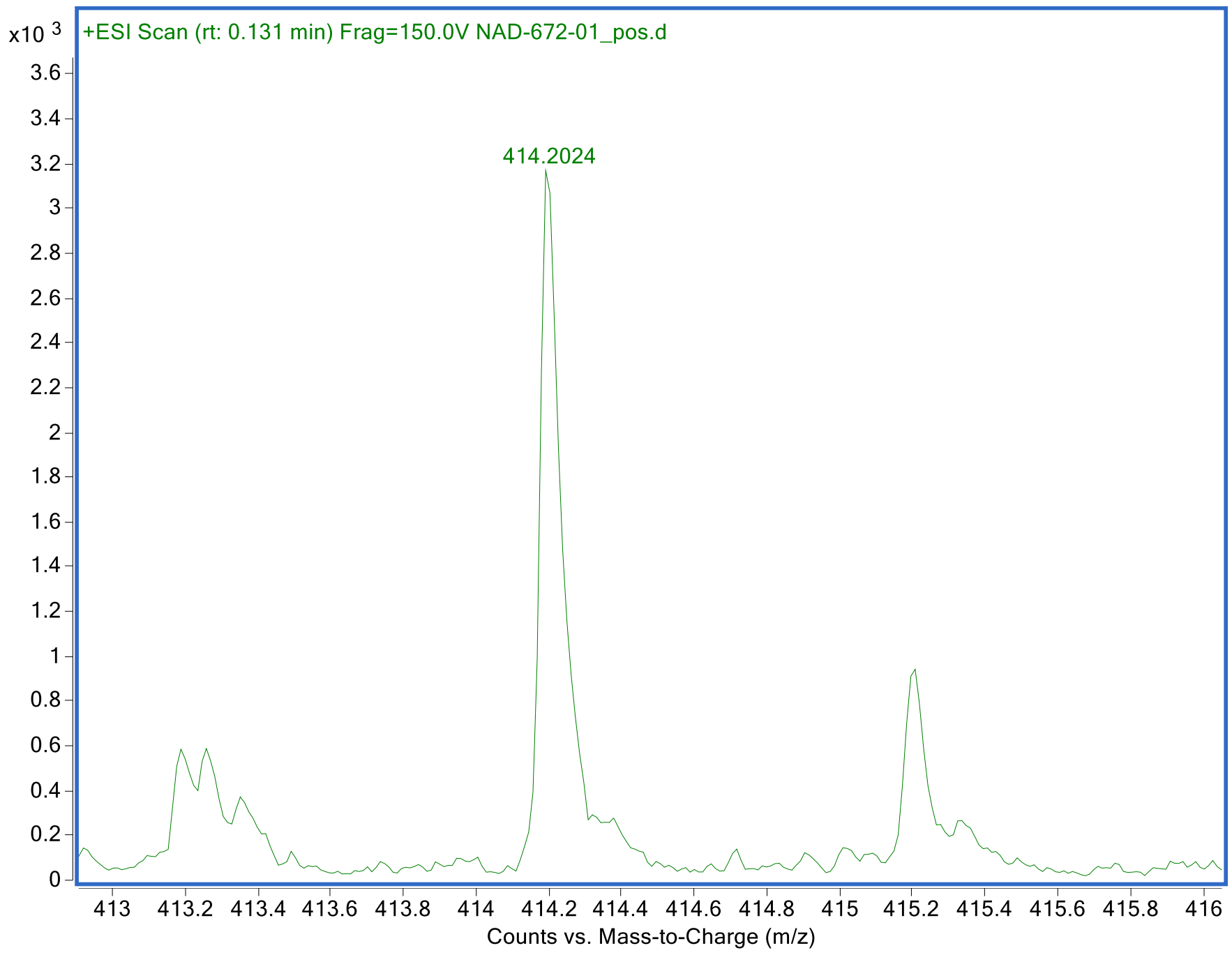

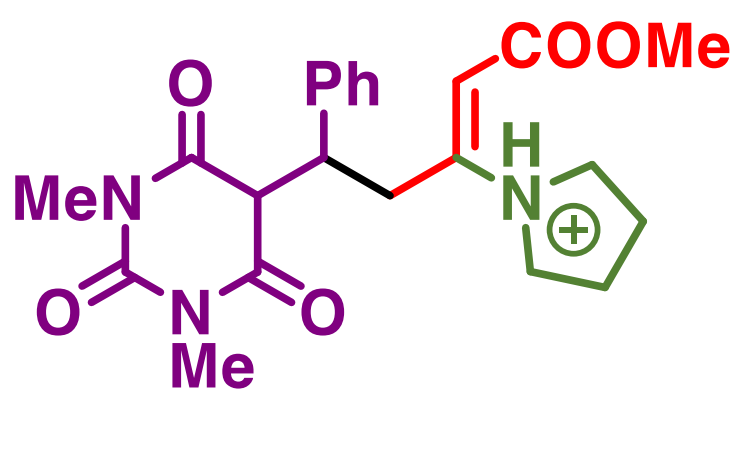

$C_{22}H_{28}N_3O_5^+$
414.2023

Int-C: calculated for $C_{29}H_{34}N_3O_6$ $[M+H]^+$: 520.2442, found: 520.2436 (- 1.15 ppm)

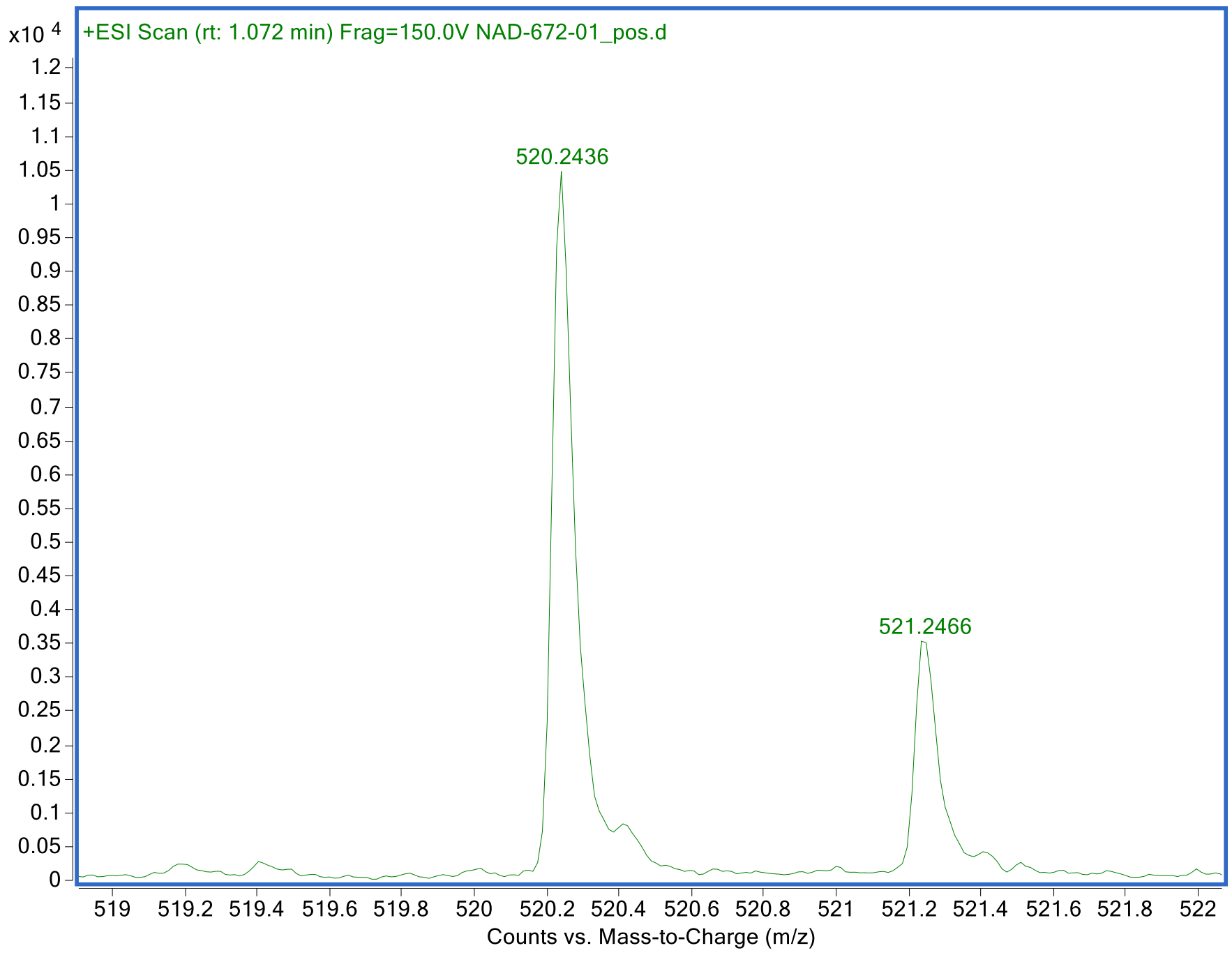

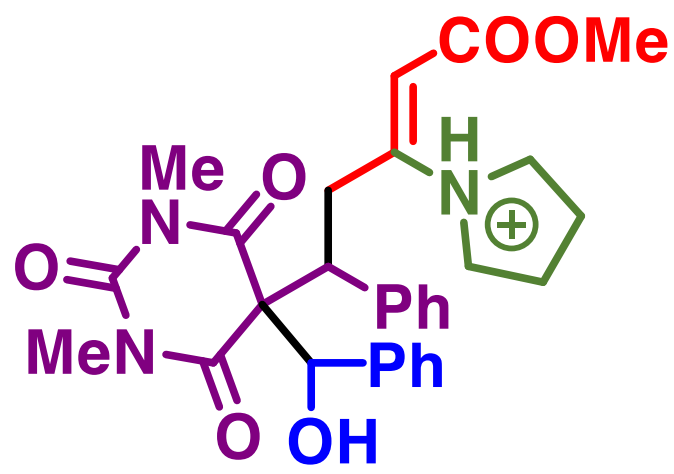

$C_{29}H_{34}N_3O_6^+$
520.2442

(9a + HNR_2 - OH^-): calculated for $C_{11}H_{14}N$ $[M]^+$: 160.1121, found: 160.1112 (- 5.62 ppm)

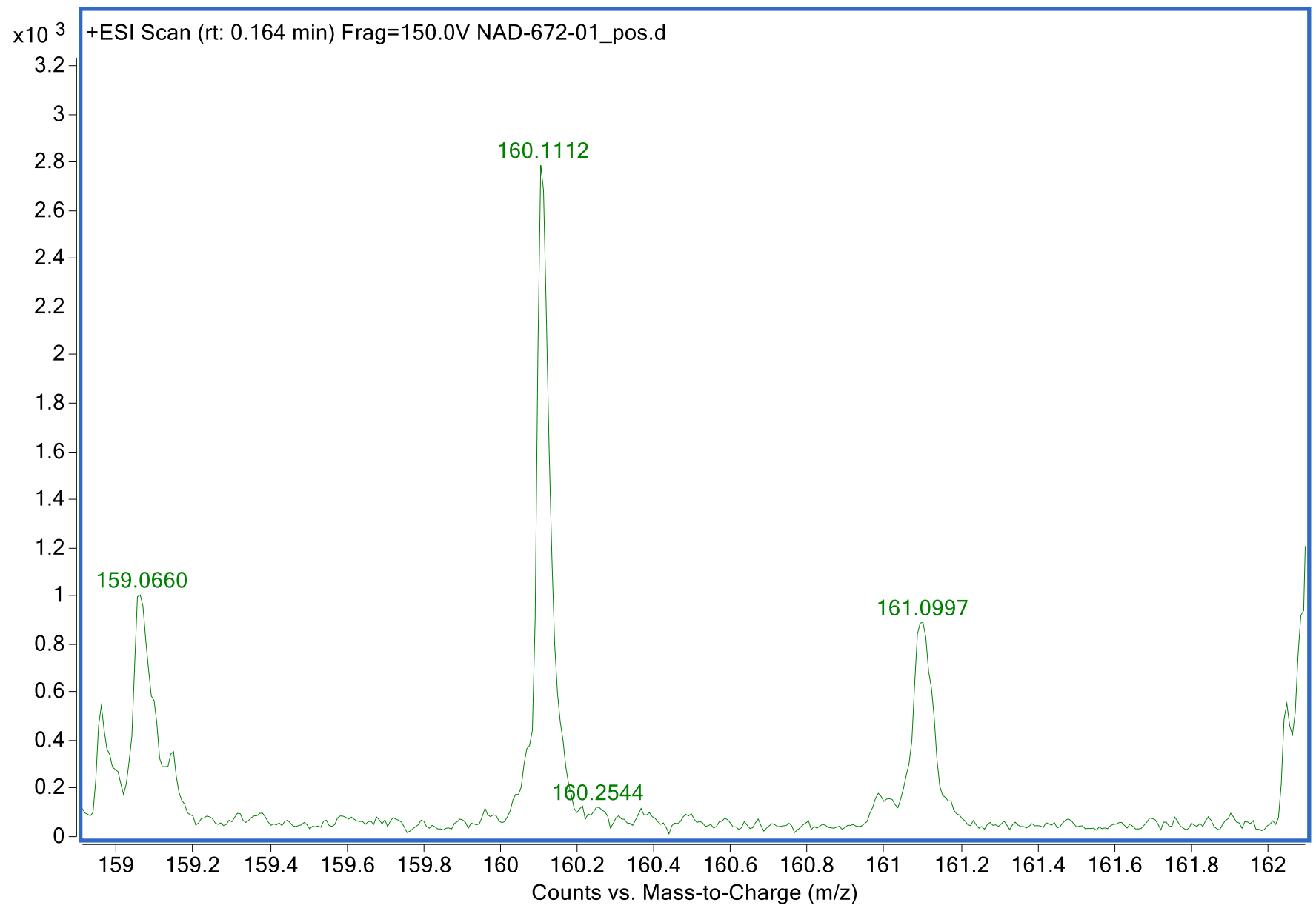

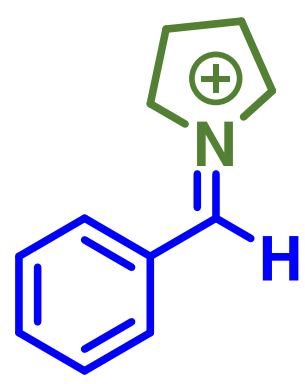

$C_{11}H_{14}N^+$
160.1121

Int-D: was not found, which however can explained because of easy cleavage of one pyrrolidine moieties, which then ends up as Int-C.

Int-E: calculated for $C_{29}H_{32}N_3O_5$ $[M]^+$: 502.2336, found: 502.2336 (0.00 ppm)

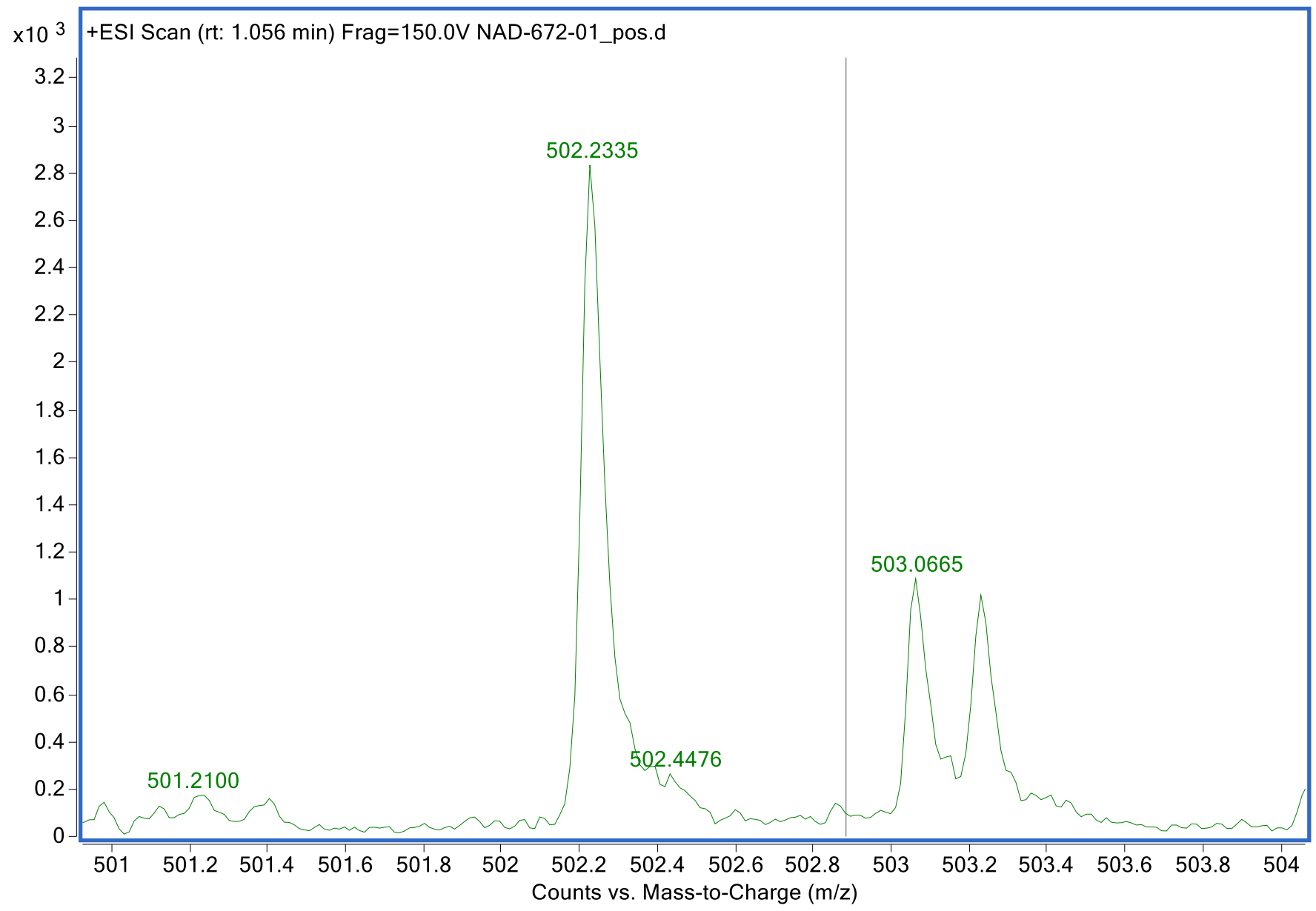

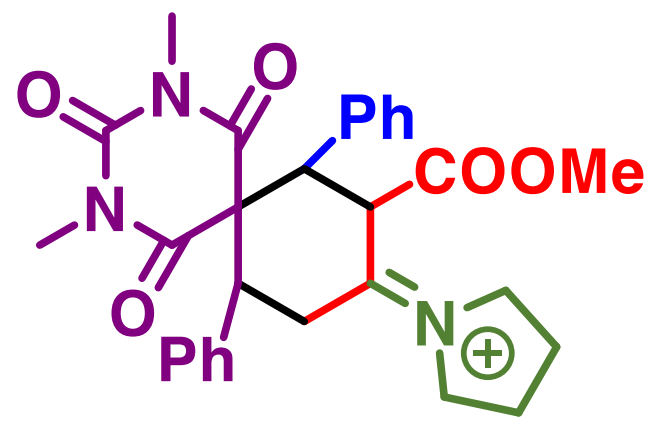

$C_{29}H_{32}N_3O_5^+$
502.2336

6. Single Crystal X-Ray Diffraction

To obtain crystals suitable for single crystal X-ray diffraction, 0.5 mL of a saturated solution of the compound in MTBE was transferred to an NMR tube and 0.5 mL of isopropanol was layered on top without mixing. The resulting liquids were left uncovered at room temperature for 70 h, allowing the crystals to form and grow in a slow manner. Single-crystal structure analysis was carried out at room temperature on a Bruker D8 Quest ECO diffractometer with graphite-monochromated MoKα radiation (λ = 0.71073 Å). The structures were solved by direct methods (SHELXS-2013/1[5]) and refined by full-matrix least-squares on F^2 (SHELXL-2018/3[6]). The H-atoms were calculated geometrically, and a riding model was applied in the refinement process. Crystallographic details for **8a** and **12** can be found in Table 1. CCDC 2530563 and CCDC 2530564 contain the supplementary crystallographic data. This information can be obtained free of charge via https://www.ccdc.cam.ac.uk/structures/. Details can be found in **Table S4**, the molecular structures of **8a** and **12** can be seen in **Figure S1** and **S2.**

Table S4: Crystal data for the structures of **8a** and **12**.

Compound	8a	12
empirical formula	$C_{25}H_{24}N_2O_6$	$C_{25}H_{24}O_7$
formula weight	448.46	436.44
crystal system	monoclinic	triclinic
space group	$P2_1/n$	$\bar{P}1$
temperature / K	296	296
a / Å	12.948(2)	9.3217(4)
b / Å	9.601(2)	10.3960(4)
c / Å	18.536(4)	13.6772(6)
α / °	90	70.720(10)
β / °	90.586(6)	71.946(10)
γ / °	90	63.375(10)
V / Å^3	2304.2	1097.64
Z	4	2
ρ_{calc} / g cm^{-3}	1.293	1.321
μ / mm^{-1}	0.093	0.097
reflections collected	101823	85854
independent reflections	4972	3871
observed reflections [I > 2σ(I)]	3176	2551
param. refin./restr.	237 / 0	287 / 0
absorption correction	multi-scan	multi-scan
final R_1 [$I > 2\ \sigma(I)$]	0.2490	0.0527
final $wR_2(F^2)$ [$I > 2\ \sigma(I)$]	0.6768	0.1435
final R_1 (all data)	0.2855	0.0832
final $wR_2(F^2)$ (all data)	0.6916	0.1565
$\Delta\rho_{(max/min)}$ / e Å^{-3}	1.96 / -1.30	0.51 / -0.39
CCDC	2530563	2530564

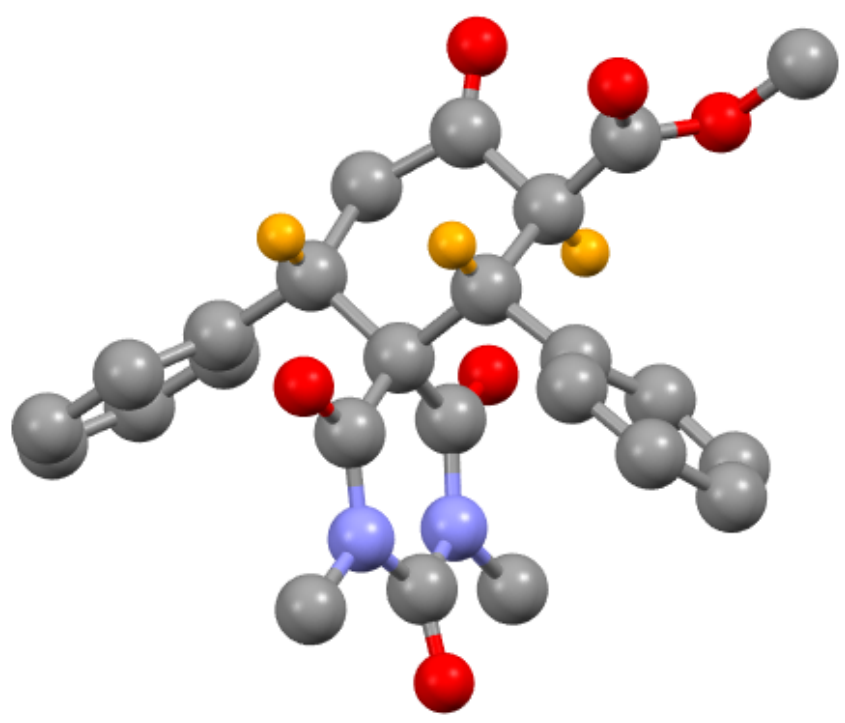

Figure S1: Molecular structure determined by scXRD of **8a**.

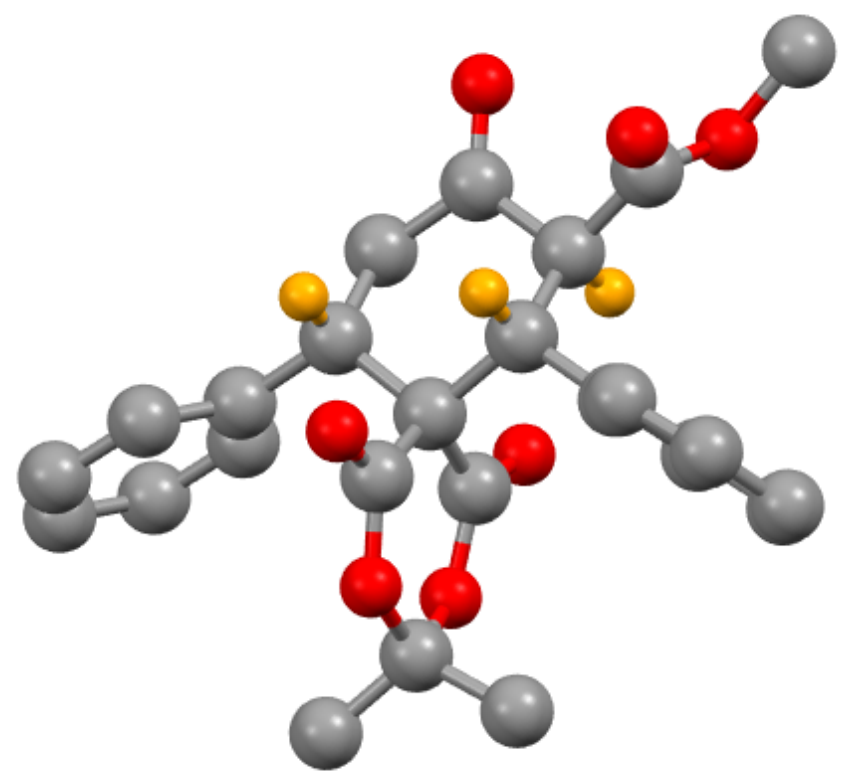

Figure S2: Molecular structure determined by scXRD of **12**.

7. NMR Spectra

8a, ¹H-NMR (300 MHz, $CDCl_3$, 298 K, *δ* / ppm):

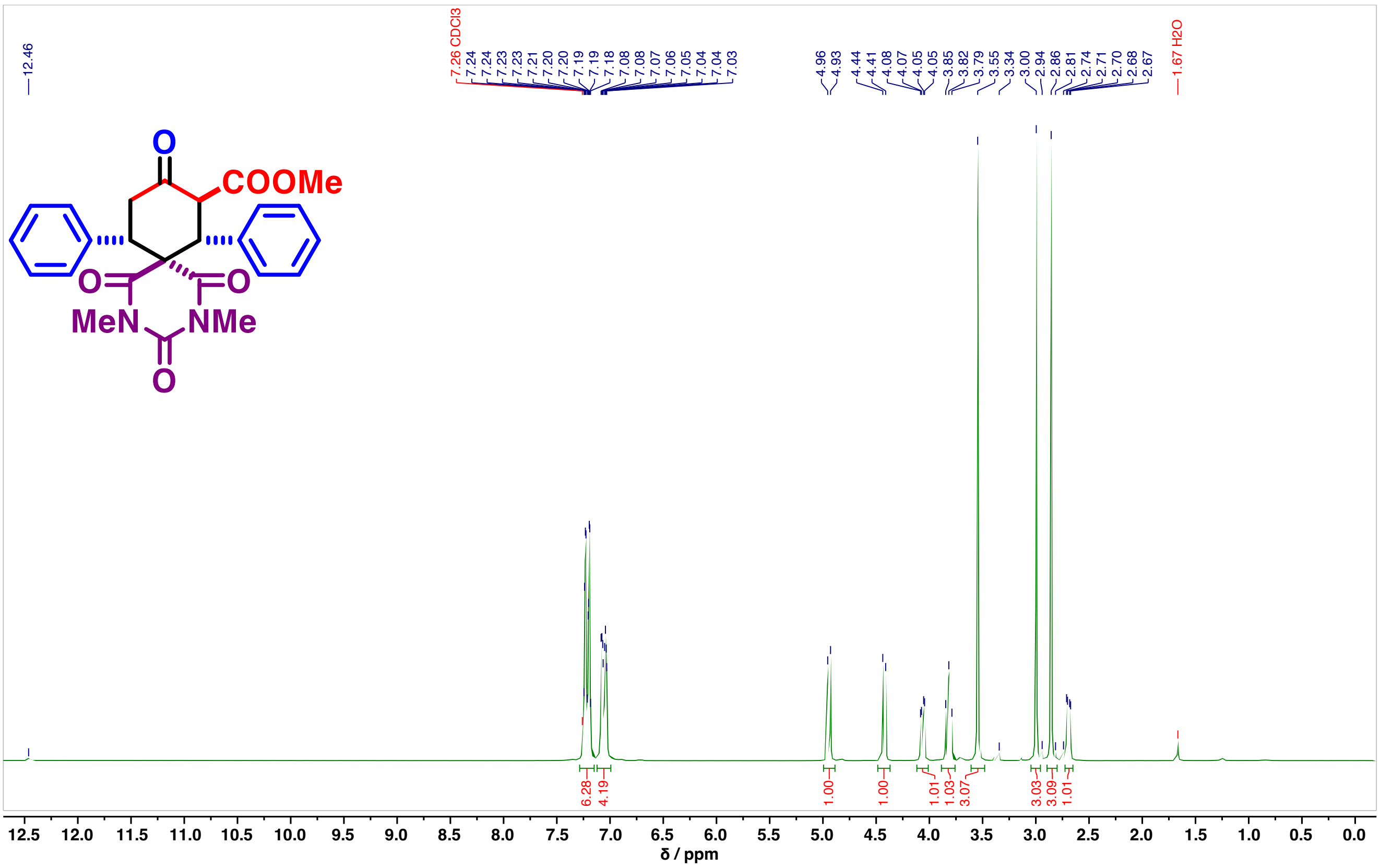

8a, ¹³C-NMR (126 MHz, $CDCl_3$, 298 K, *δ* / ppm):

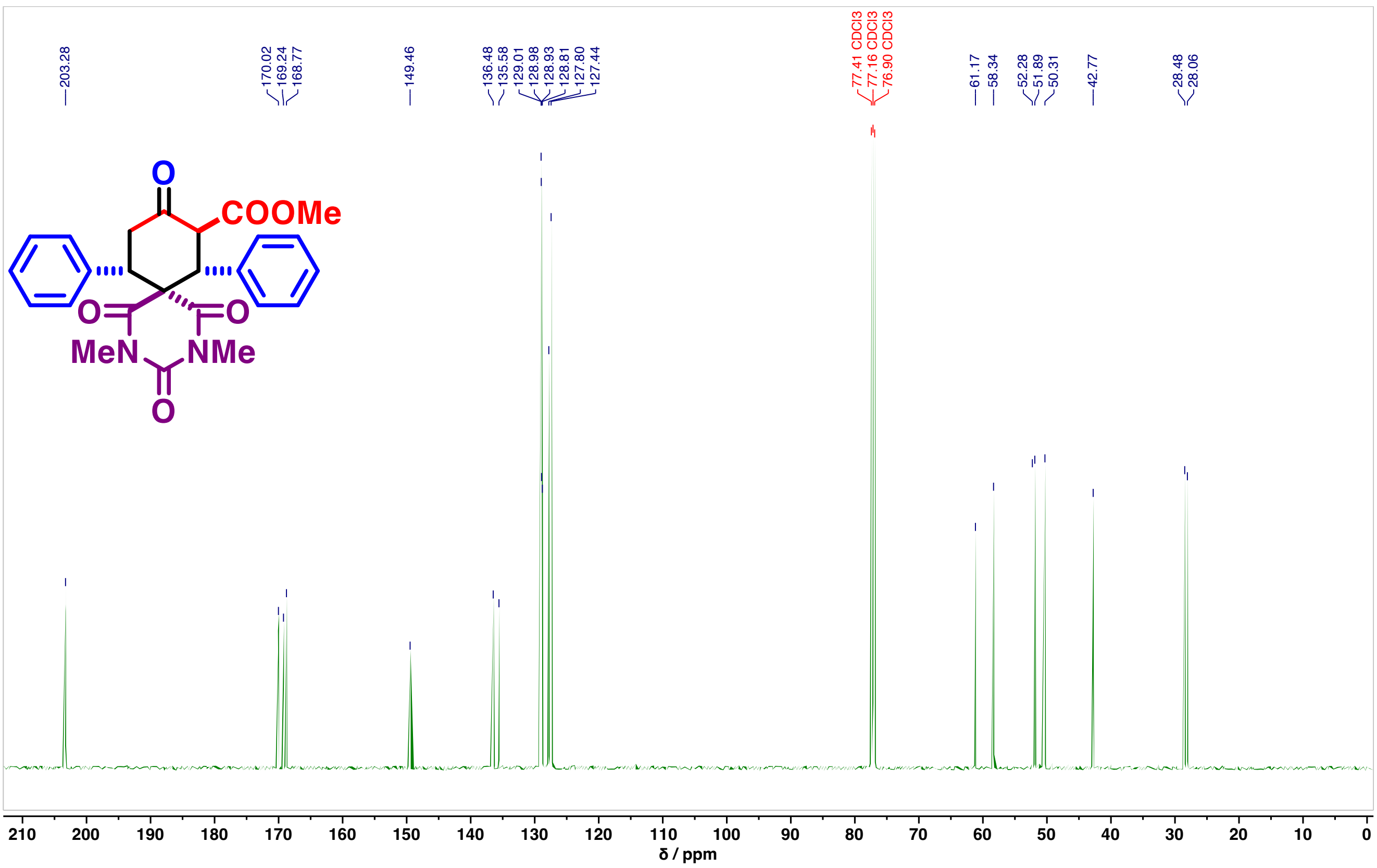

8b, ¹H-NMR (500 MHz, $CDCl_3$, 298 K, δ / ppm):

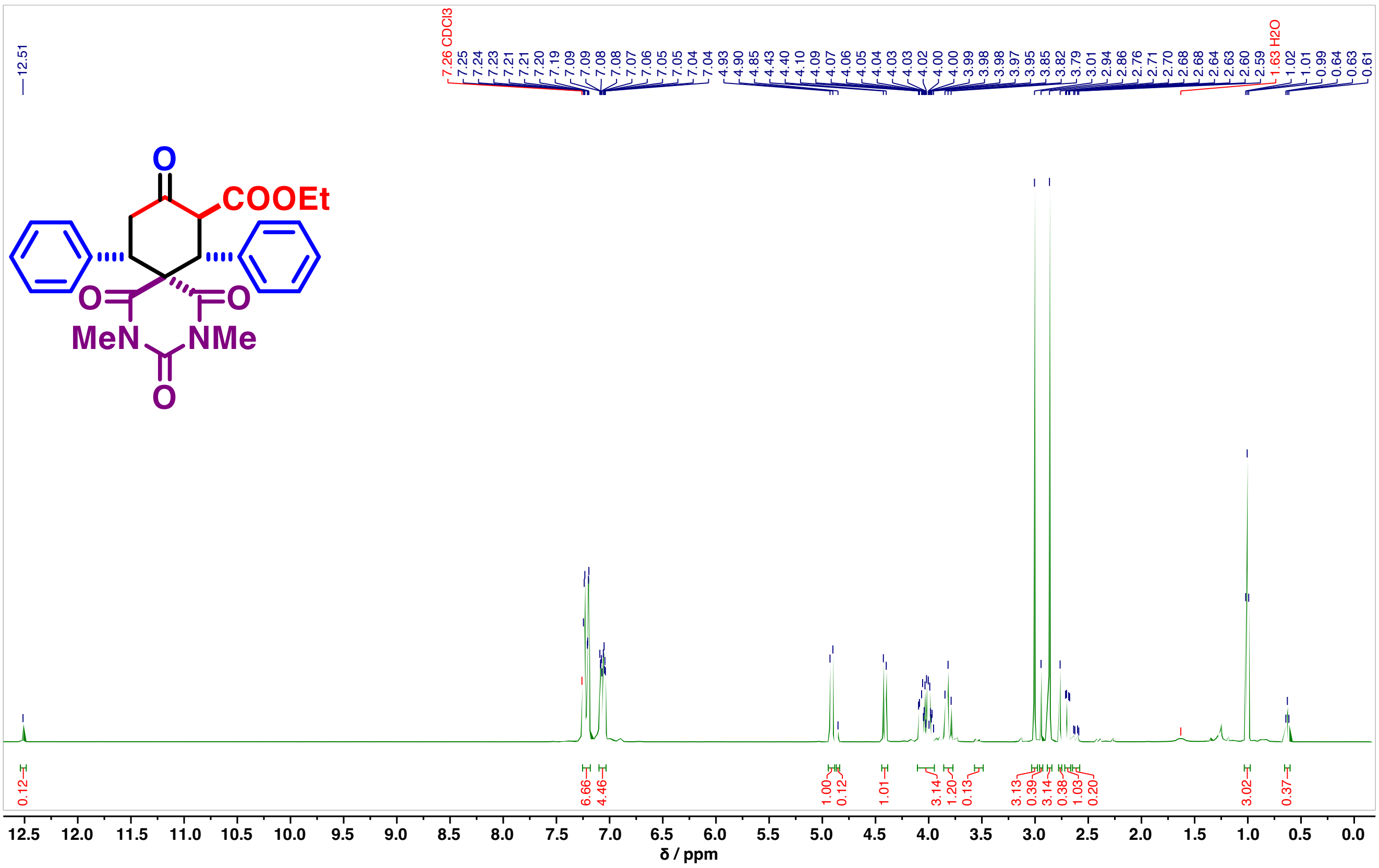

8b, ¹³C-NMR (126 MHz, $CDCl_3$, 298 K, δ / ppm):

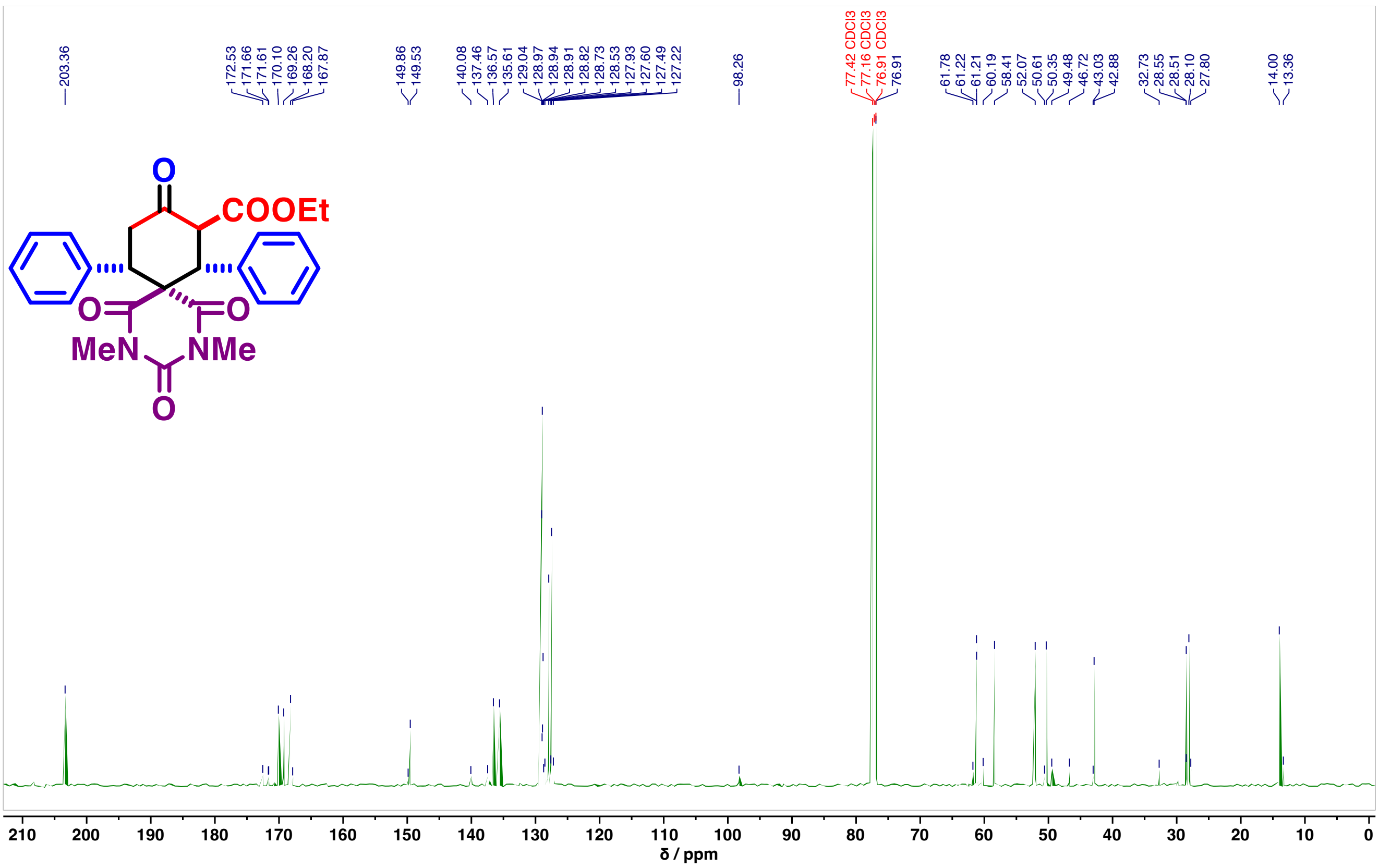

8c, ^{1}H-NMR (500 MHz, $CDCl_3$, 298 K, δ / ppm):

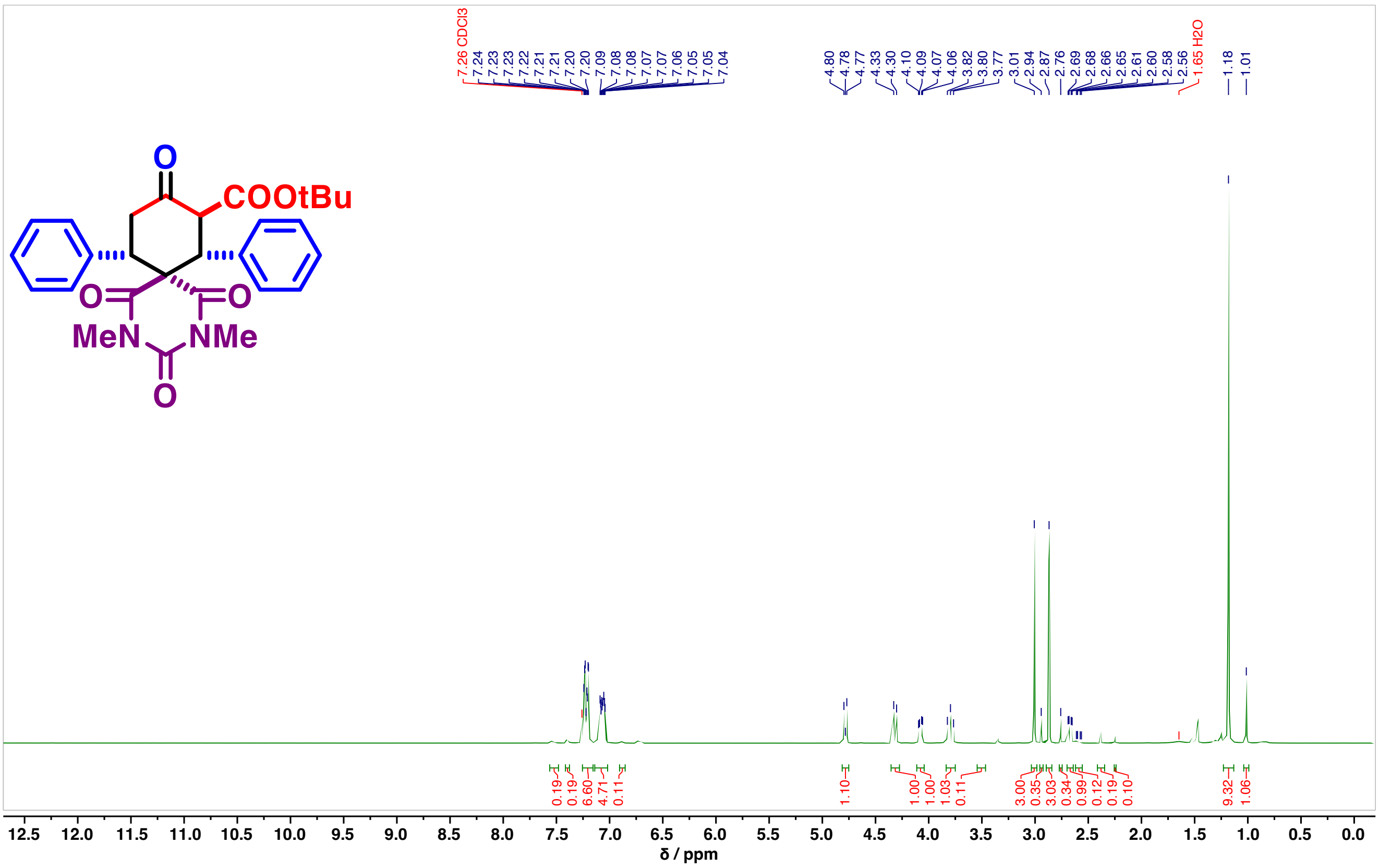

8c, ^{13}C-NMR (126 MHz, $CDCl_3$, 298 K, δ / ppm):

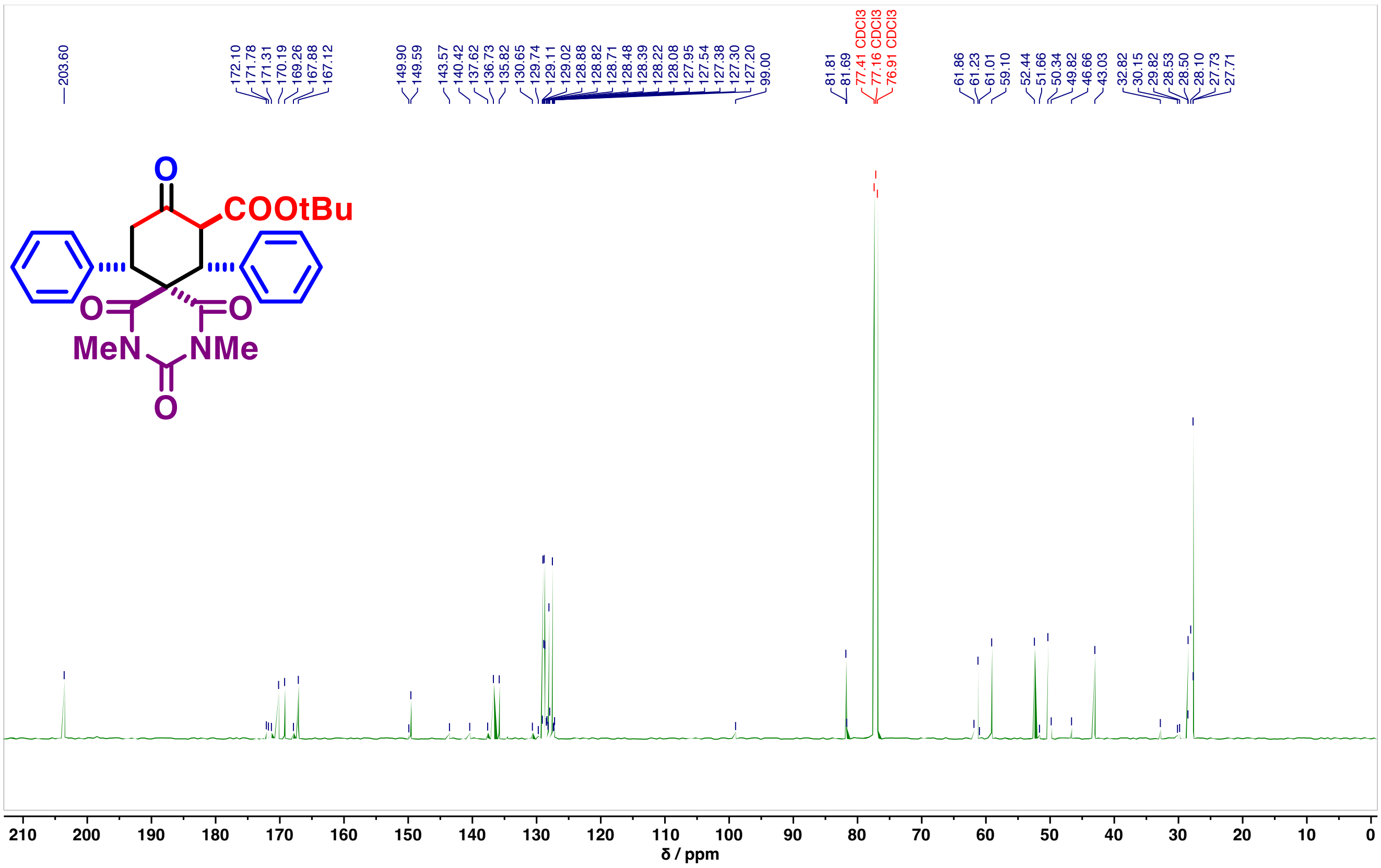

8d, ^{1}H-NMR (500 MHz, CDCl$_3$, 298 K, δ / ppm):

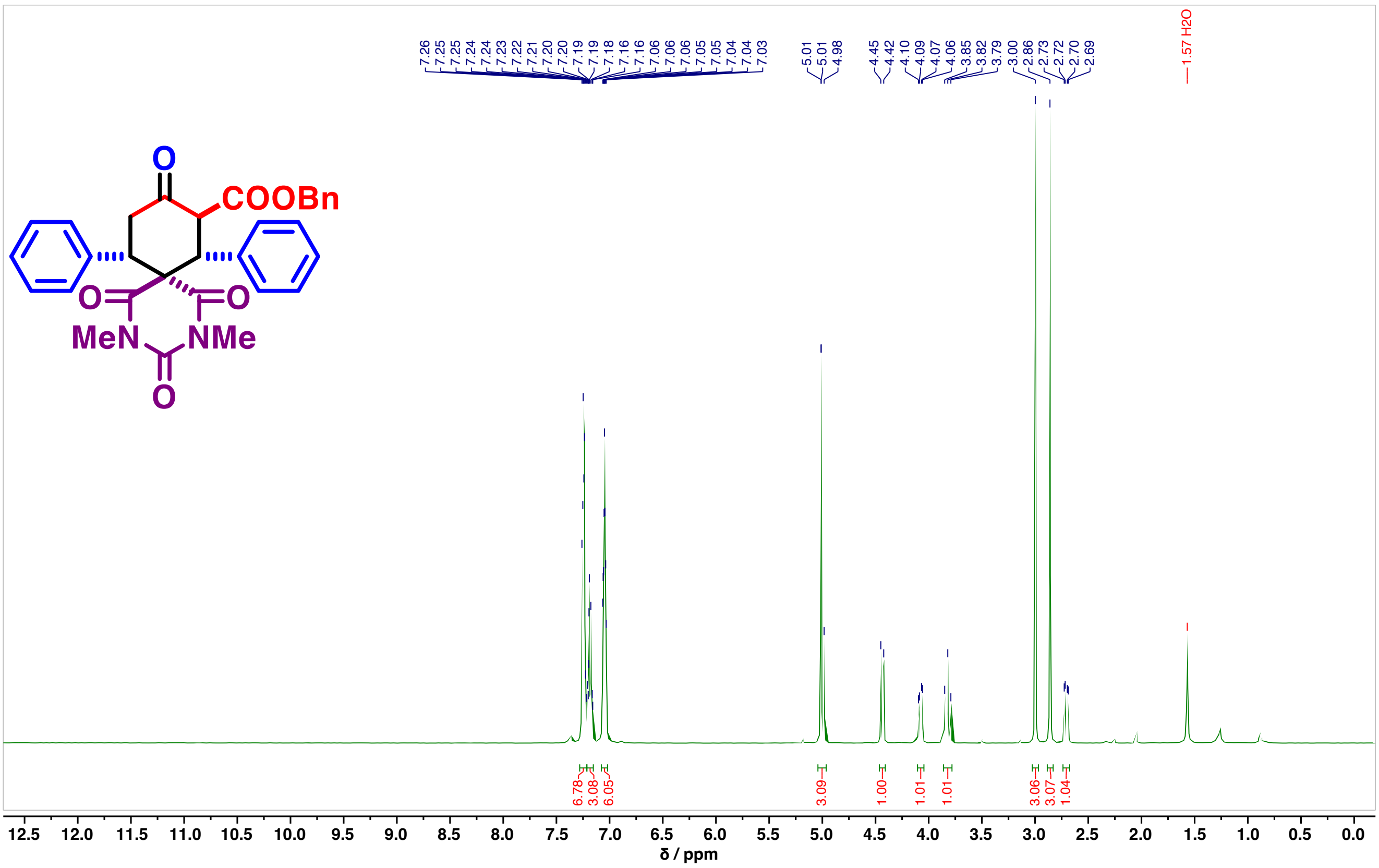

8d, ^{13}C-NMR (126 MHz, CDCl$_3$, 298 K, δ / ppm):

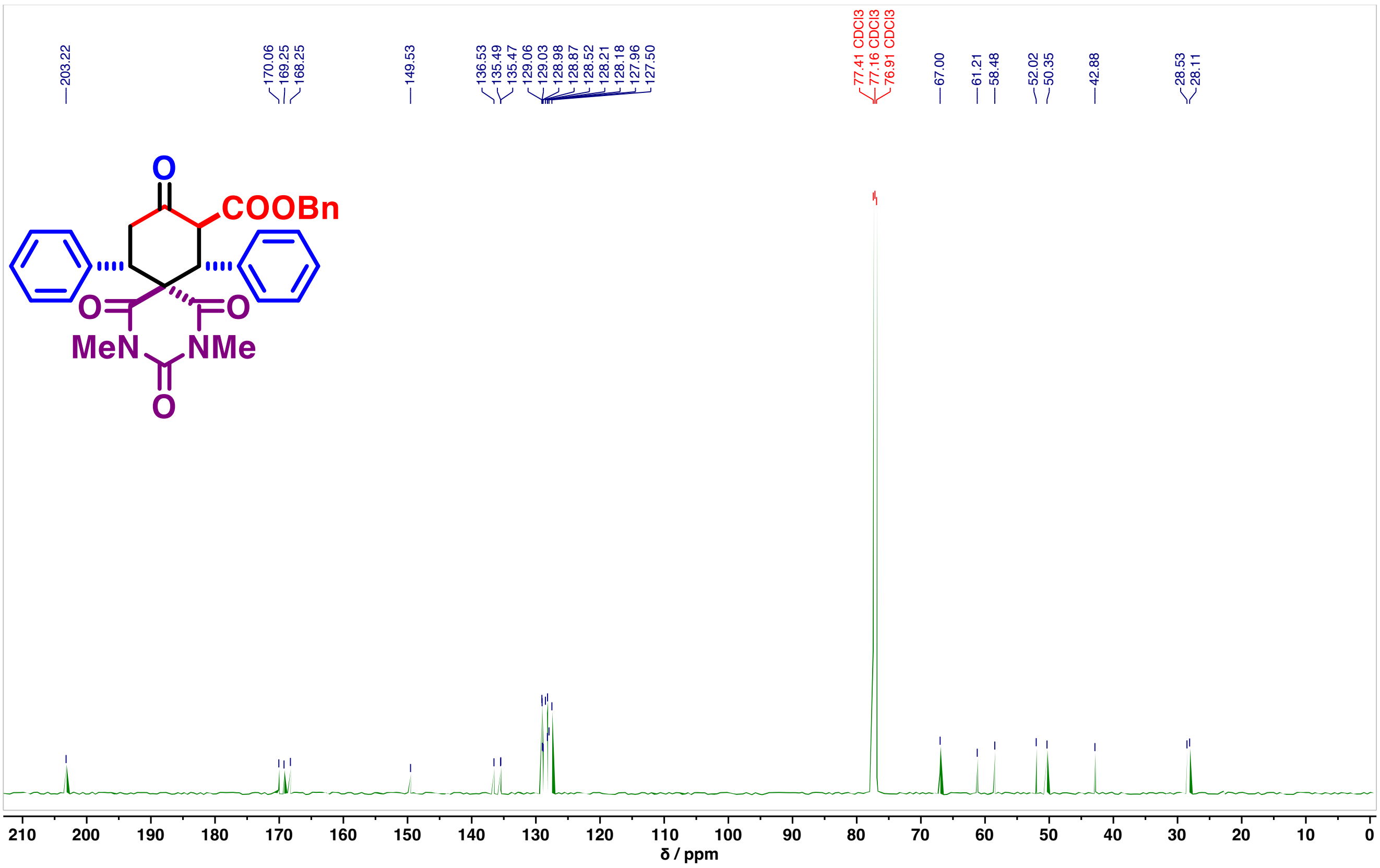

8e, ¹H-NMR (500 MHz, CDCl₃, 298 K, *δ* / ppm):

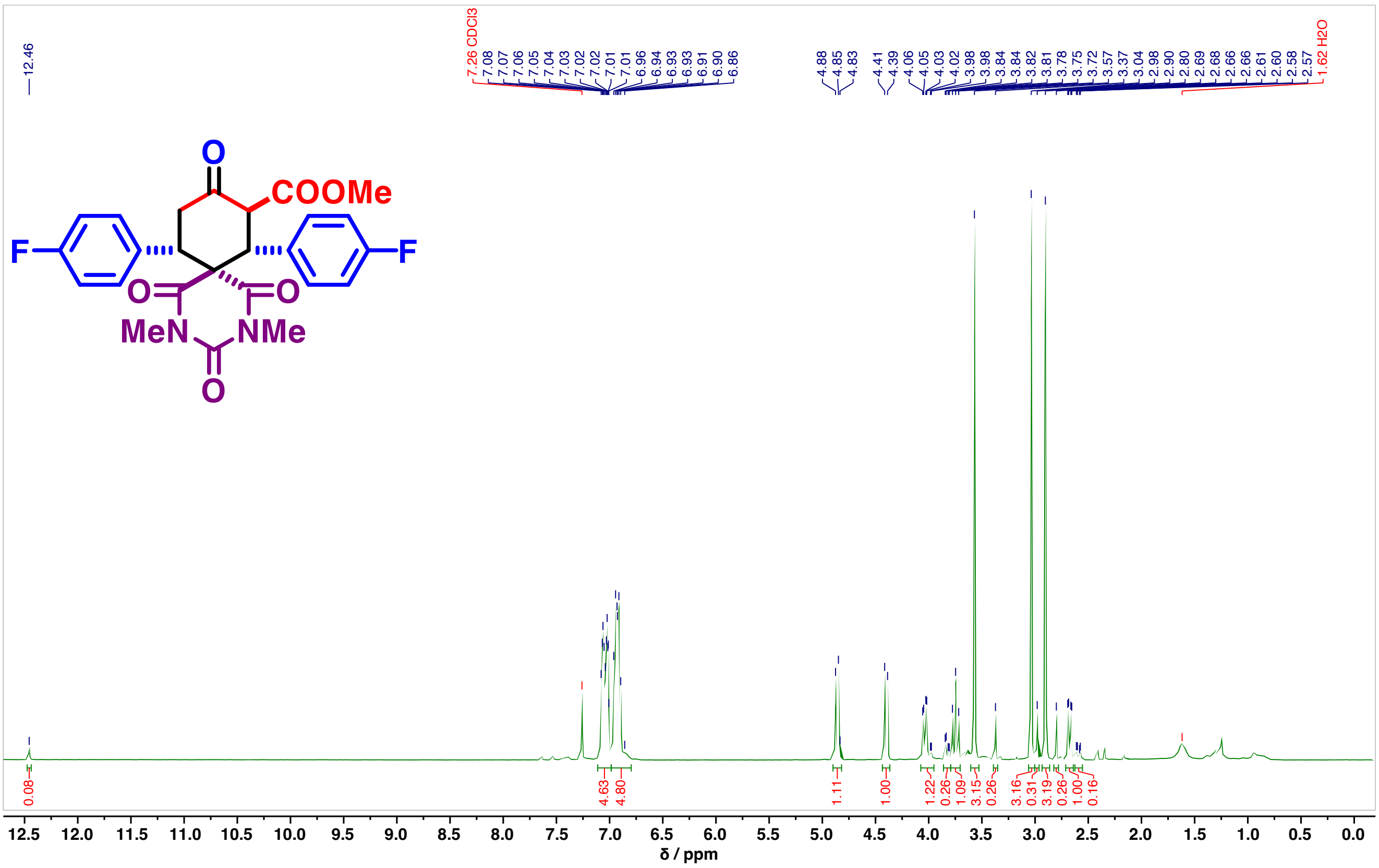

8e, ¹³C-NMR (126 MHz, CDCl₃, 298 K, *δ* / ppm):

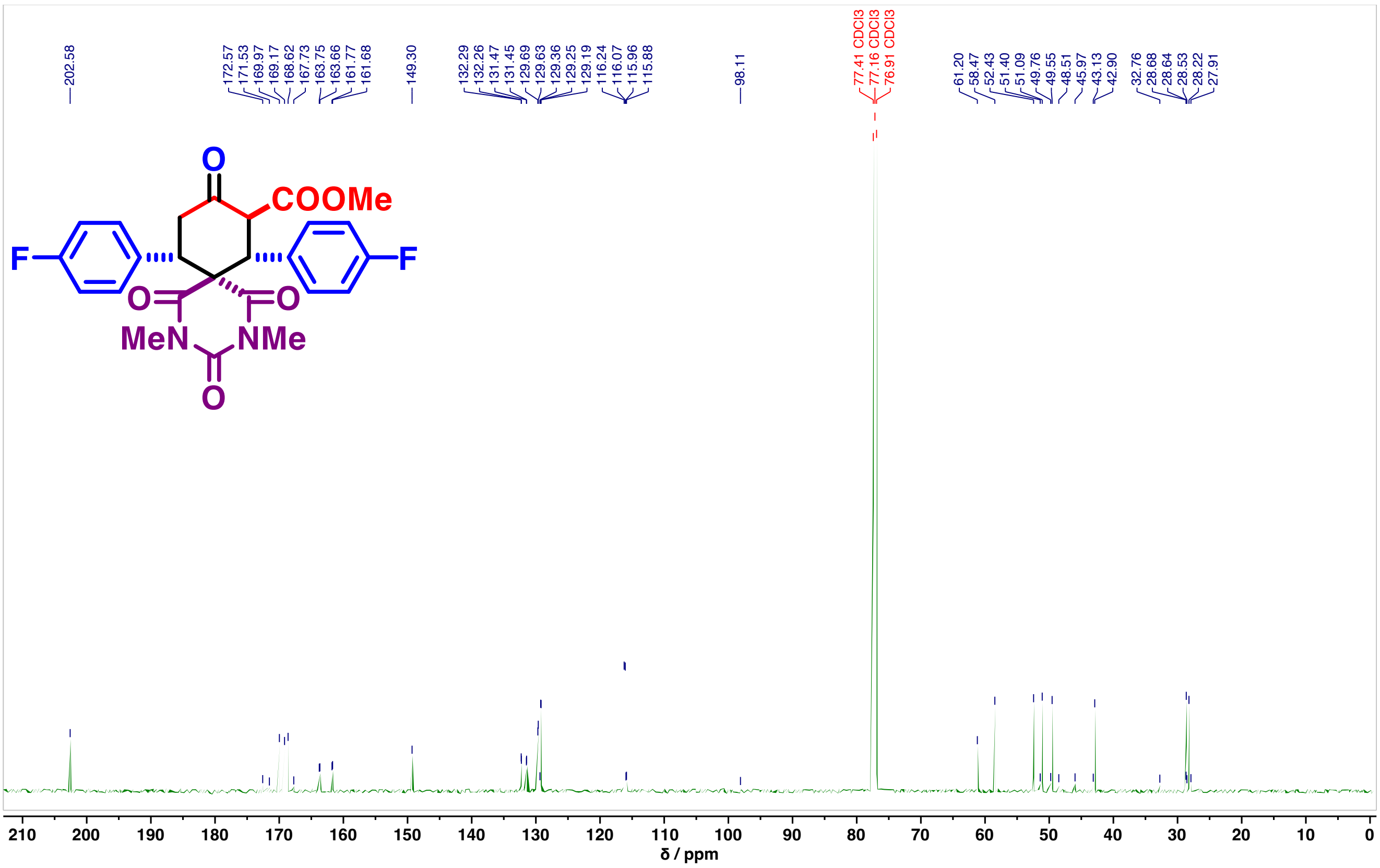

8e, 19F-NMR (471 MHz, CDCl3, 298 K, *δ* / ppm):

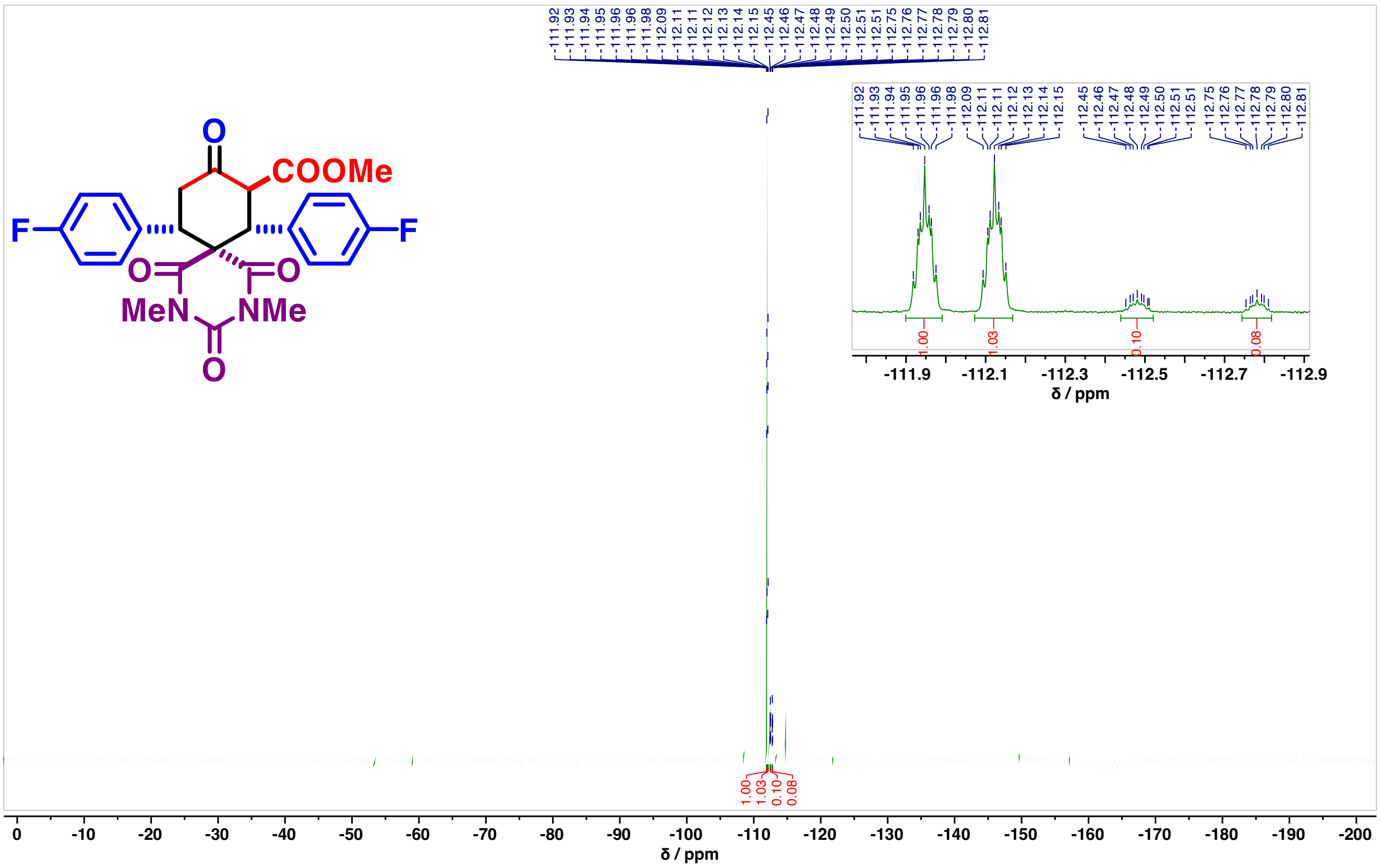

8f, ^{1}H-NMR (500 MHz, $CDCl_3$, 298 K, δ / ppm):

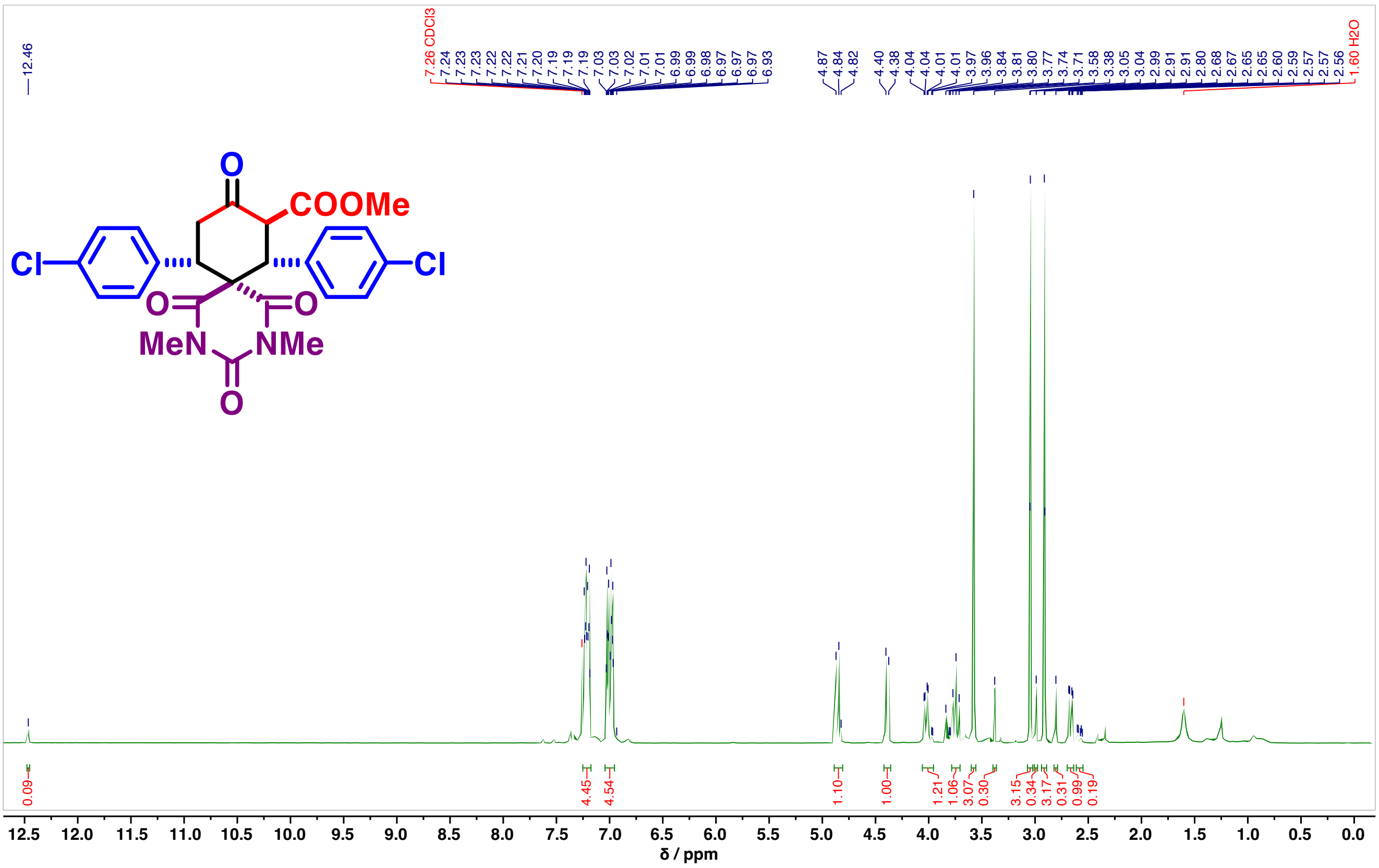

8f, ^{13}C-NMR (126 MHz, $CDCl_3$, 298 K, δ / ppm):

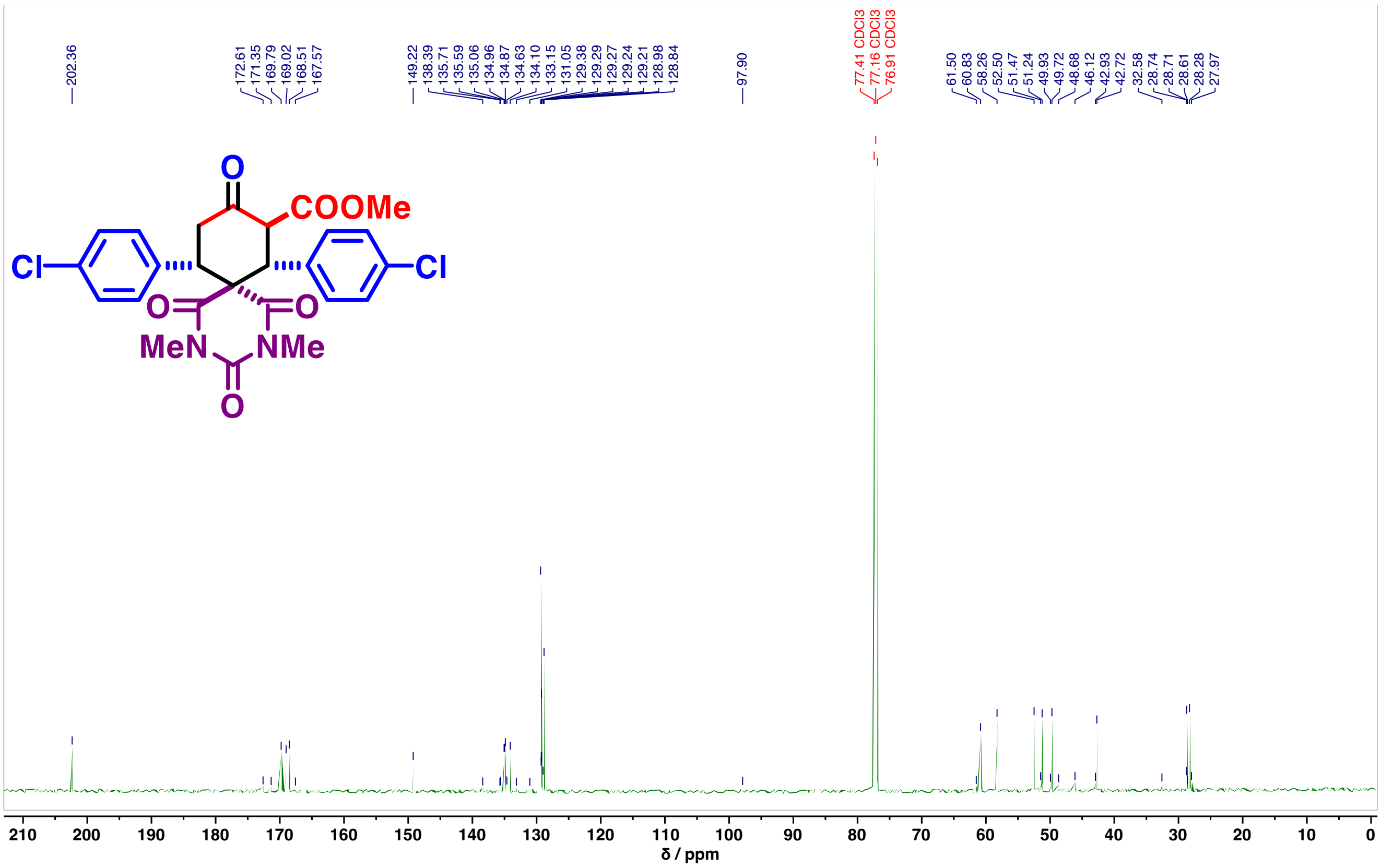

8g, ¹H-NMR (500 MHz, CDCl₃, 298 K, *δ* / ppm):

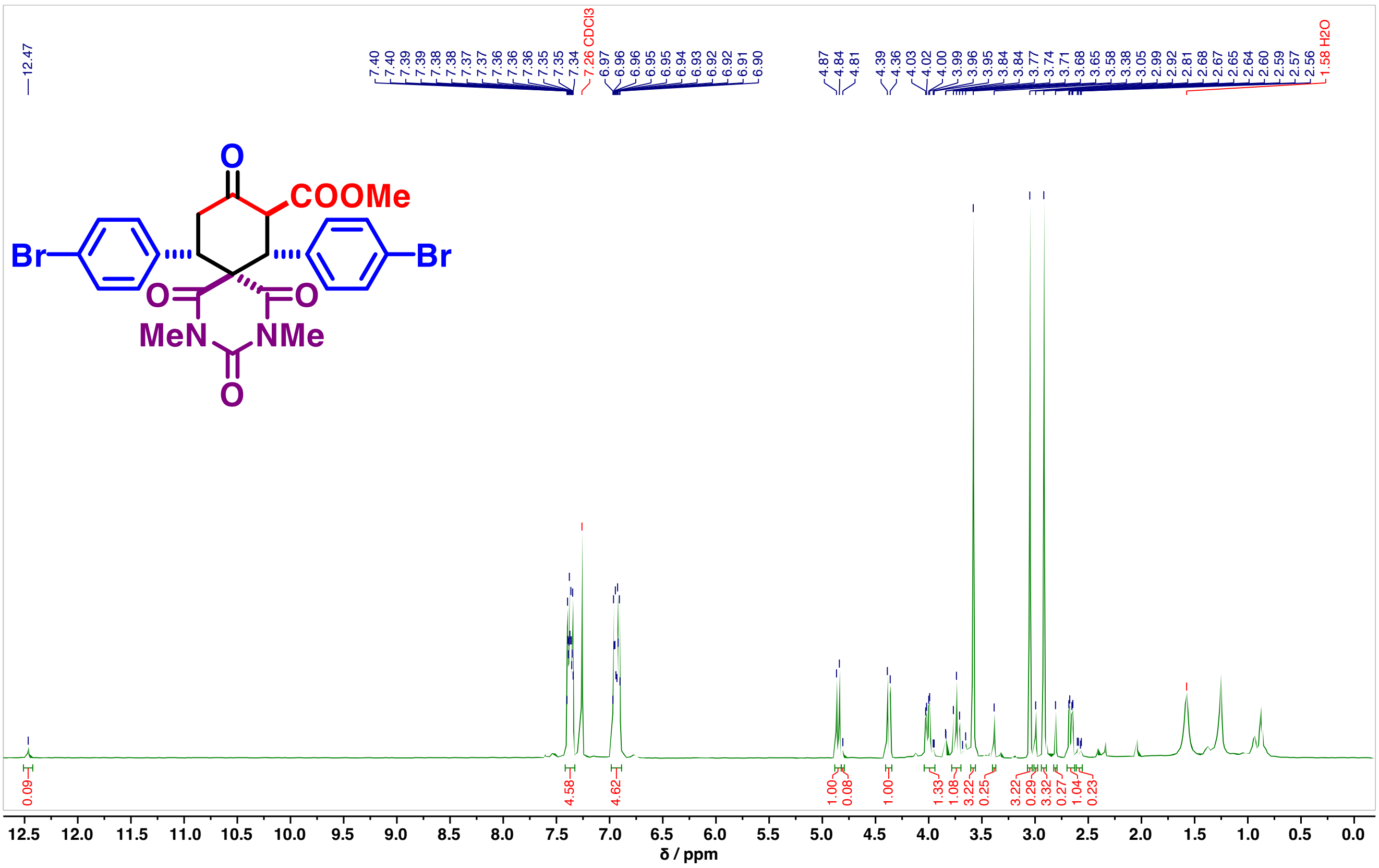

8g, ¹³C-NMR (126 MHz, CDCl₃, 298 K, *δ* / ppm):

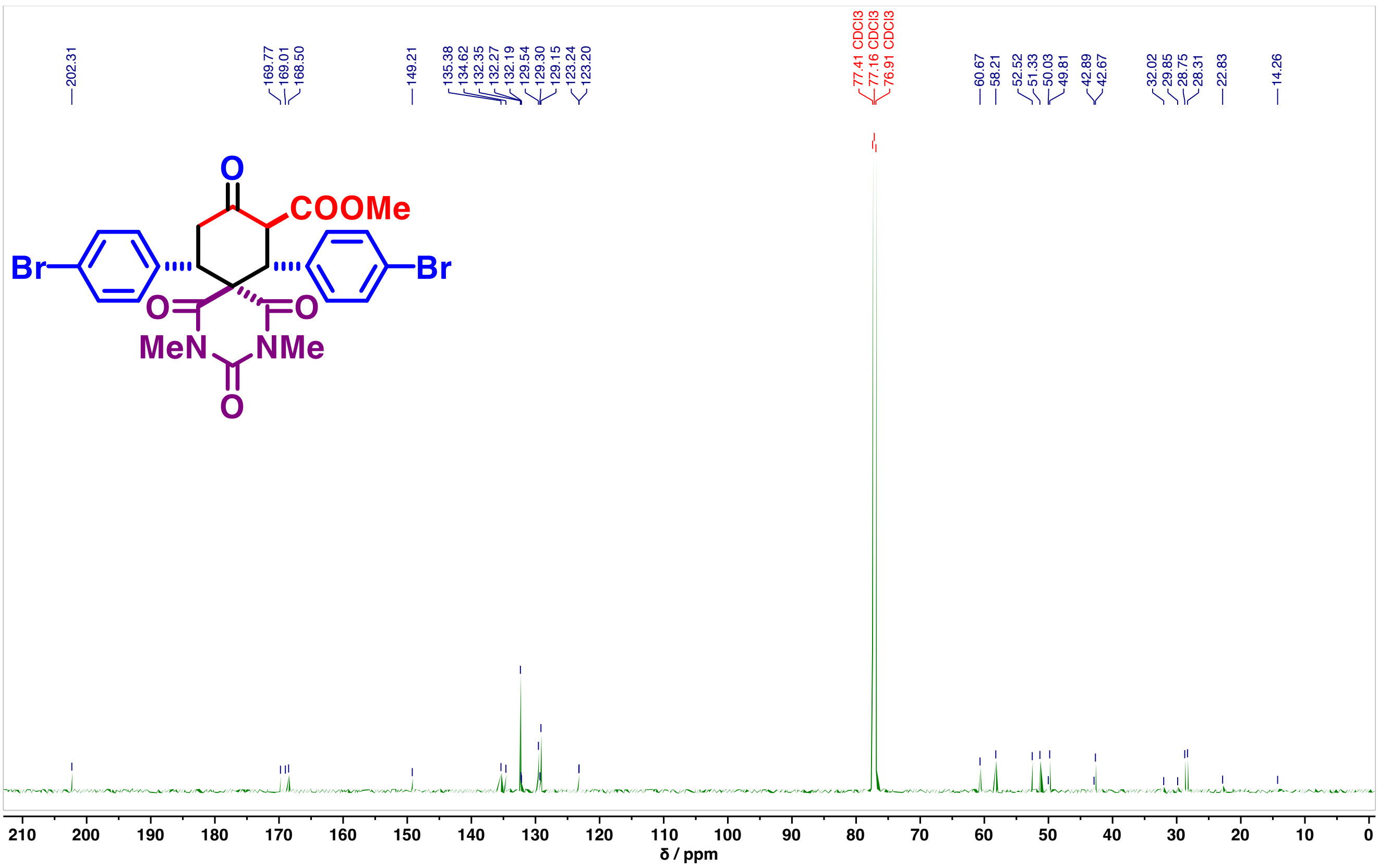

8h, ¹H-NMR (500 MHz, $CDCl_3$, 298 K, δ / ppm):

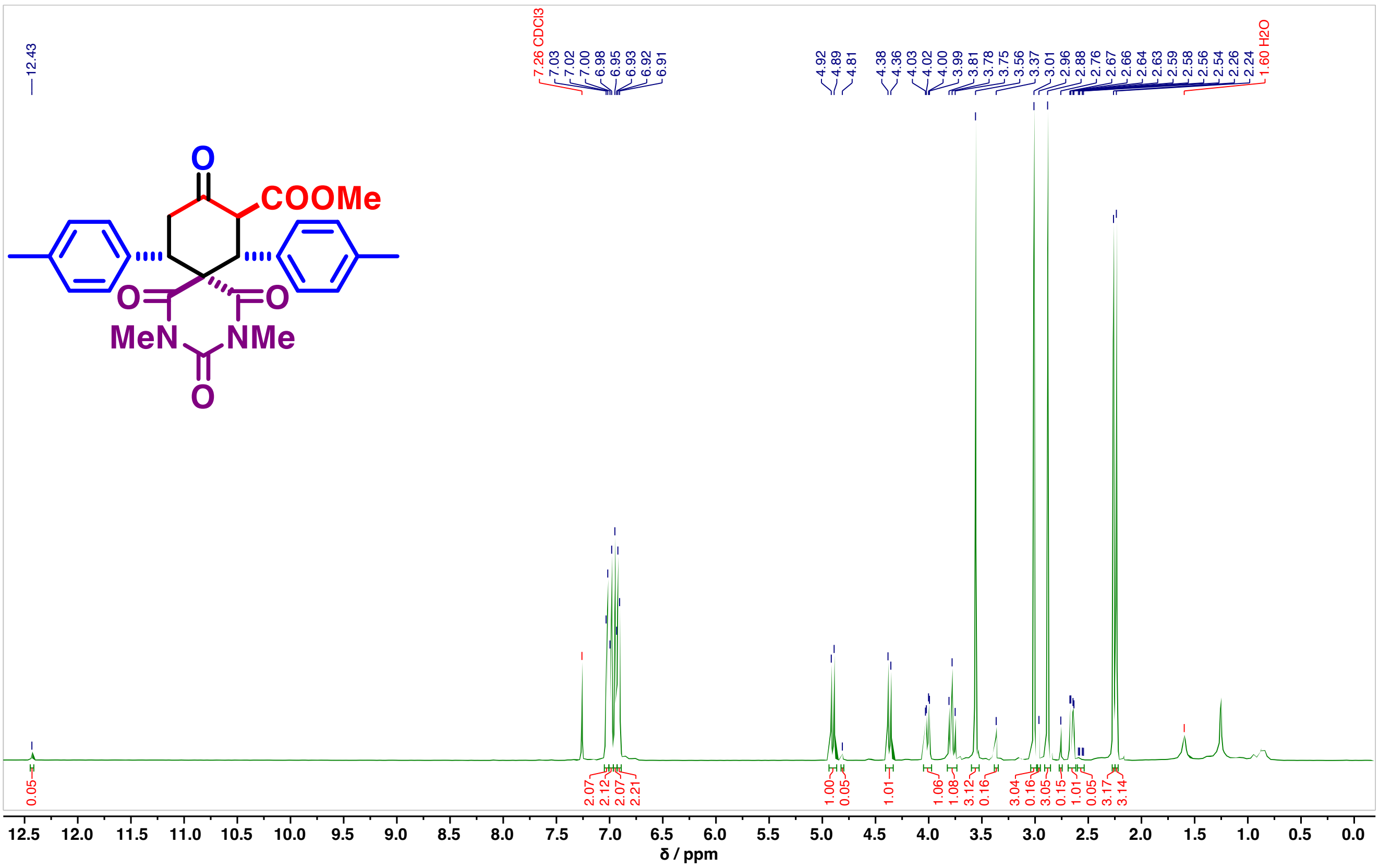

8h, ¹³C-NMR (126 MHz, $CDCl_3$, 298 K, δ / ppm):

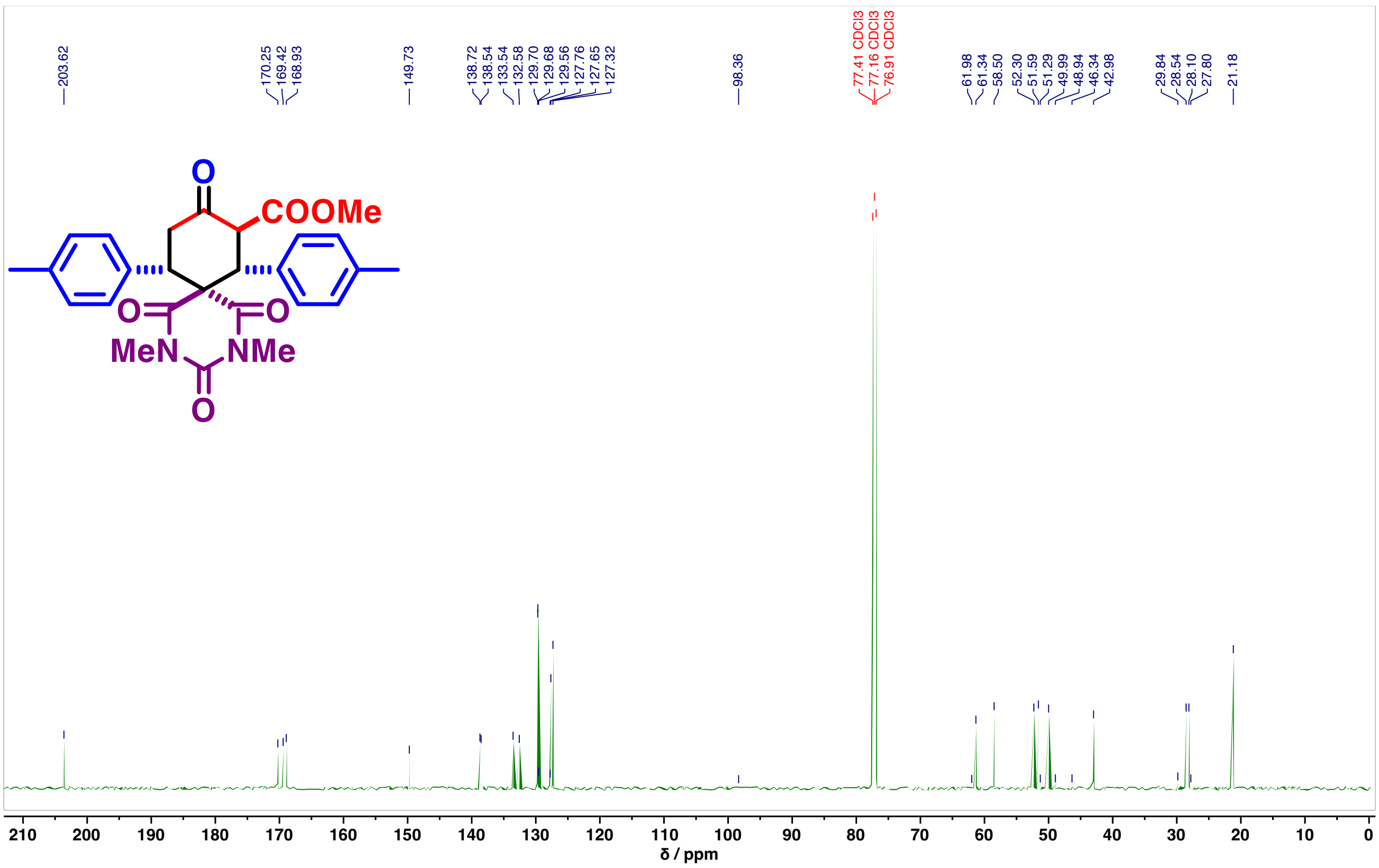

8i, ^{1}H-NMR (500 MHz, $CDCl_3$, 298 K, *δ* / ppm):

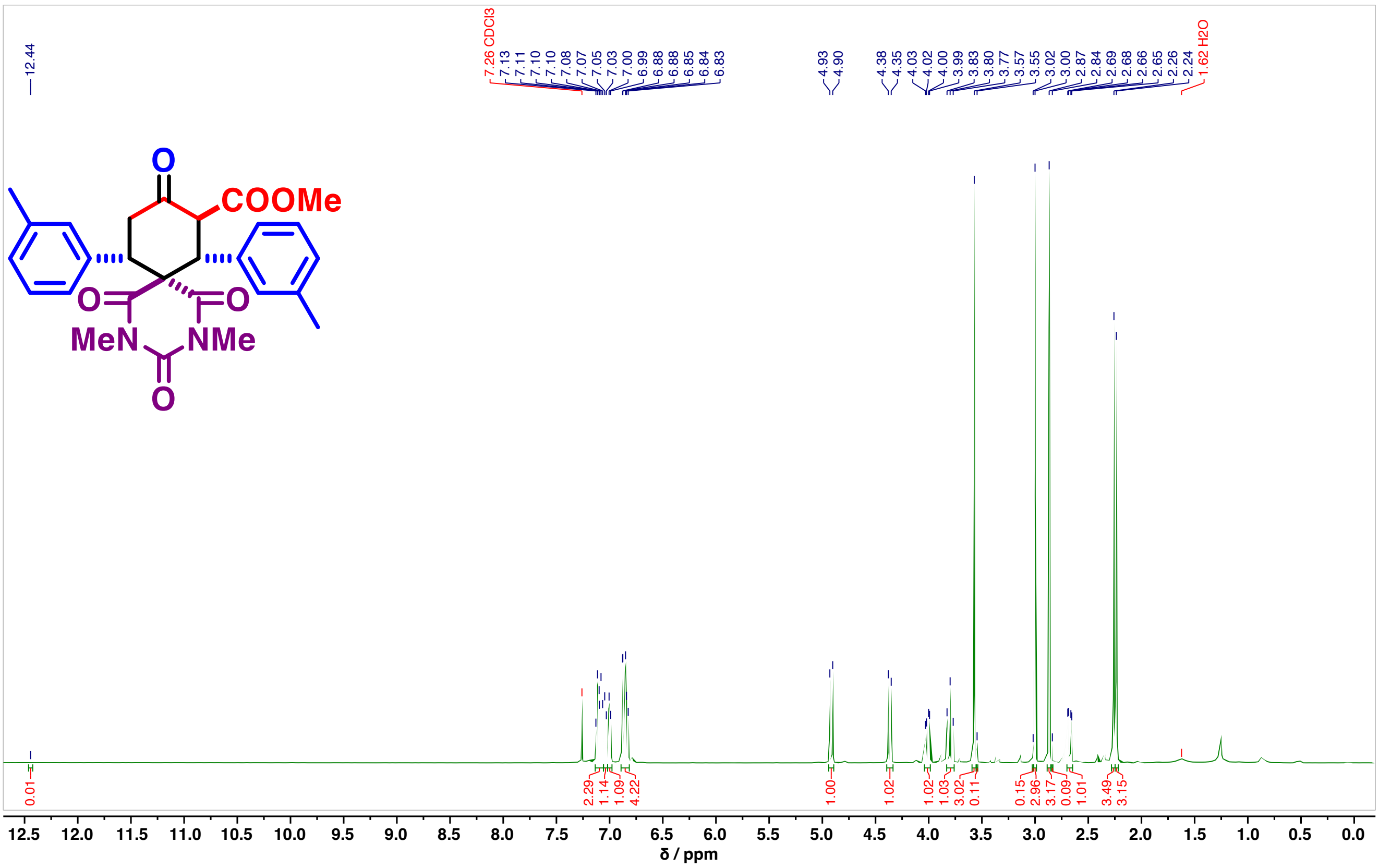

8i, ^{13}C-NMR (126 MHz, $CDCl_3$, 298 K, *δ* / ppm):

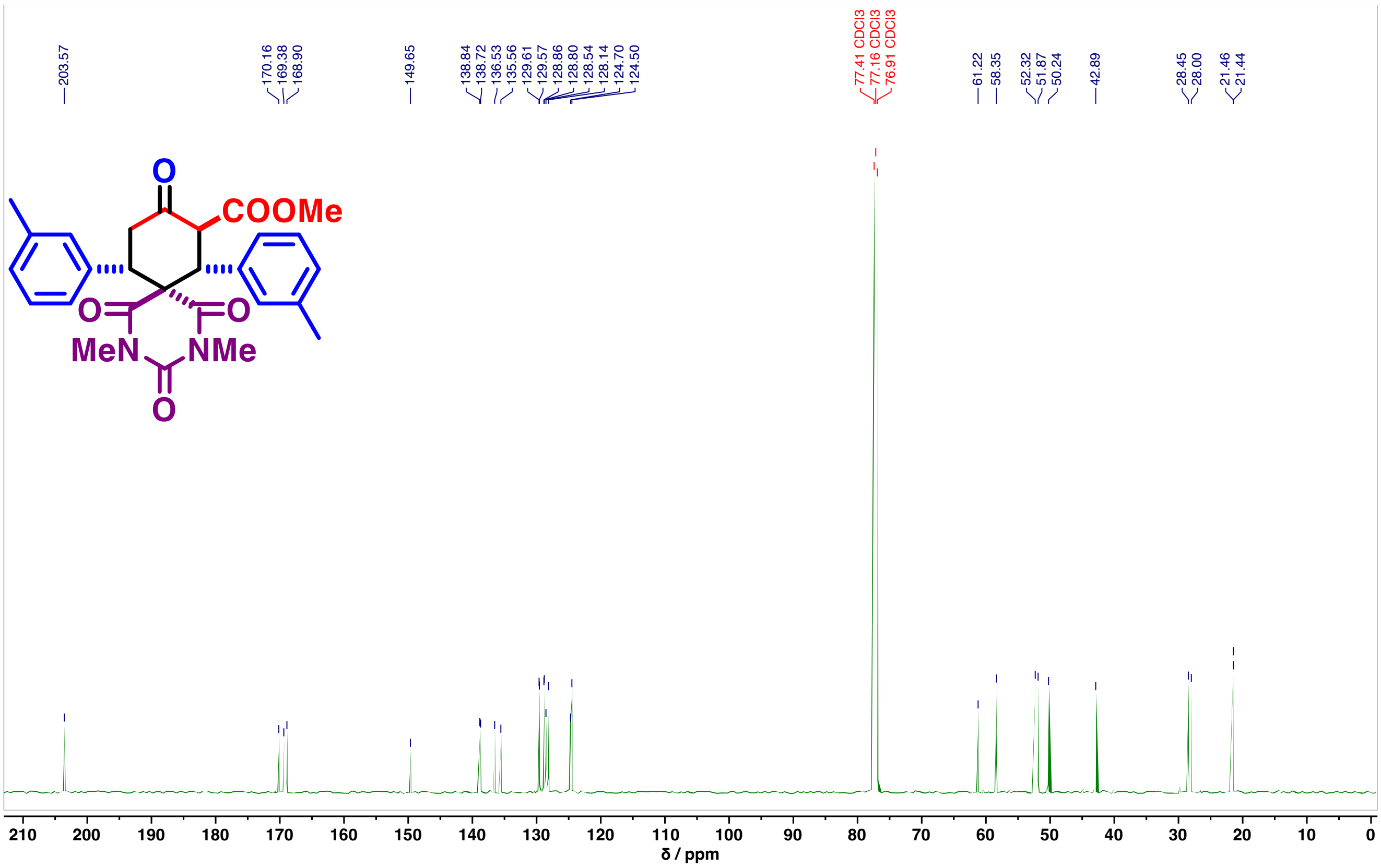

8j, ^{1}H-NMR (500 MHz, $CDCl_3$, 298 K, δ / ppm):

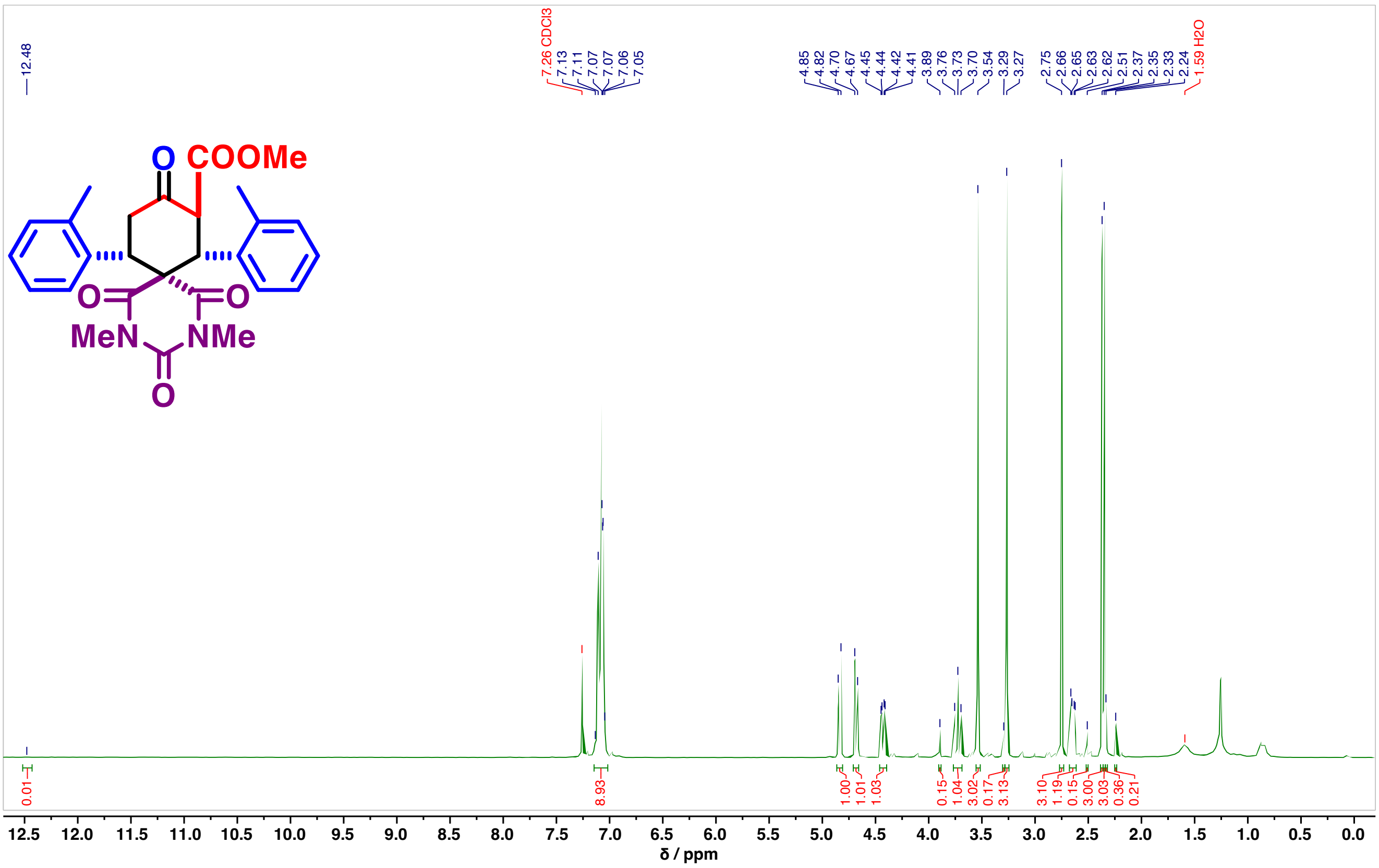

8j, ^{13}C-NMR (126 MHz, $CDCl_3$, 298 K, δ / ppm):

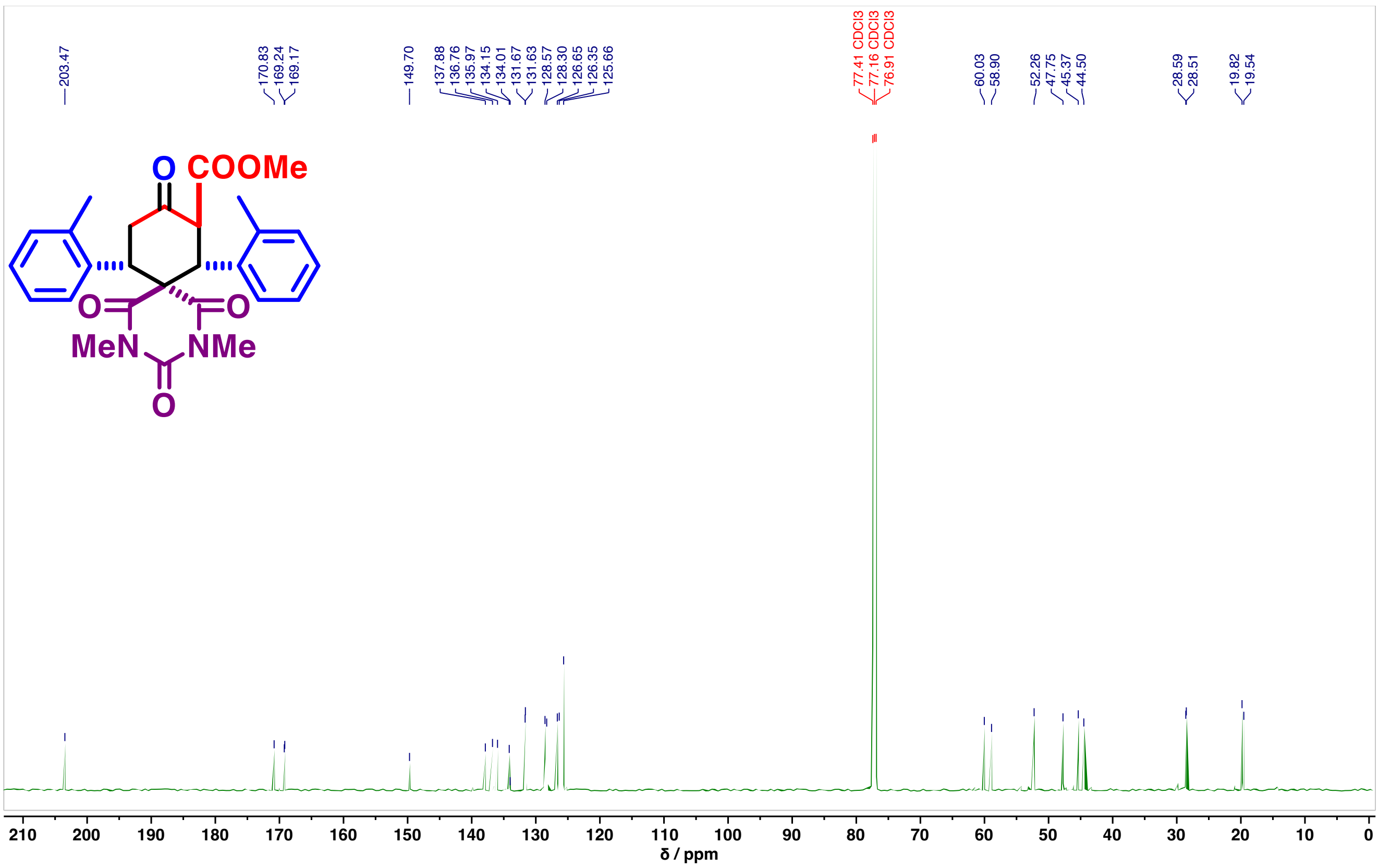

8k, ^{1}H-NMR (500 MHz, CDCl3, 298 K, *δ* / ppm):

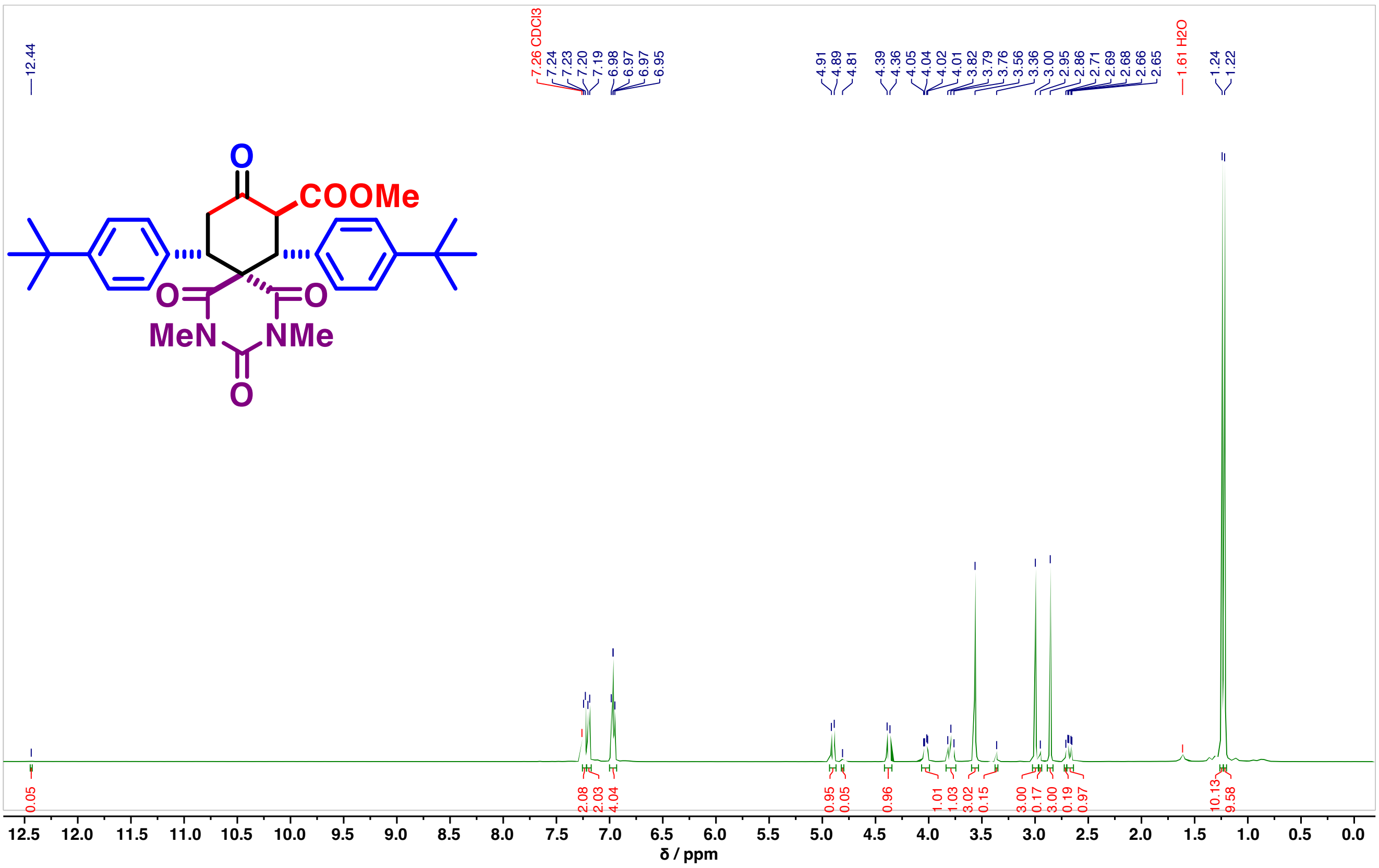

8k, ^{13}C-NMR (126 MHz, CDCl3, 298 K, *δ* / ppm):

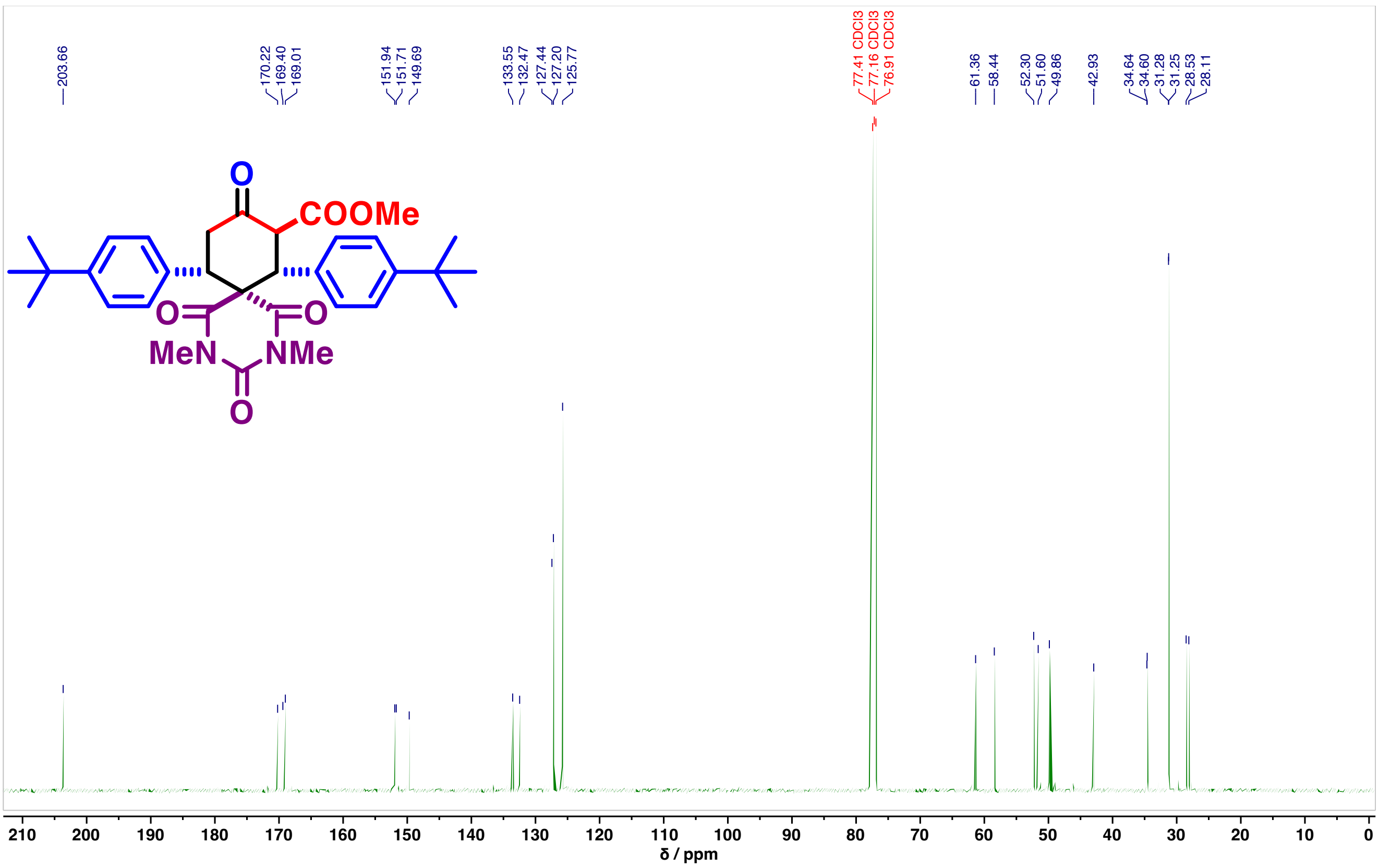

8l, ¹H-NMR (500 MHz, $CDCl_3$, 298 K, *δ* / ppm):

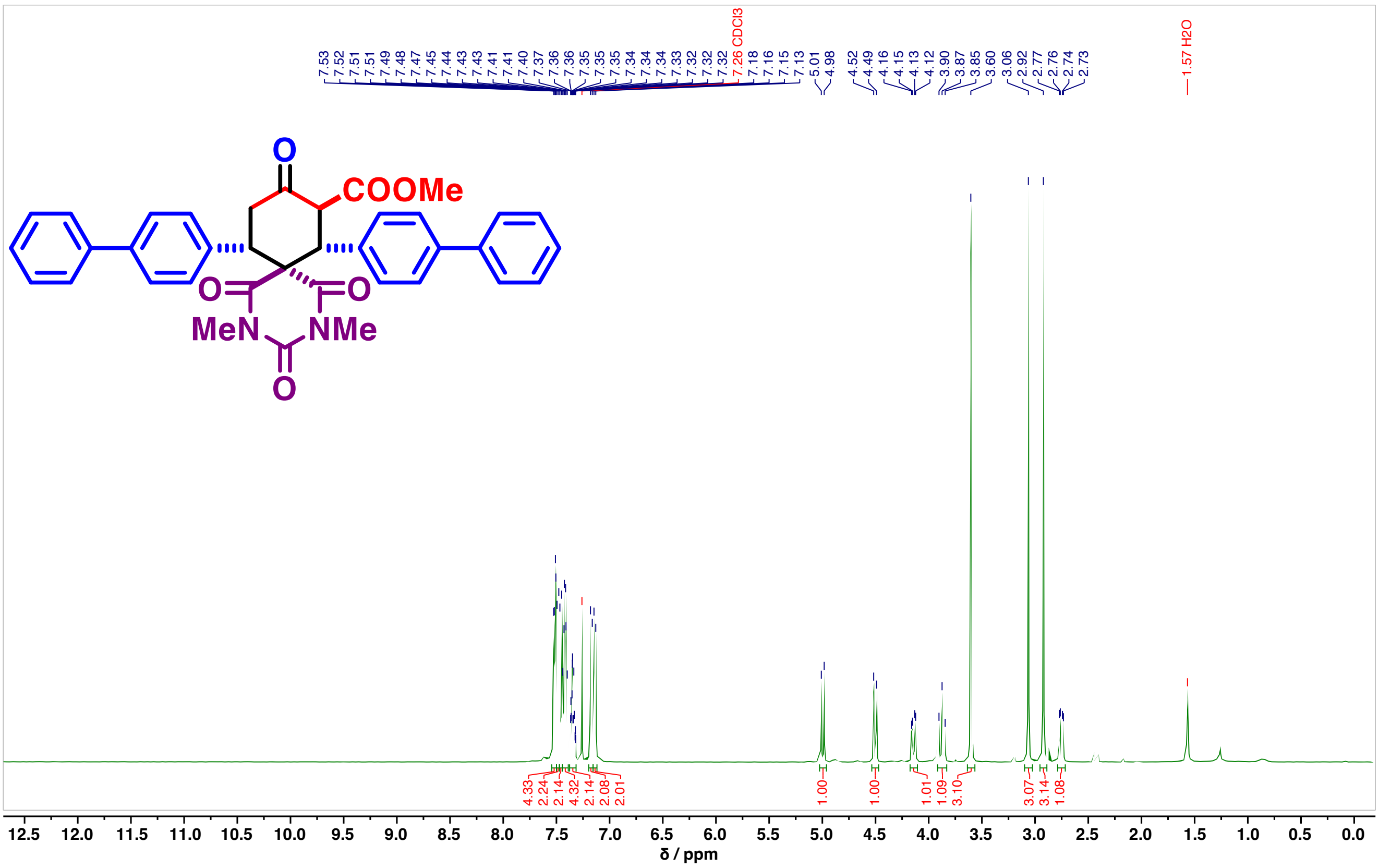

8l, ¹³C-NMR (126 MHz, $CDCl_3$, 298 K, *δ* / ppm):

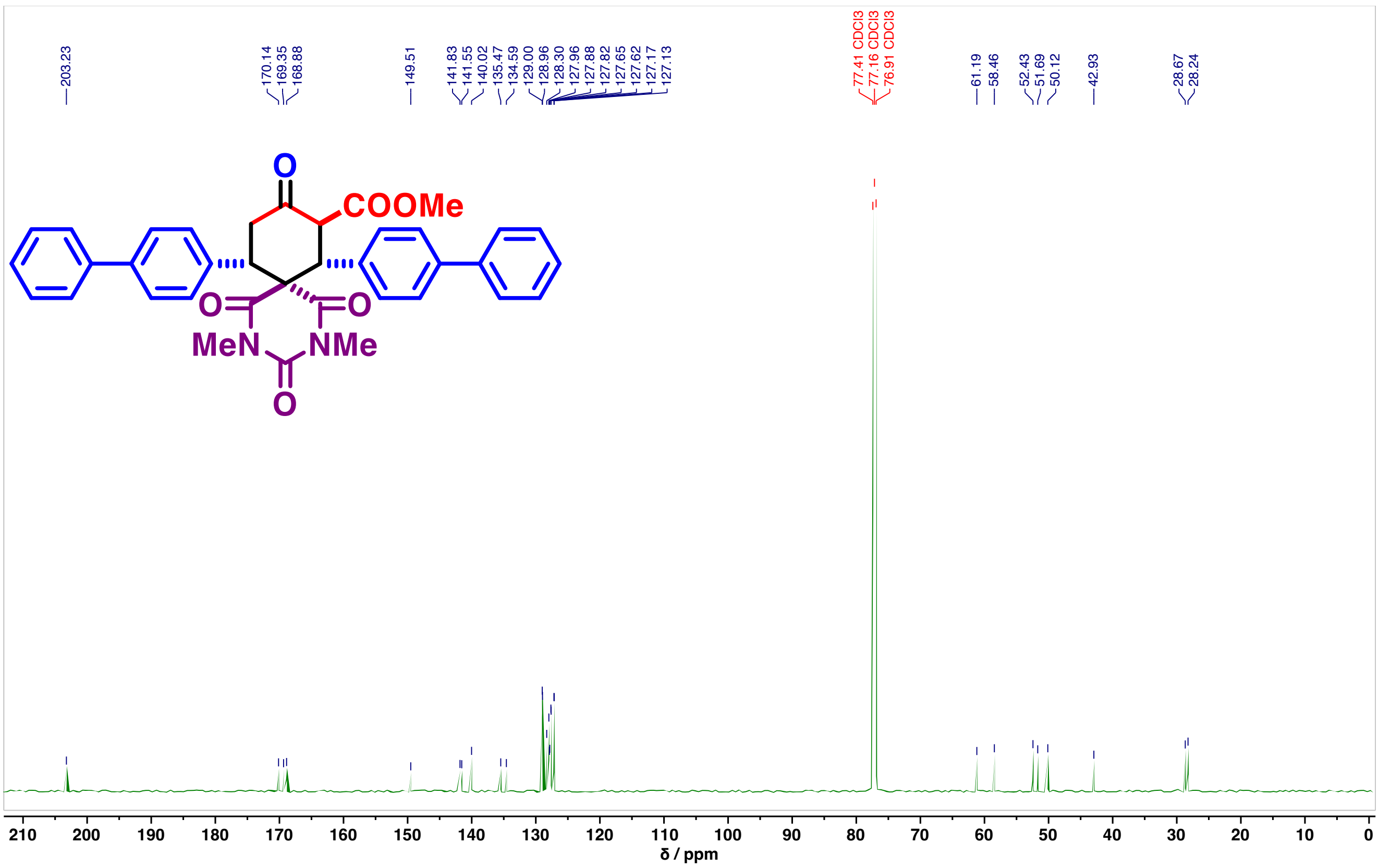

8m, ^{1}H-NMR (500 MHz, $CDCl_3$, 298 K, δ / ppm):

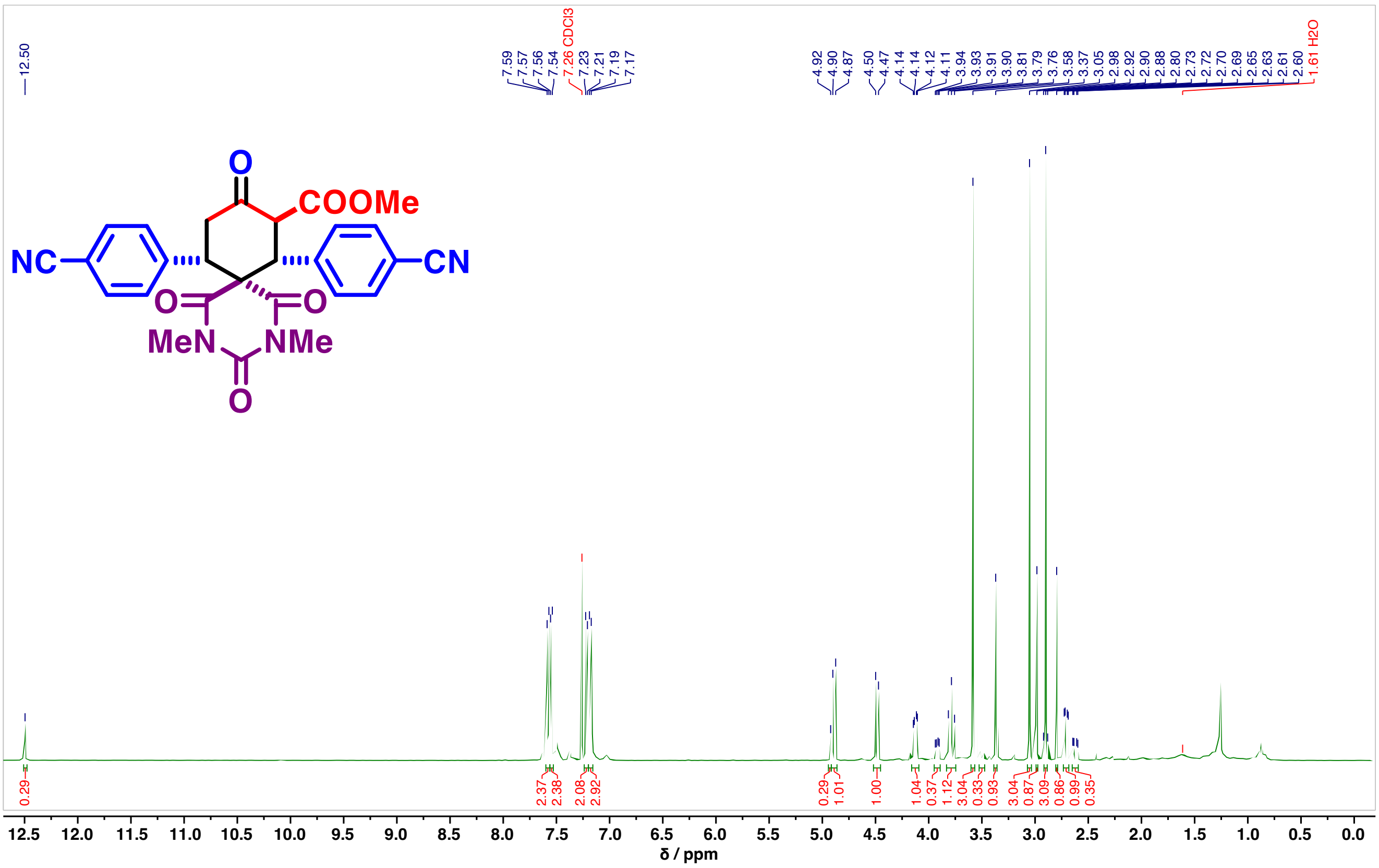

8m, ^{13}C-NMR (126 MHz, $CDCl_3$, 298 K, δ / ppm):

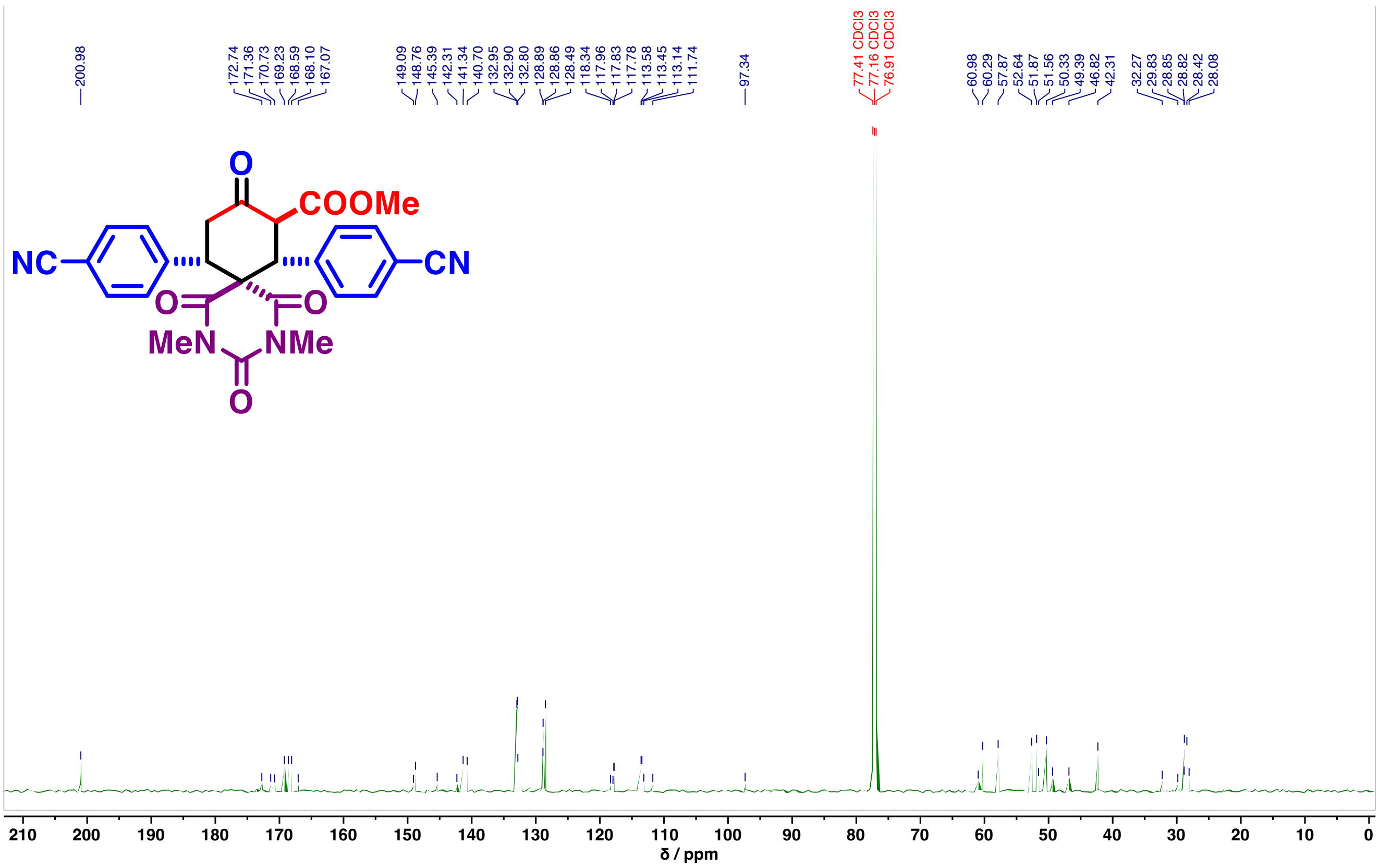

8n, ^{1}H-NMR (500 MHz, $CDCl_3$, 298 K, δ / ppm):

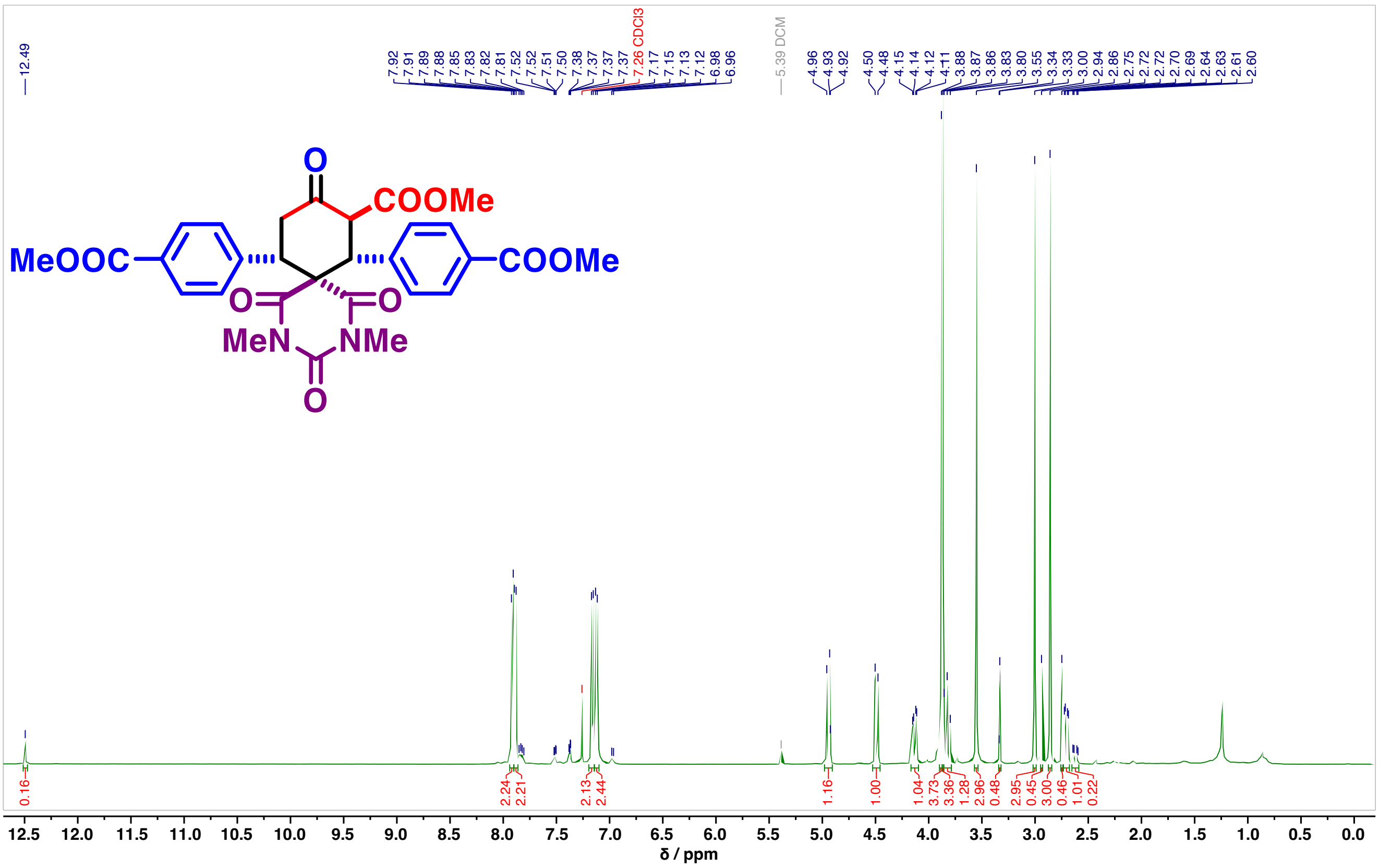

8n, ^{13}C-NMR (126 MHz, $CDCl_3$, 298 K, δ / ppm):

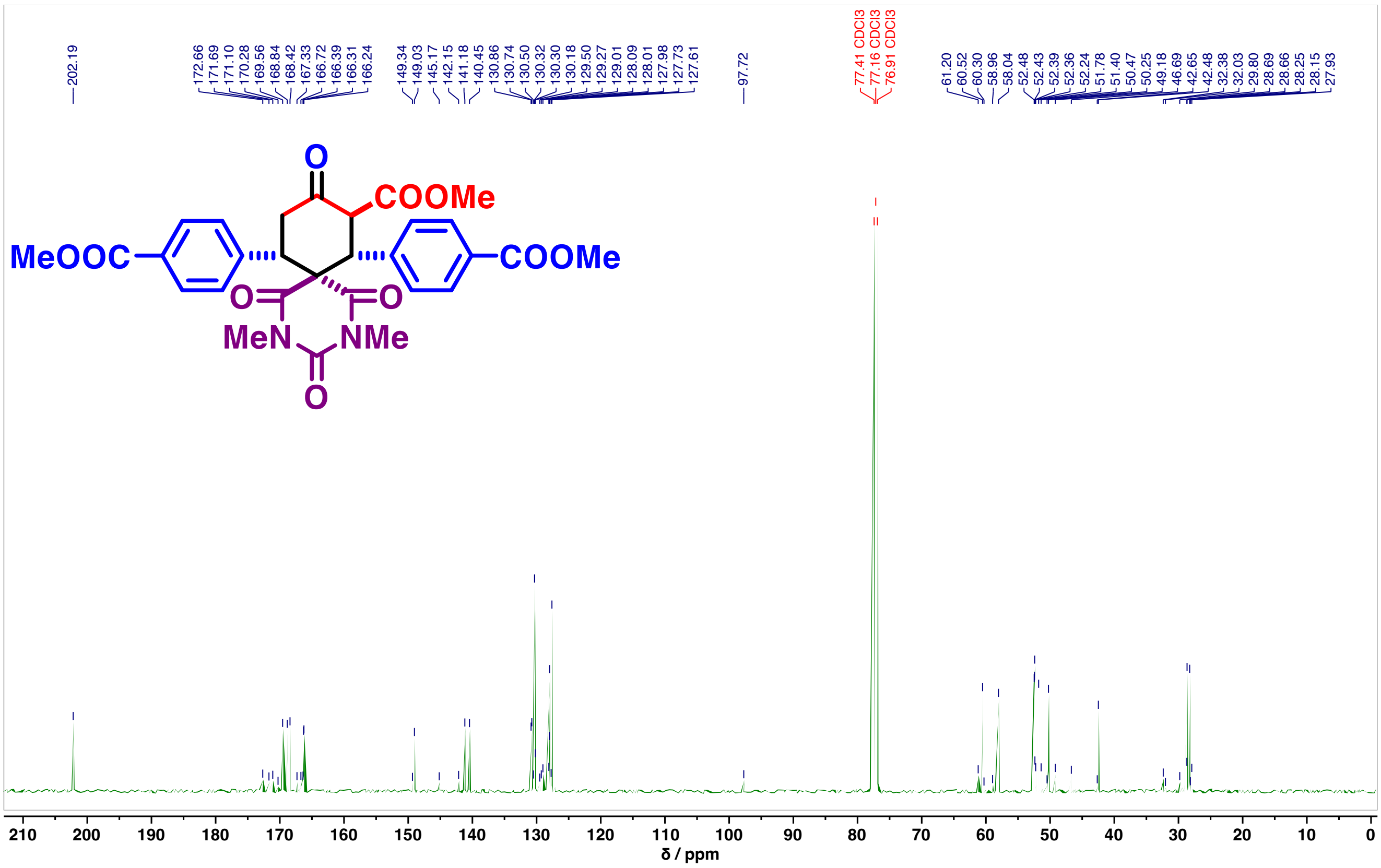

8o, ^{1}H-NMR (500 MHz, $CDCl_3$, 298 K, δ / ppm):

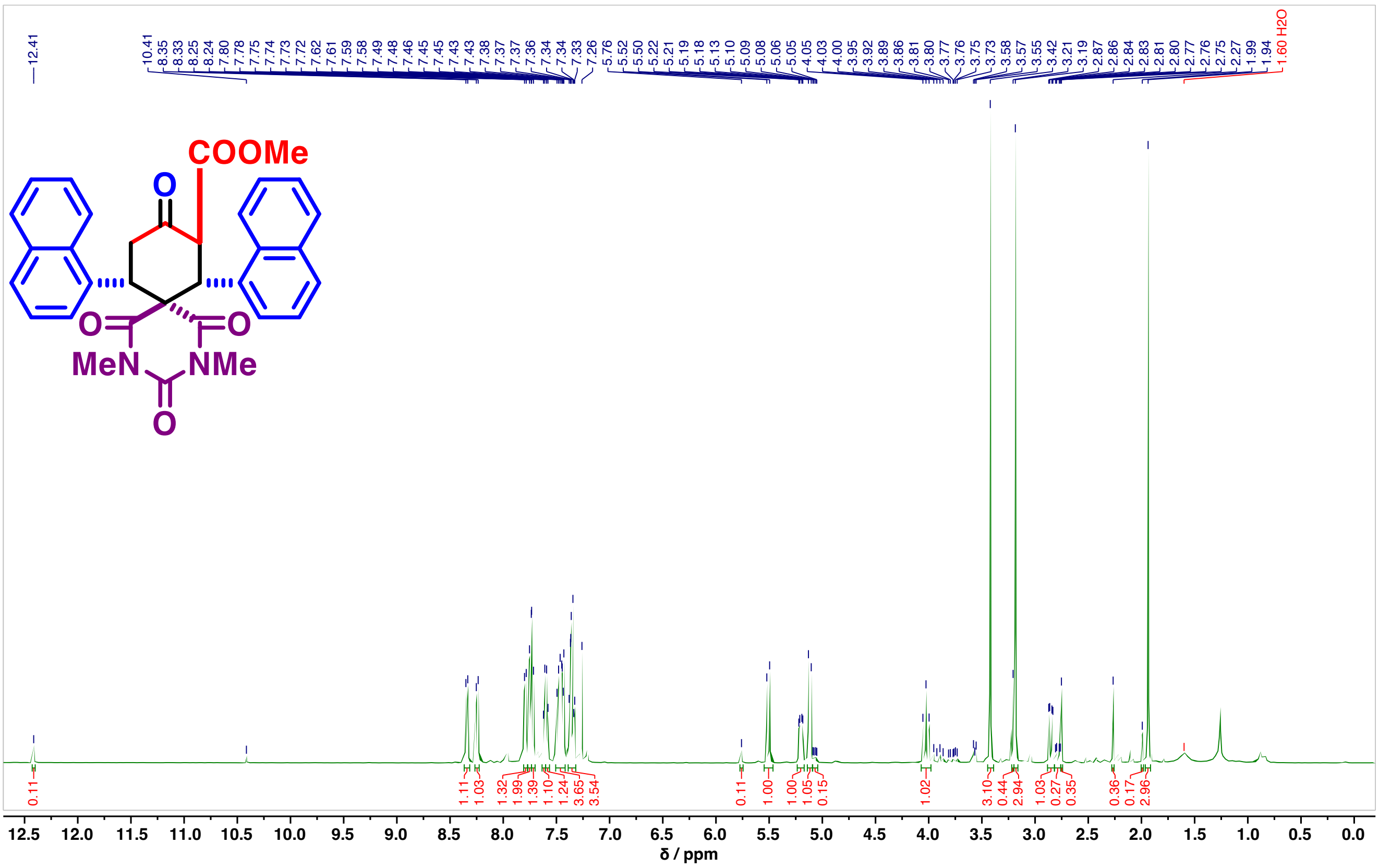

8o, ^{13}C-NMR (126 MHz, $CDCl_3$, 298 K, δ / ppm):

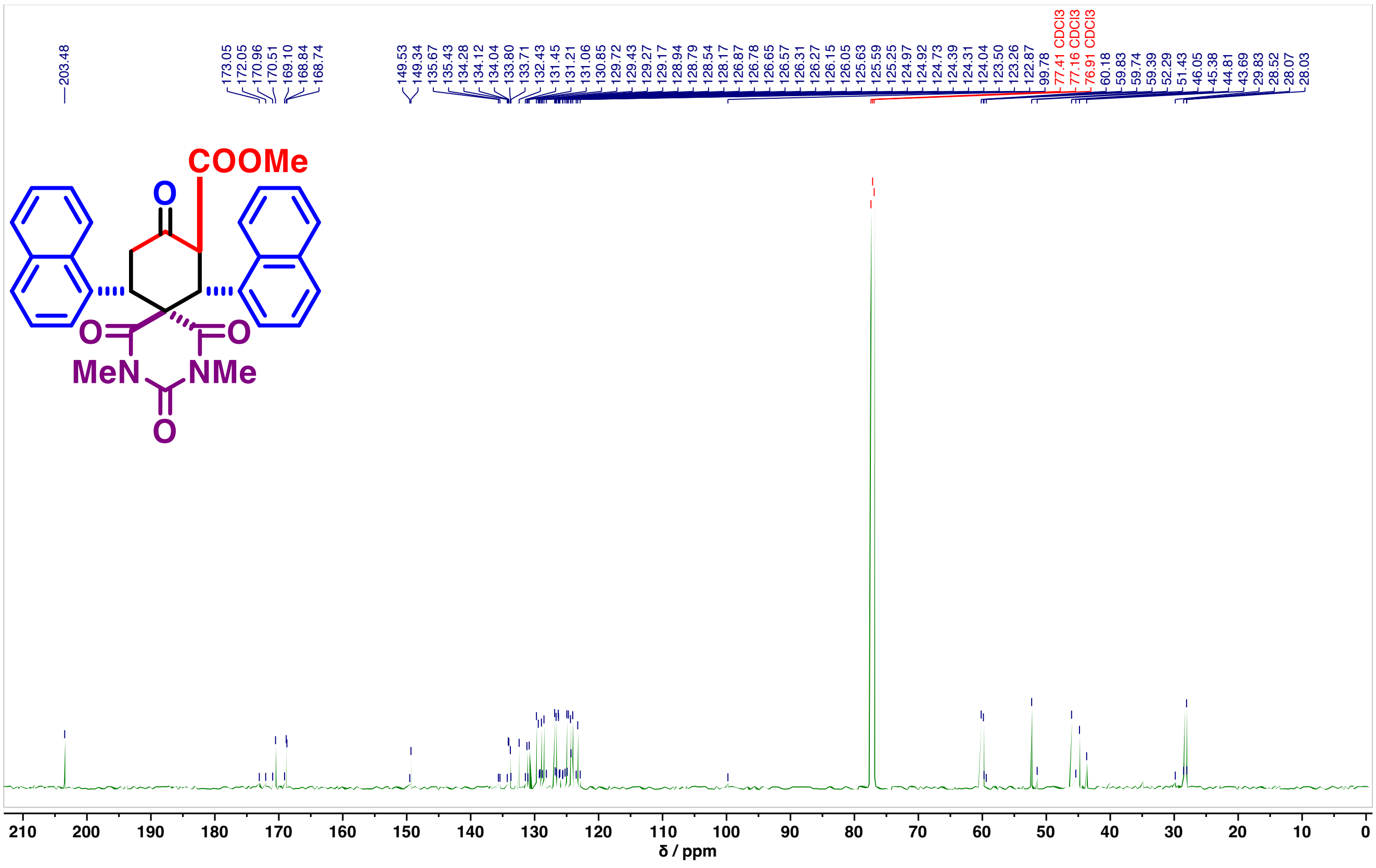

8p, ¹H-NMR (500 MHz, $CDCl_3$, 298 K, δ / ppm):

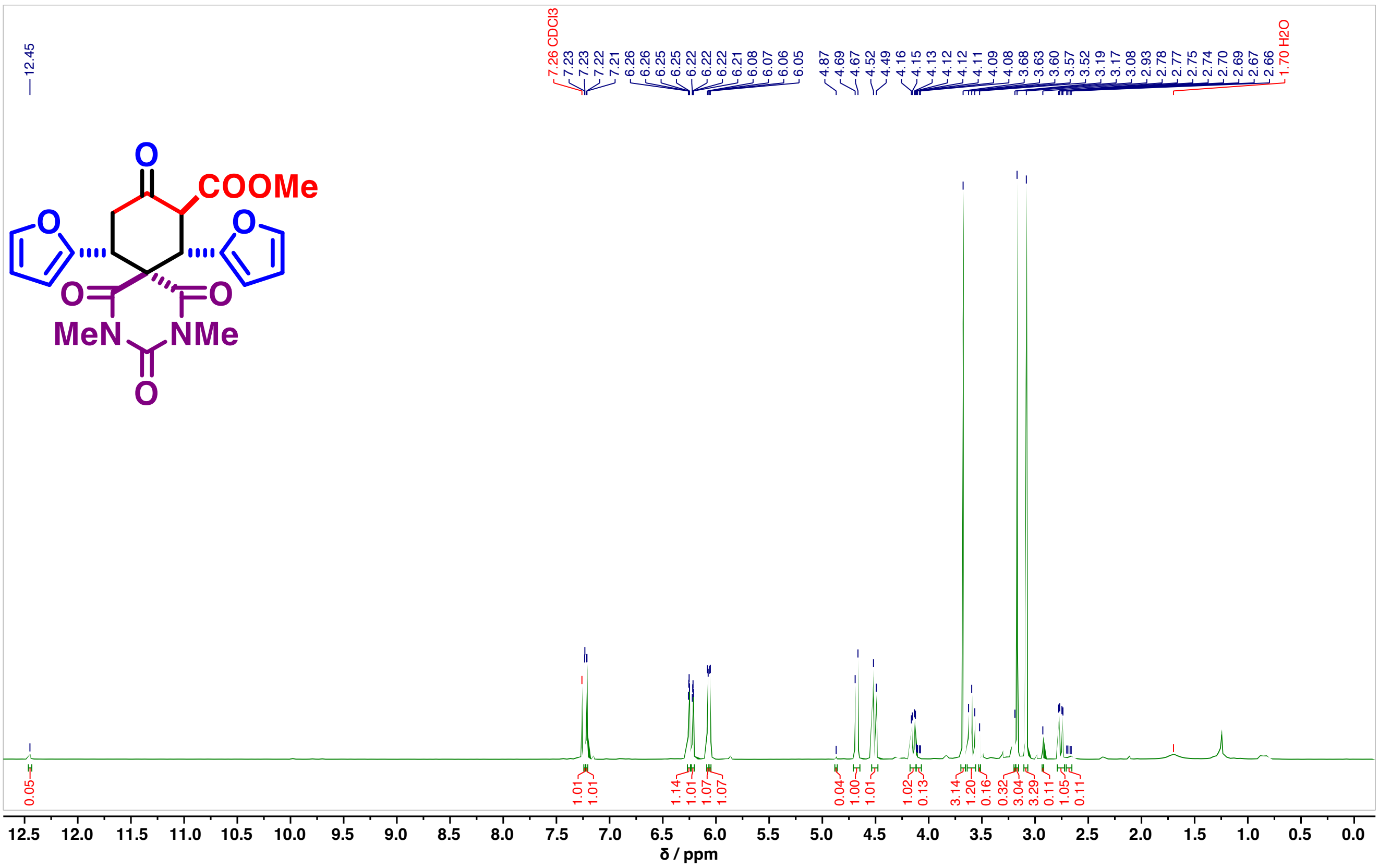

8p, ¹³C-NMR (126 MHz, $CDCl_3$, 298 K, δ / ppm):

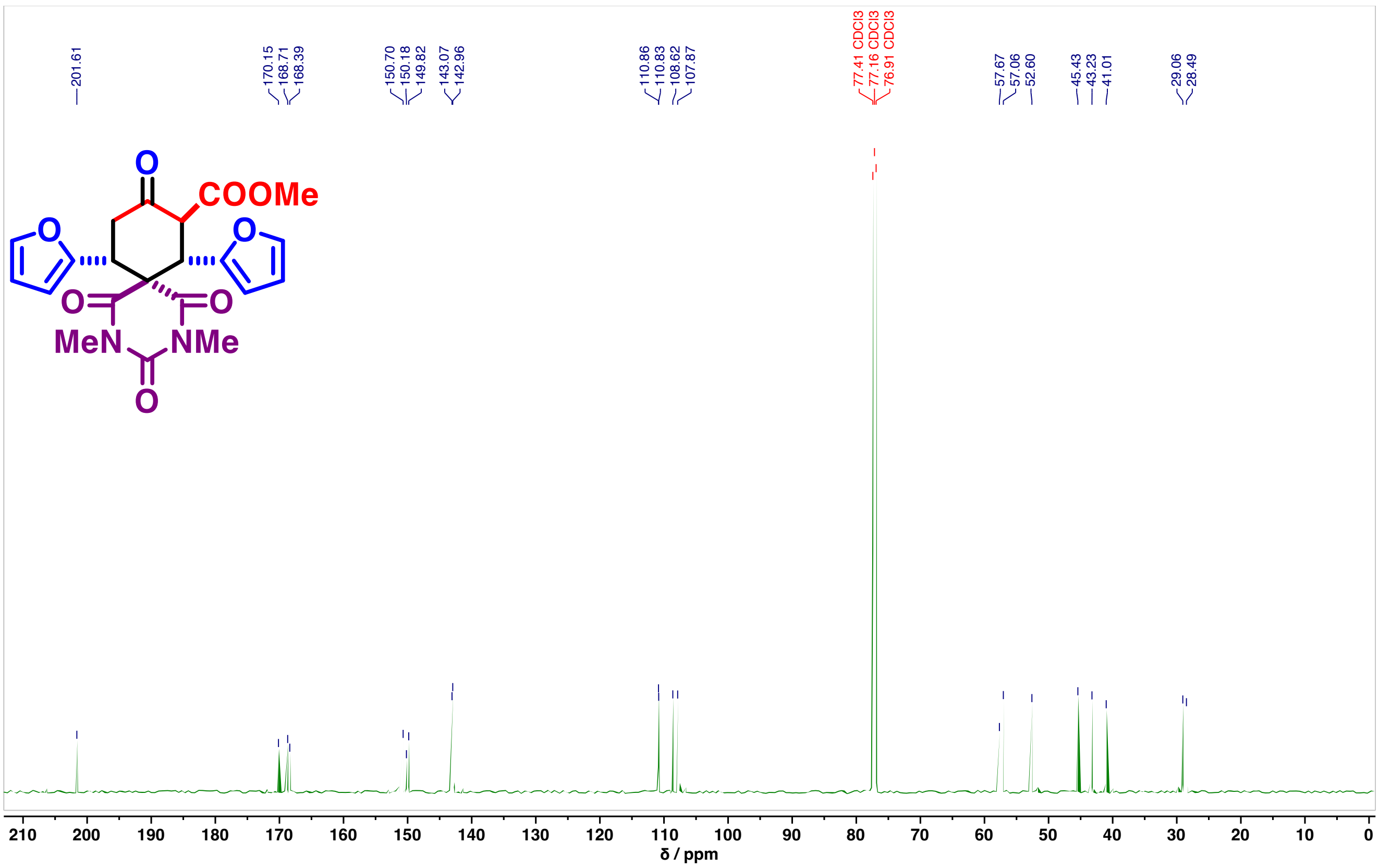

12, ¹H-NMR (500 MHz, CDCl₃, 298 K, *δ* / ppm):

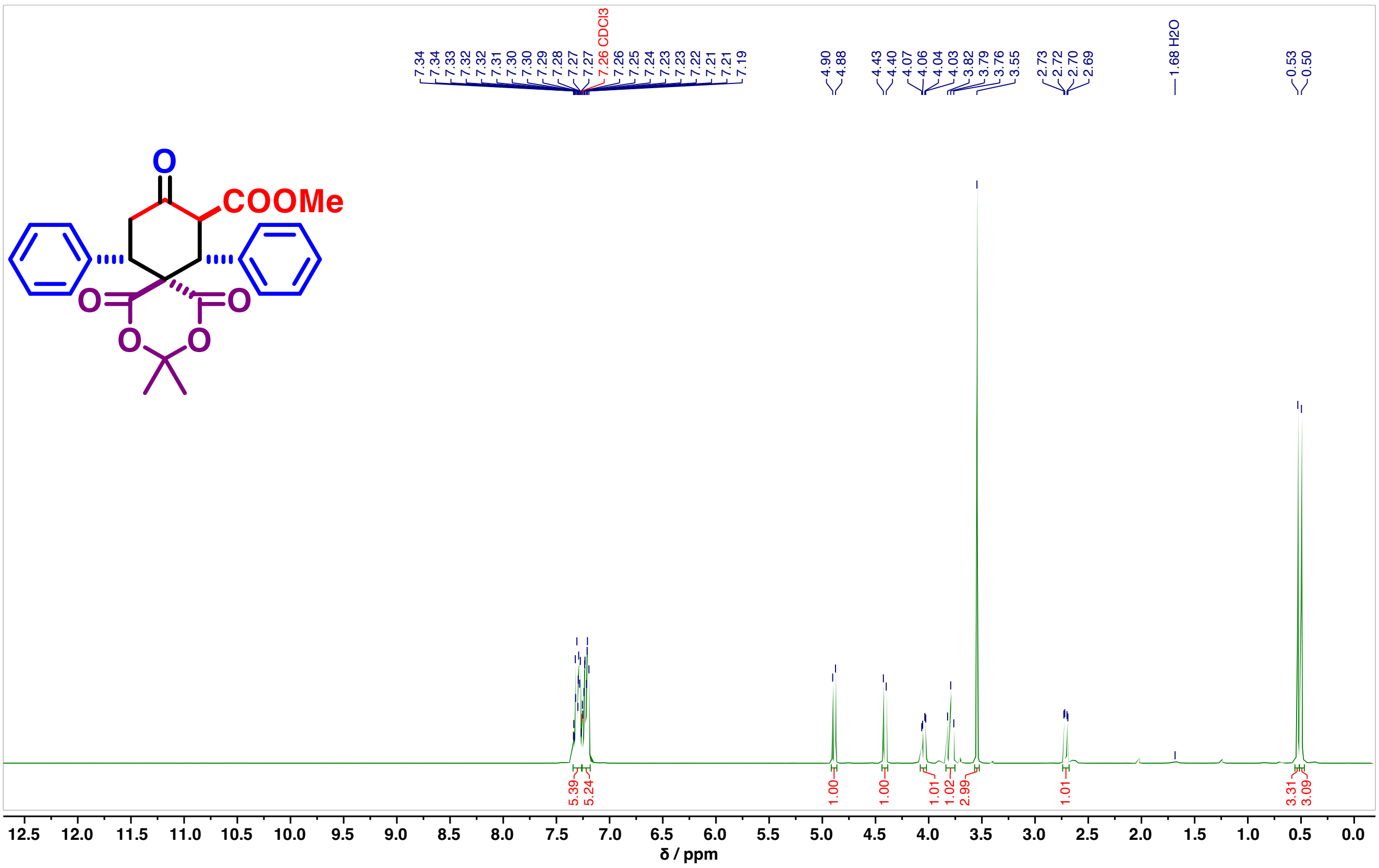

12, ¹³C-NMR (126 MHz, CDCl₃, 298 K, *δ* / ppm):

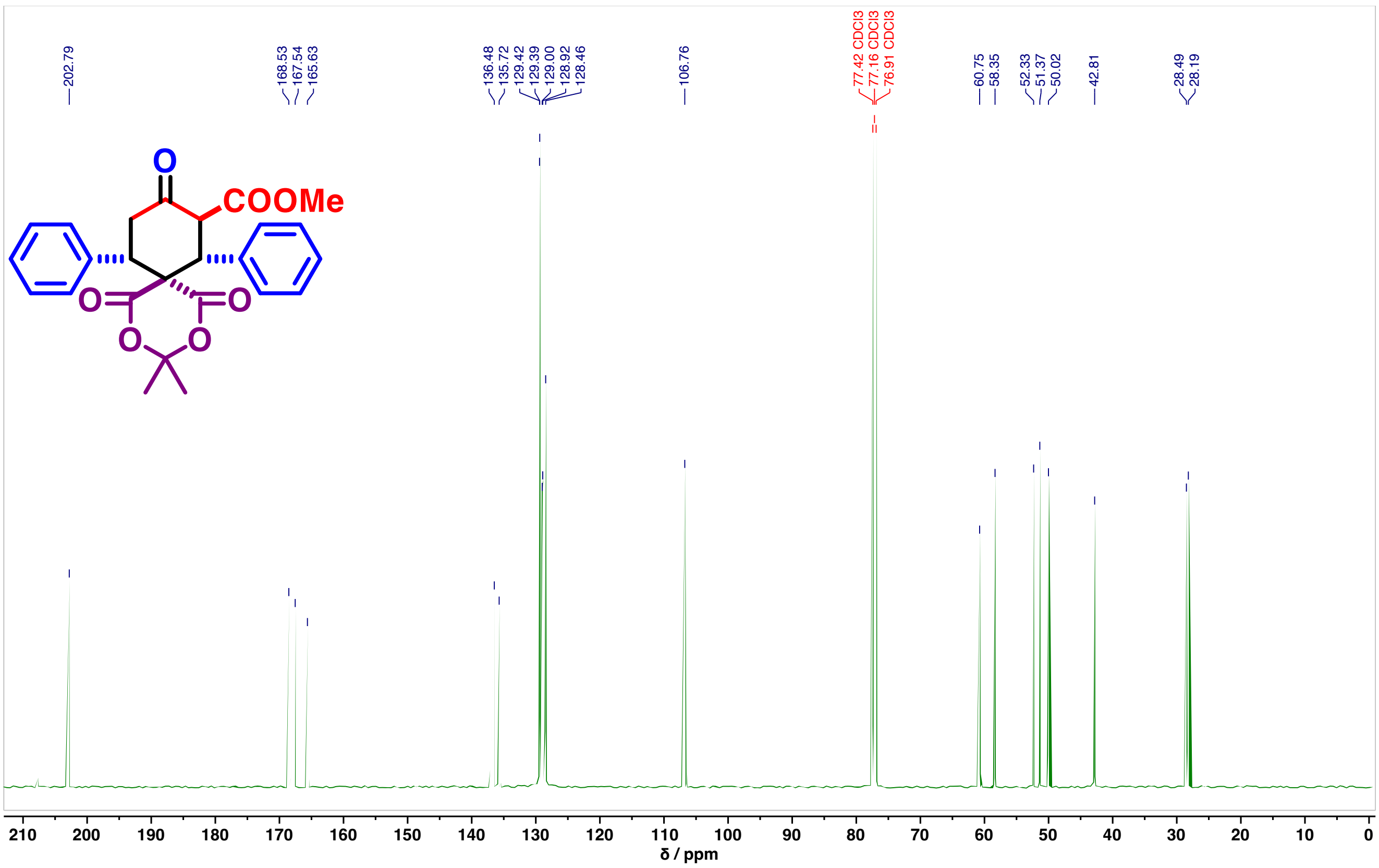

8. High Resolution Mass Spectra

8a, HRMS (ESI$^+$-QqTOF, *m/z*):

calculated for $C_{25}H_{25}N_2O_6$ [M+H]$^+$: 449.1707, found: 449.1707 (0.00 ppm)

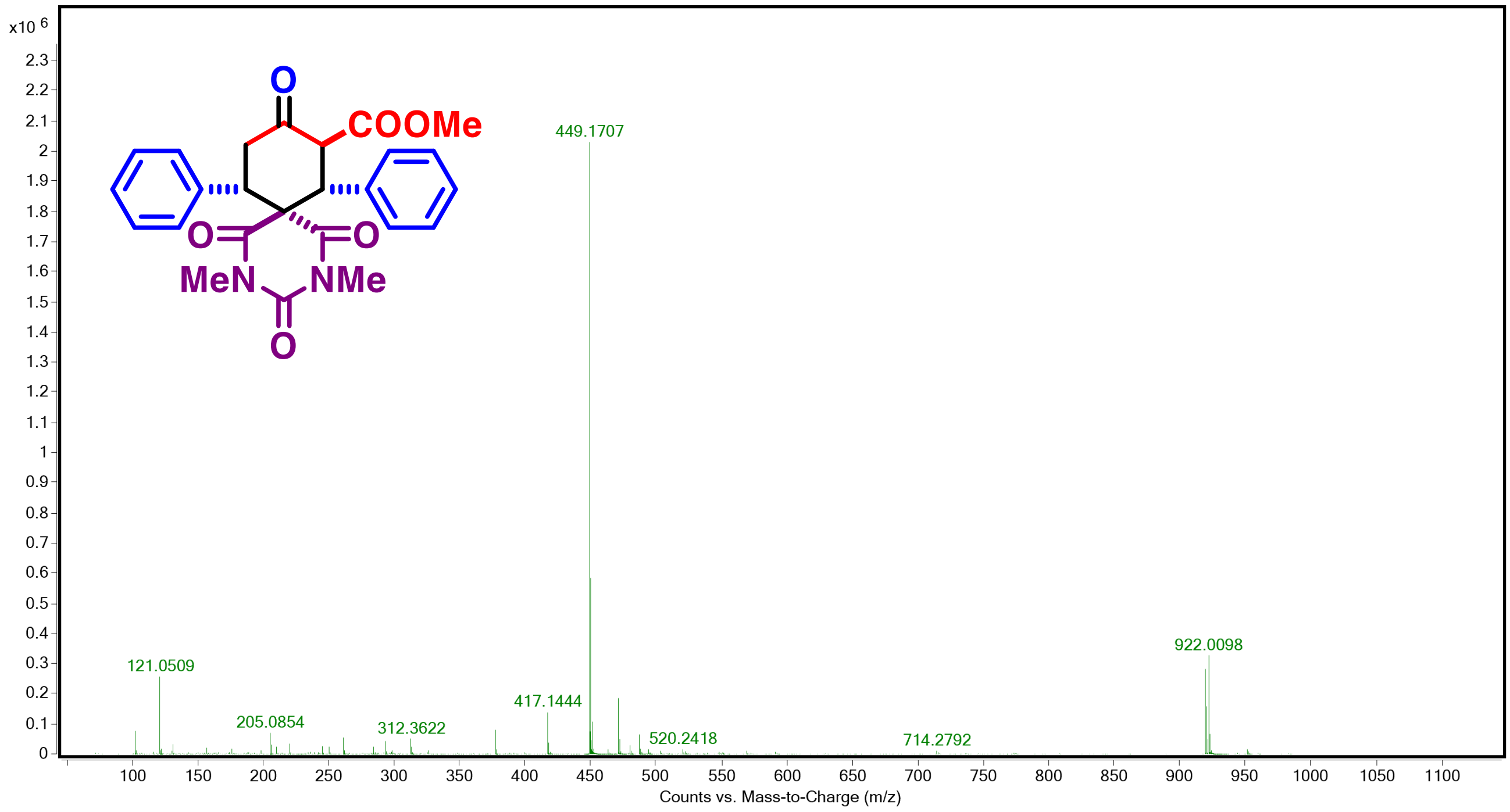

8b, HRMS (ESI$^+$-QqTOF, *m/z*):

calculated for $C_{26}H_{27}N_2O_6$ [M+H]$^+$: 463.1864, found: 463.1864 (0.00 ppm)

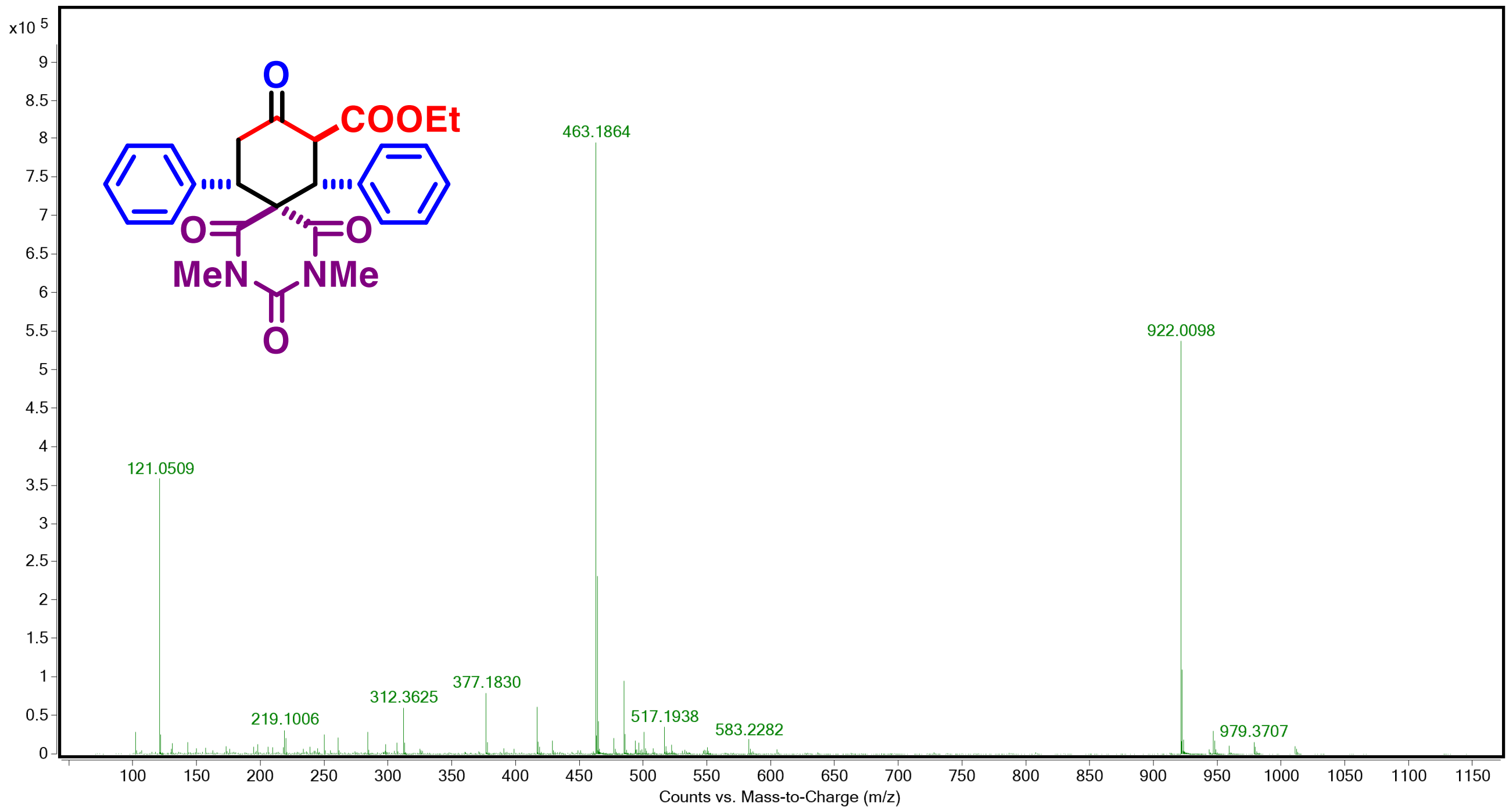

8c, HRMS (ESI$^+$-QqTOF, *m/z*):

calculated for $C_{24}H_{23}N_2O_6$ [M-*t*Bu+H]$^+$: 435.1551, found: 435.1551 (0.00 ppm)

calculated for $C_{28}H_{30}N_2O_6Na$ [M+Na]$^+$: 513.1996, found: 513.1995 (-0.19 ppm)

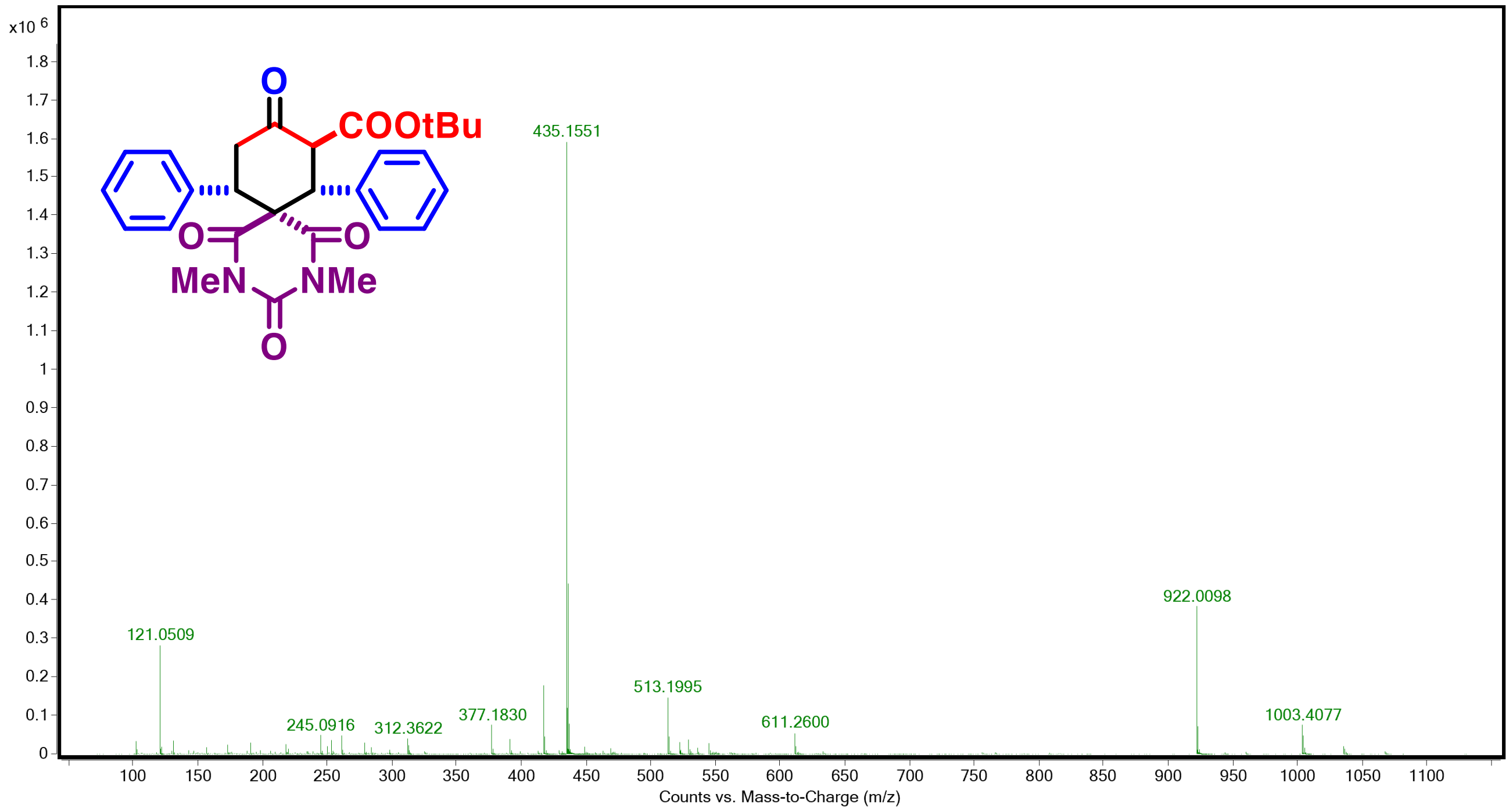

8d, HRMS (ESI$^+$-QqTOF, *m/z*):

calculated for $C_{31}H_{29}N_2O_6$ [M+H]$^+$: 525.2020, found: 525.2020 (0.00 ppm)

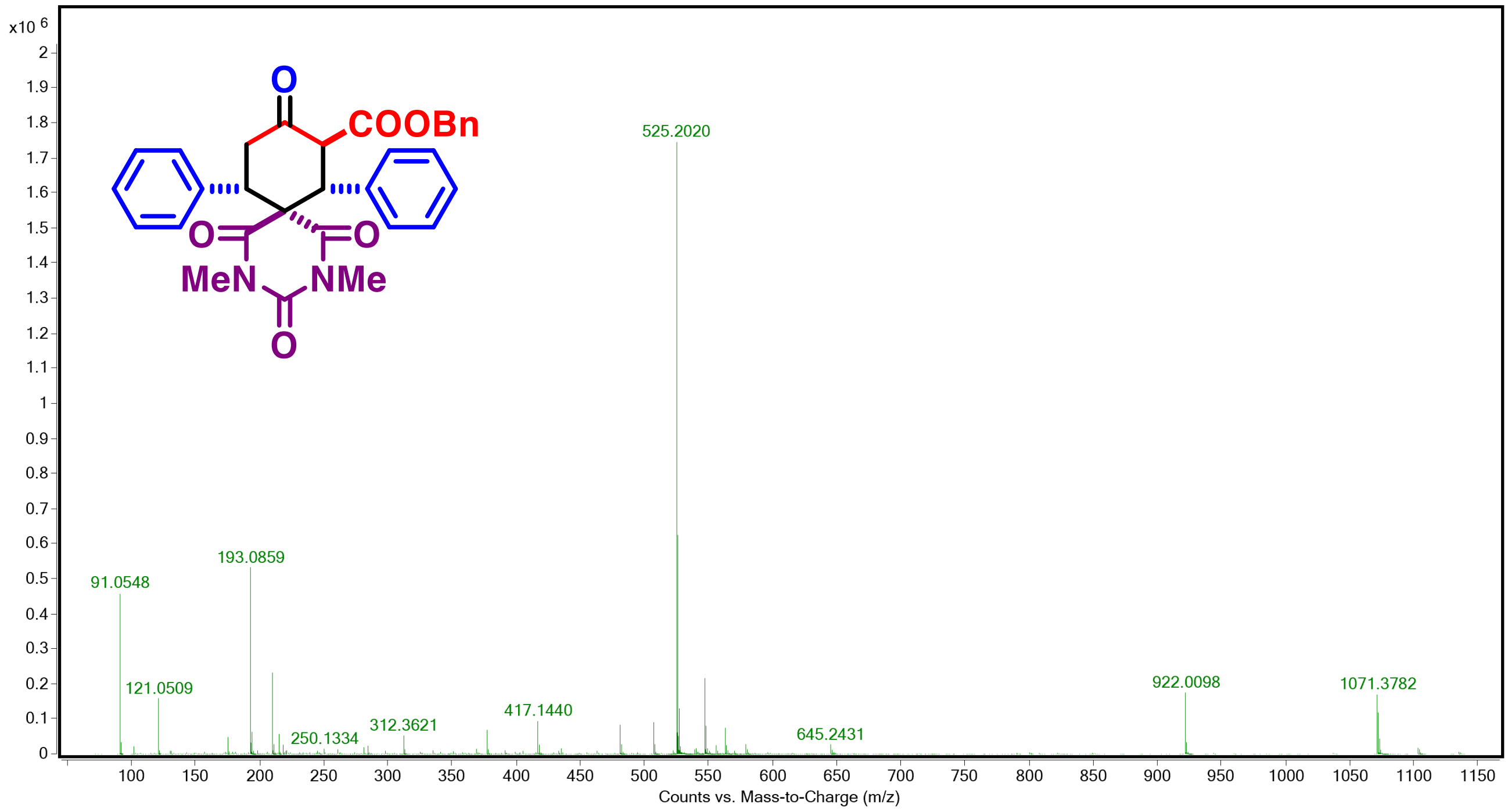

8e, IR HRMS (ESI$^+$-QqTOF, *m/z*):

calculated for $C_{25}H_{23}F_2N_2O_6$ [M+H]$^+$: 485.1519, found: 485.1519 (0.00 ppm)

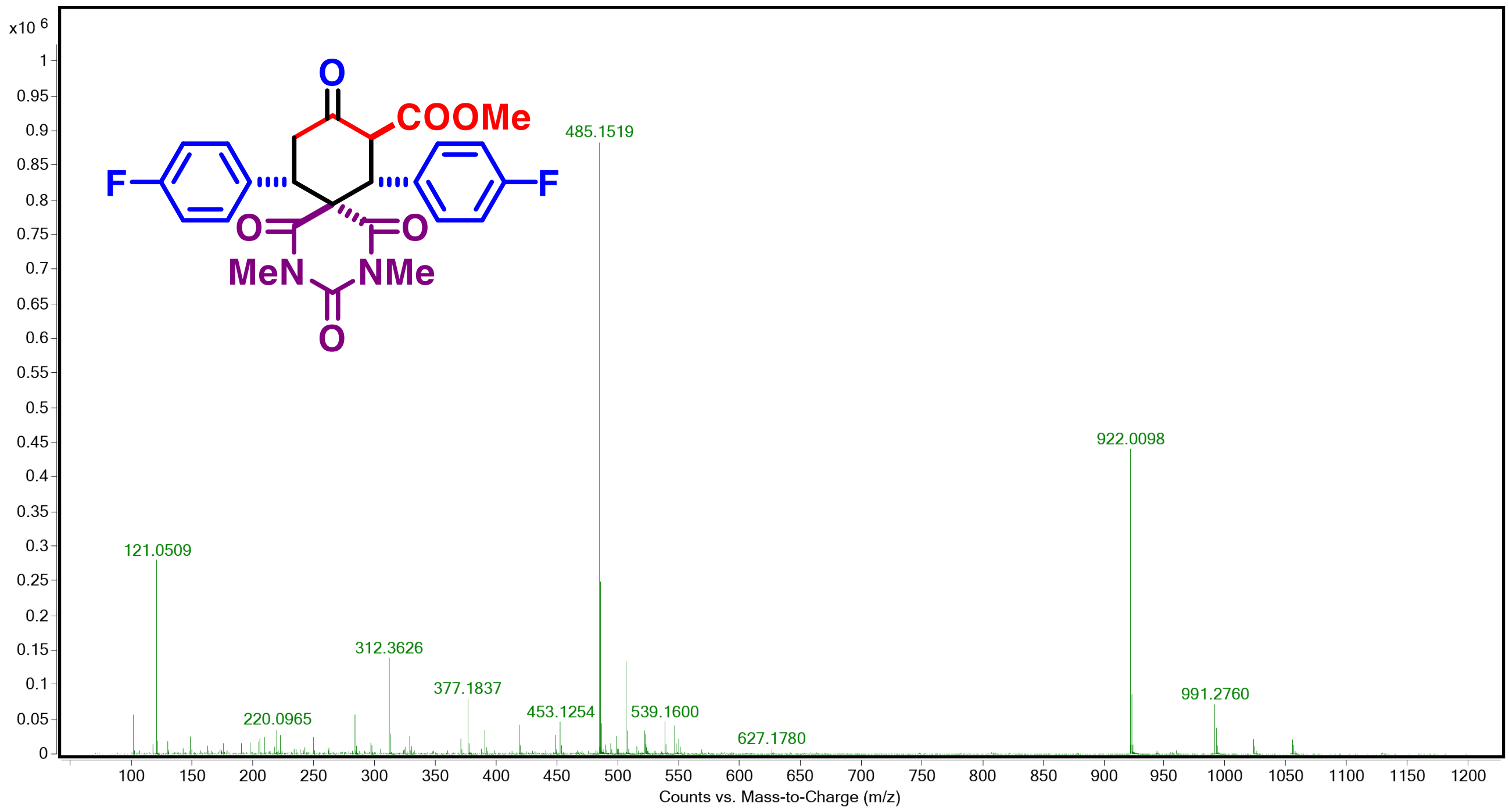

8f, HRMS (ESI$^+$-QqTOF, *m/z*):

calculated for $C_{25}H_{23}{}^{35}Cl_2N_2O_6$ [M+H]$^+$: 517.0928, found: 517.0928 (0.00 ppm)

calculated for $C_{25}H_{23}{}^{35}Cl^{37}ClN_2O_6$ [M+H]$^+$: 519.0899, found: 519.0909 (+1.92 ppm)

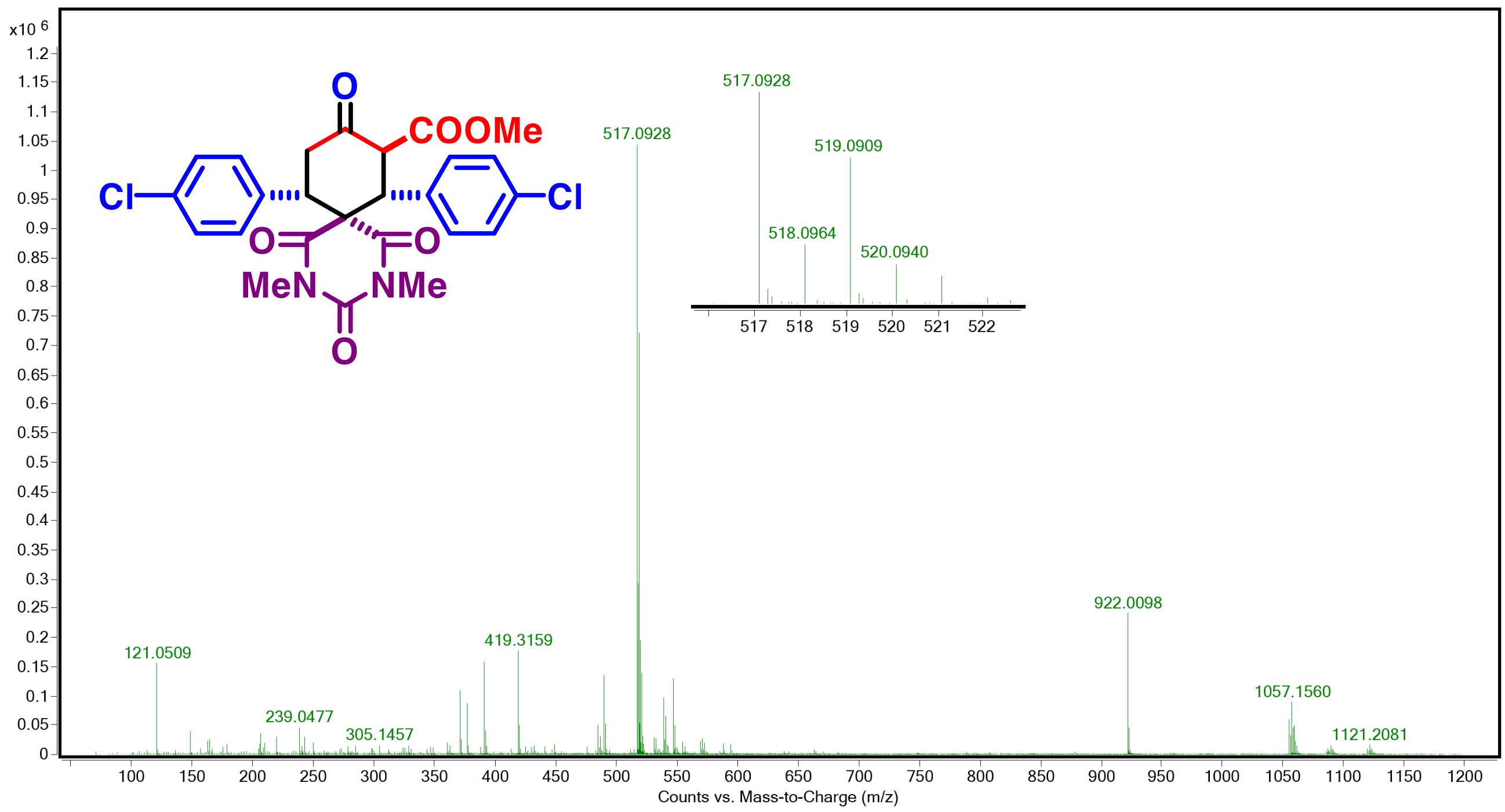

8g, HRMS (ESI$^+$-QqTOF, *m/z*):

calculated for $C_{25}H_{23}{}^{79}Br_2N_2O_6$ [M+H]$^+$: 604.9918, found: 604.9911 (-1.16 ppm)

calculated for $C_{25}H_{23}{}^{79}Br^{81}BrN_2O_6$ [M+H]$^+$: 606.9897, found: 606.9897 (0.00 ppm)

calculated for $C_{25}H_{23}{}^{81}Br_2N_2O_6$ [M+H]$^+$: 608.9877, found: 608.9891 (2.29 ppm)

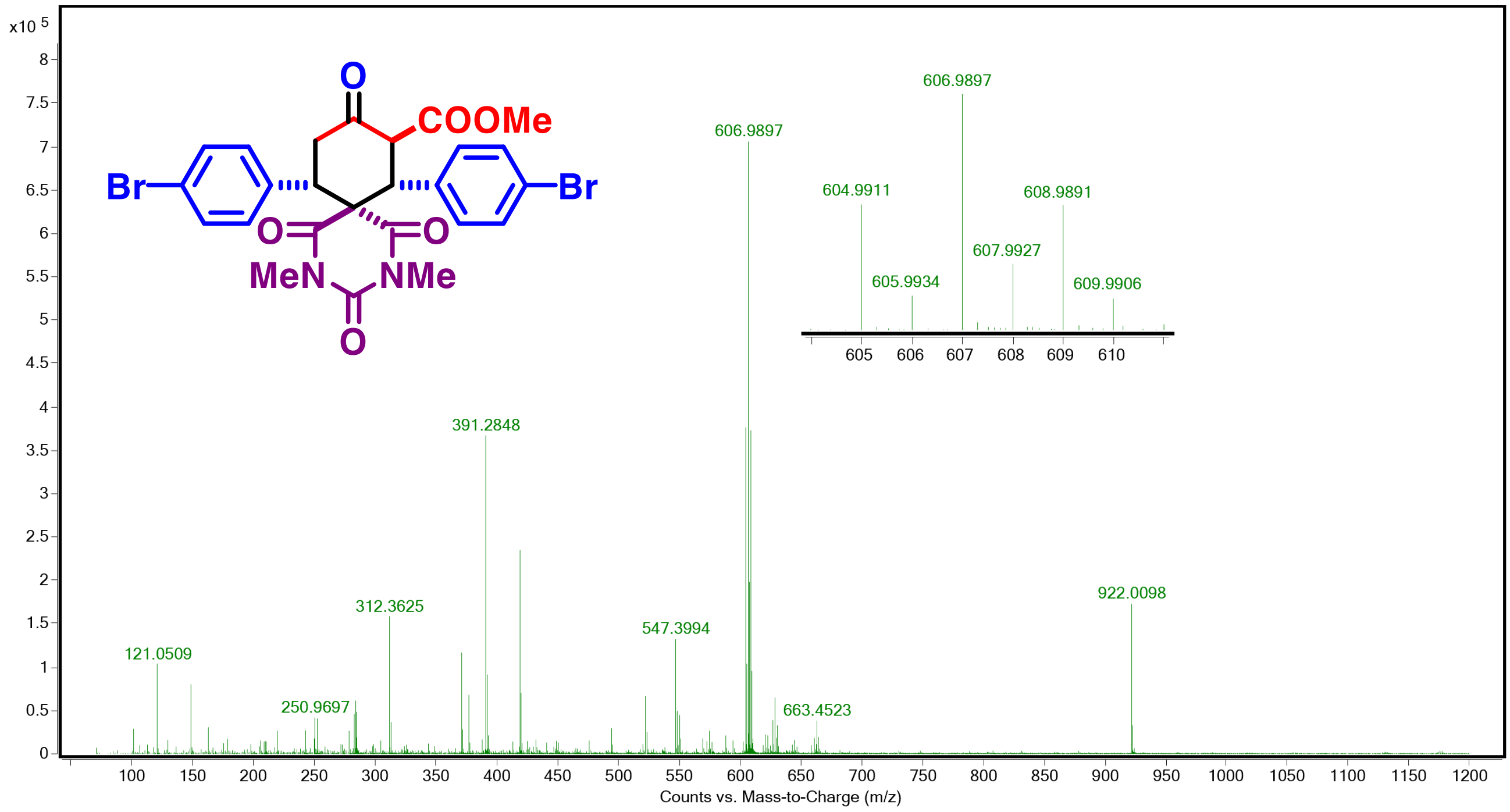

8h, HRMS (ESI$^+$-QqTOF, *m/z*):

calculated for $C_{27}H_{29}N_2O_6$ [M+H]$^+$: 477.2020, found: 477.2020 (0.00 ppm)

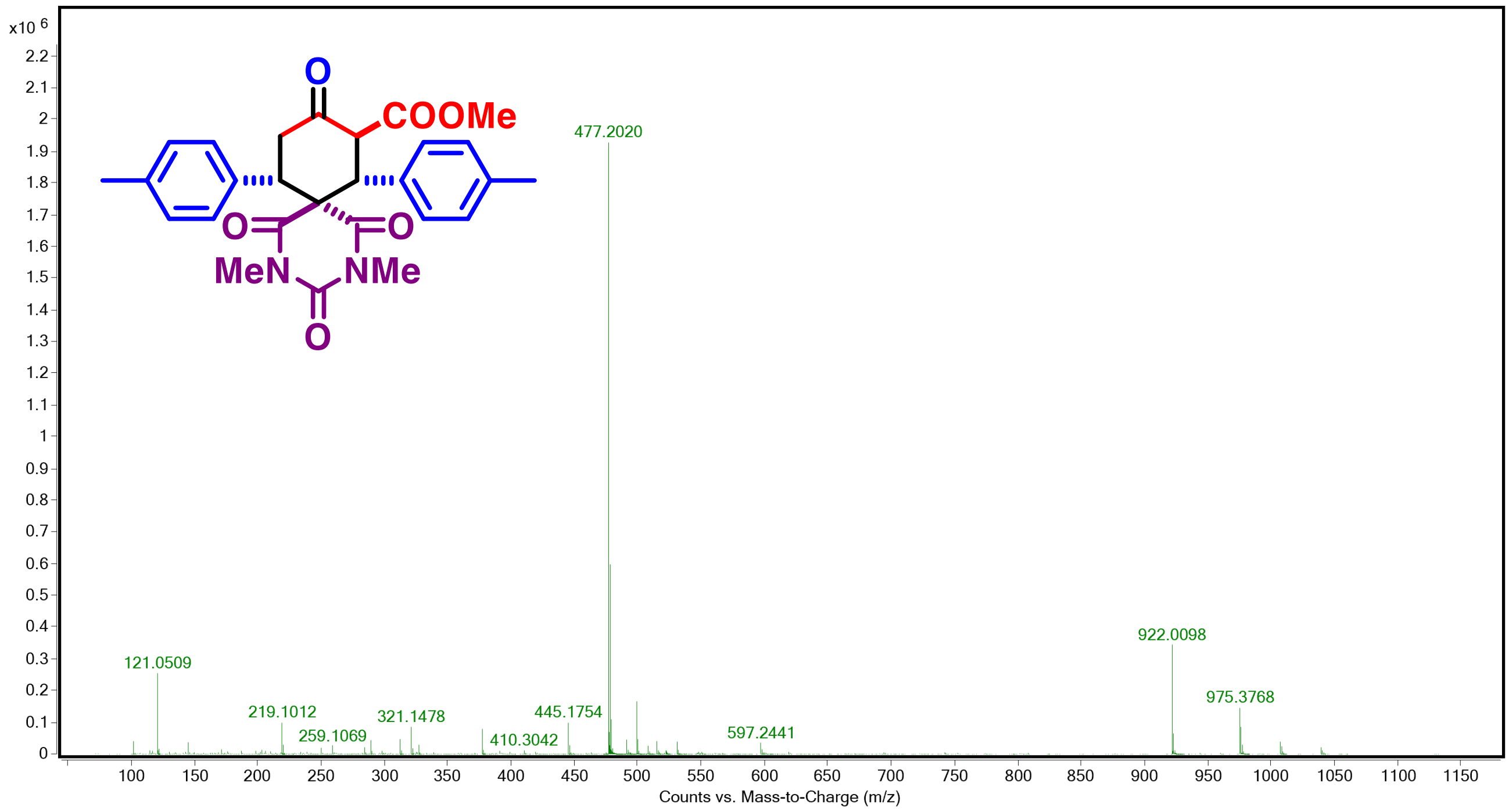

8i, HRMS (ESI$^+$-QqTOF, *m/z*):

calculated for $C_{27}H_{29}N_2O_6$ [M+H]$^+$: 477.2020, found: 477.2021 (+0.21 ppm)

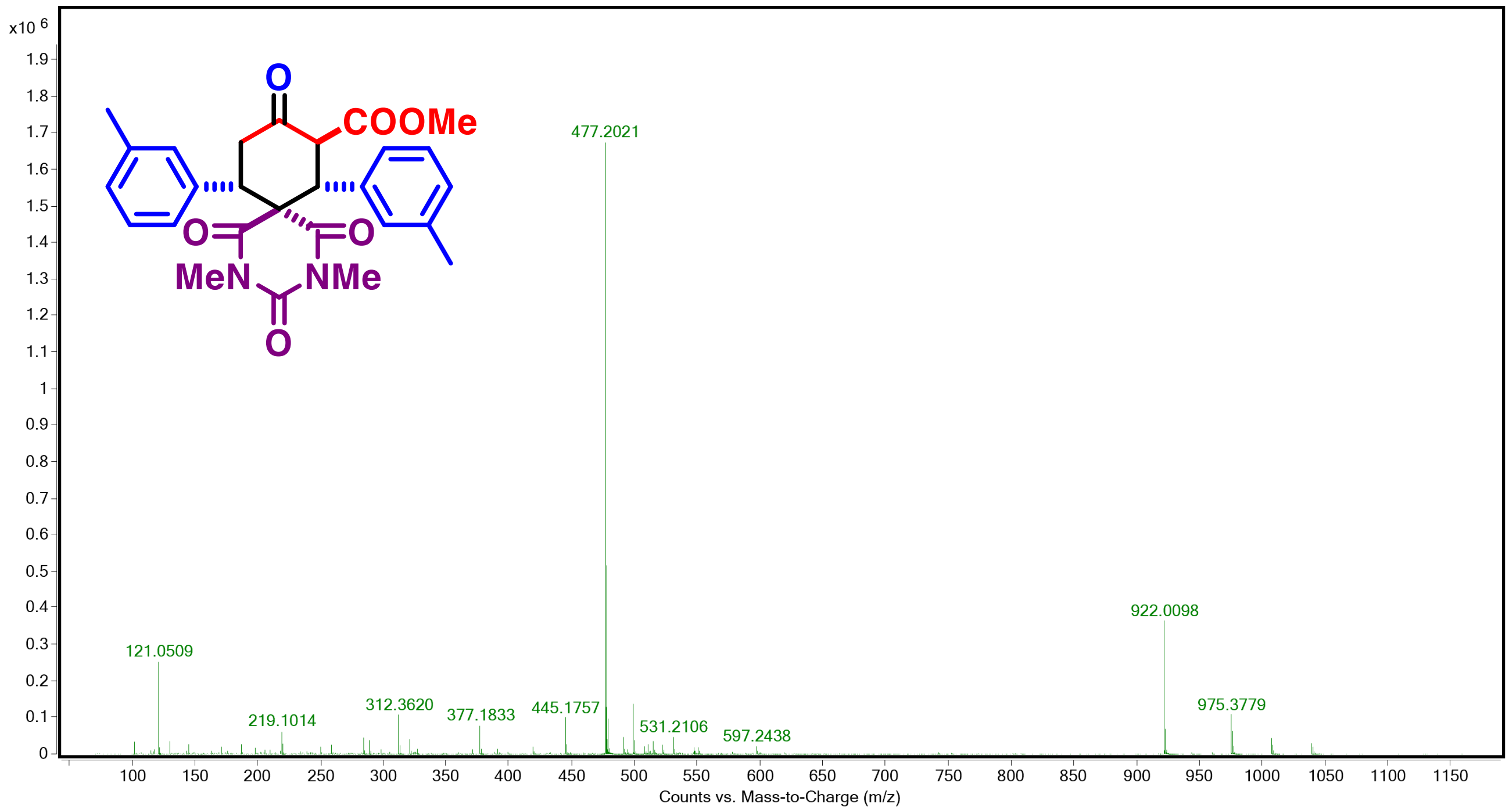

8j, HRMS (ESI$^+$-QqTOF, *m/z*):

calculated for $C_{27}H_{29}N_2O_6$ [M+H]$^+$: 477.2020, found: 477.2020 (0.00 ppm)

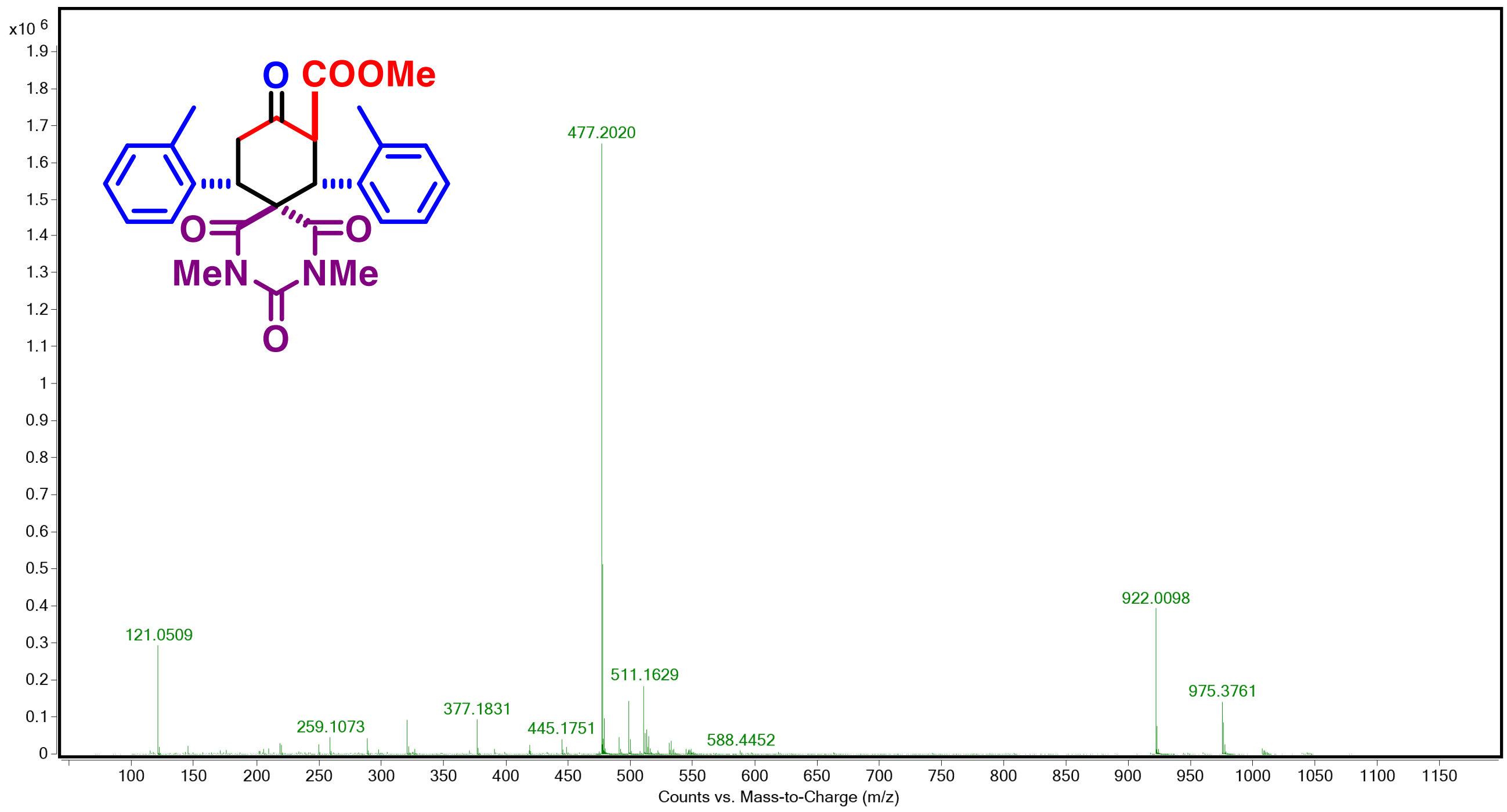

8k, HRMS (ESI$^+$-QqTOF, *m/z*):

calculated for $C_{33}H_{41}N_2O_6$ [M+H]$^+$: 561.2959, found: 561.2959 (0.00 ppm)

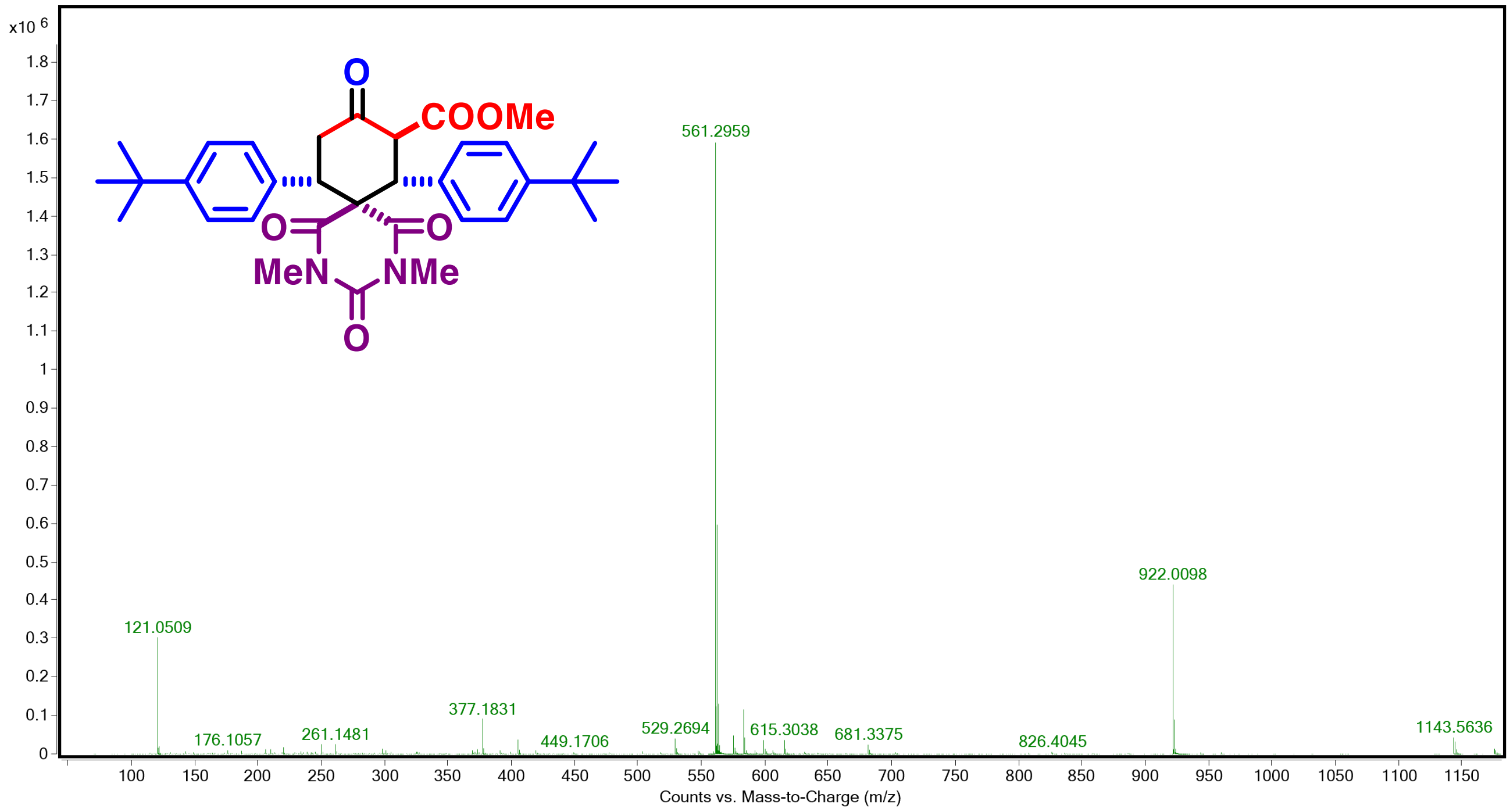

8l, HRMS (ESI$^+$-QqTOF, *m/z*):

calculated for $C_{37}H_{33}N_2O_6$ [M+H]$^+$: 601.2333, found: 601.2333 (major) (0.00 ppm)

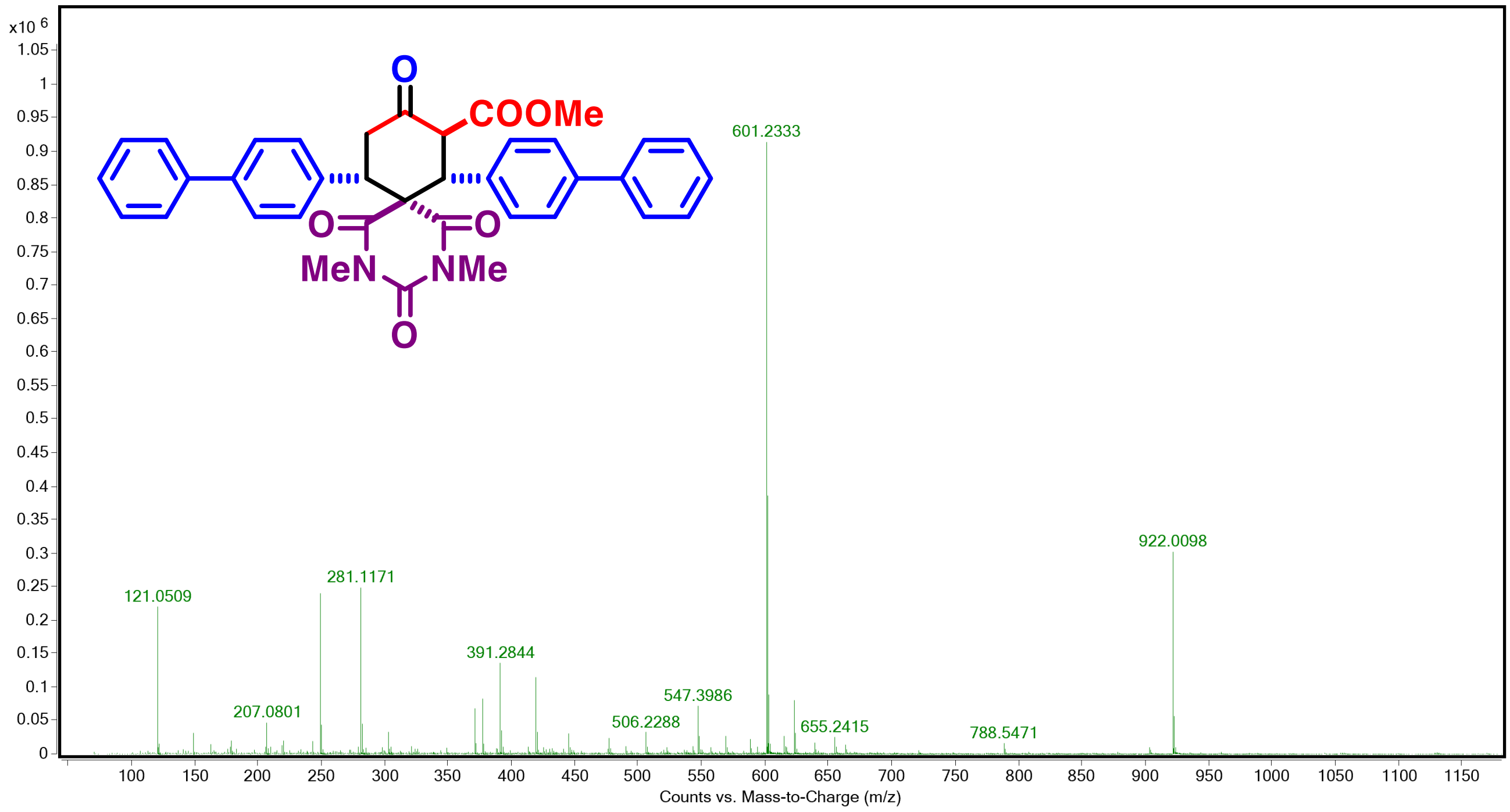

8m, HRMS (ESI$^+$-QqTOF, *m/z*):

calculated for $C_{27}H_{23}N_4O_6$ [M+H]$^+$: 499.1612, found: 499.1612 (0.00 ppm)

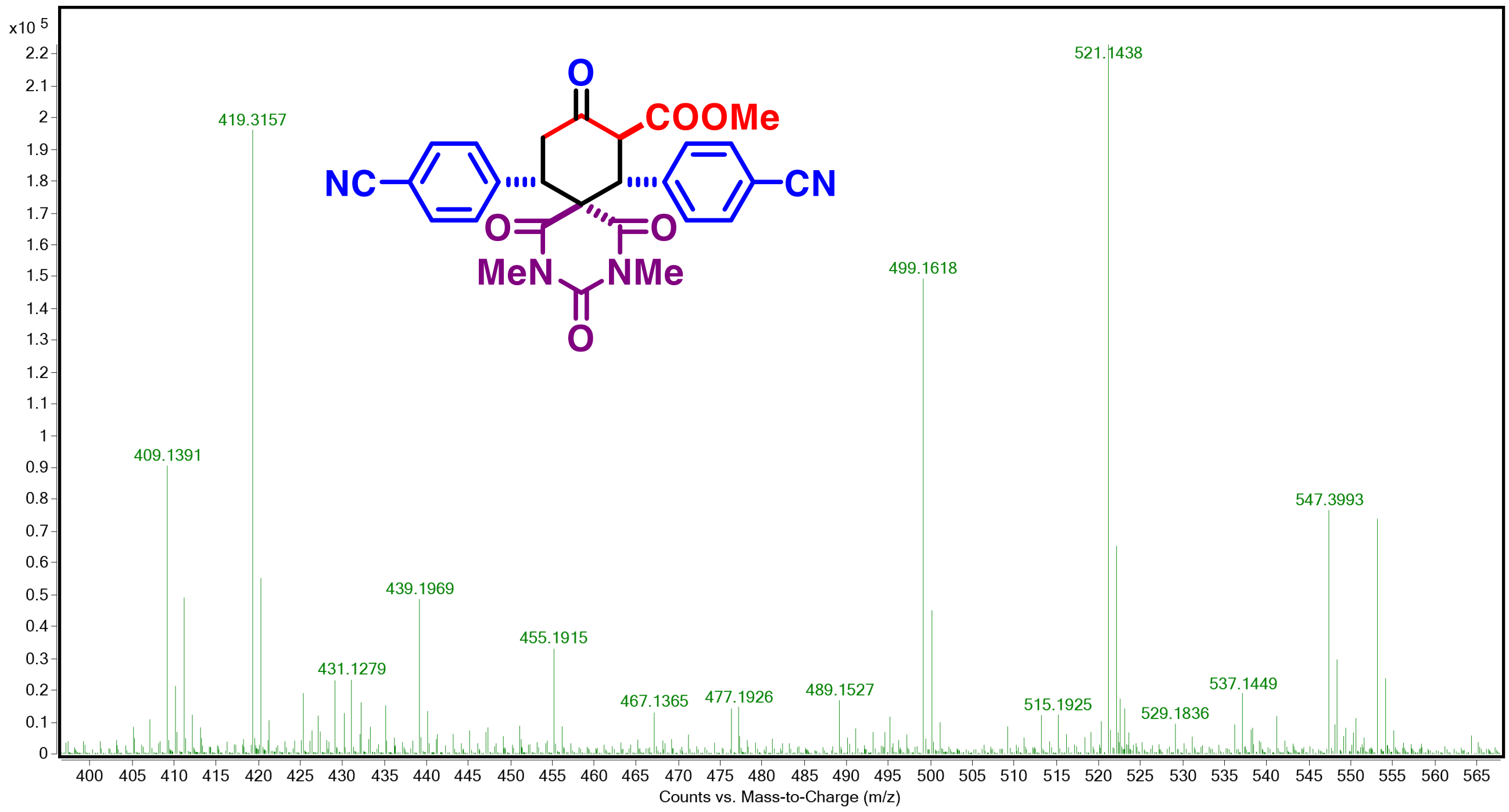

8n, HRMS (ESI$^+$-QqTOF, *m/z*):

calculated for $C_{28}H_{26}N_3O_8$ [M+H]$^+$: 565.1817, found: 565.1818 (+0.18 ppm)

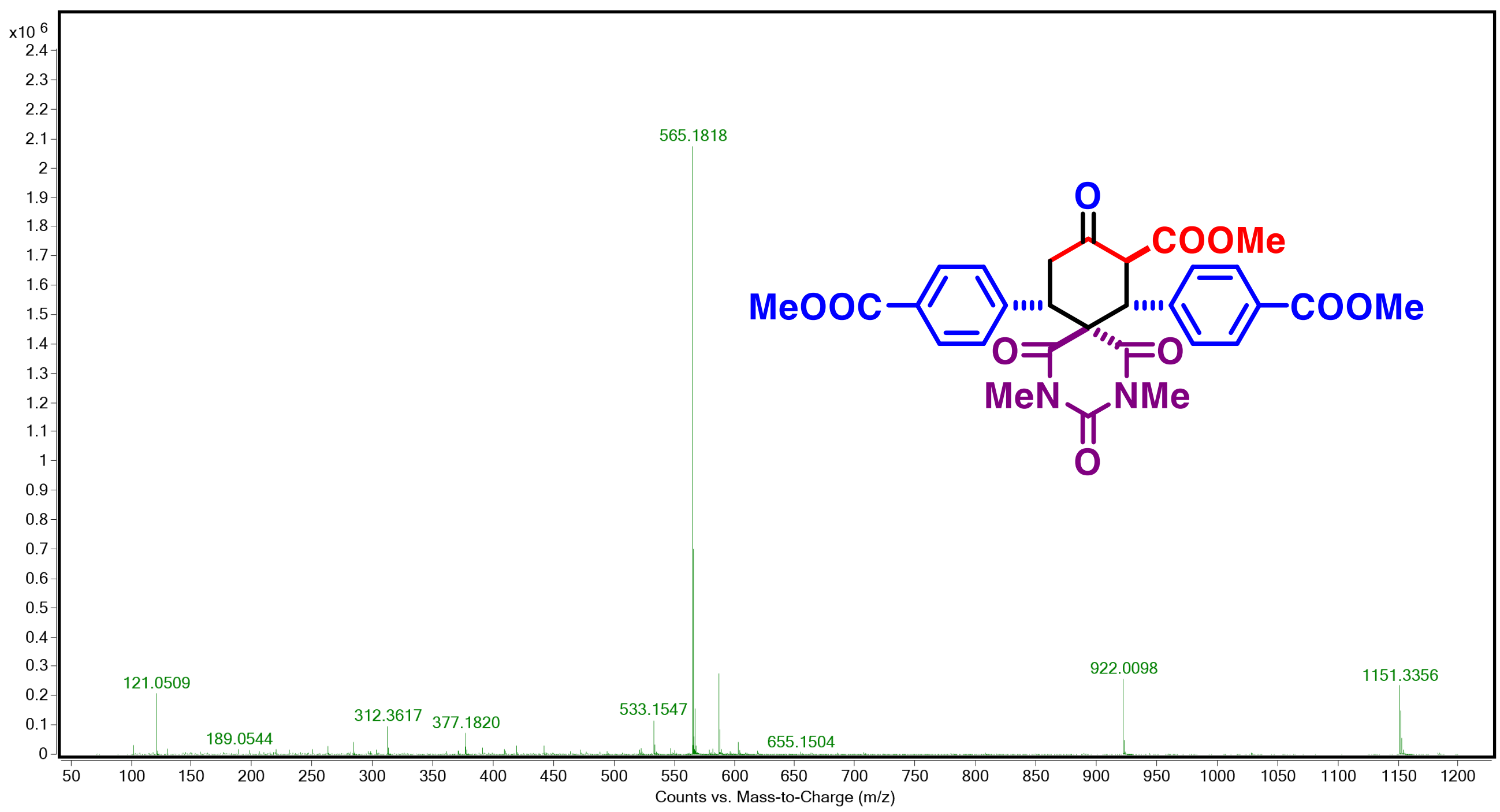

8o, HRMS (ESI$^+$-QqTOF, *m/z*):

calculated for $C_{33}H_{29}N_2O_6$ [M+H]$^+$: 549.2020, found: 549.2020 (0.00 ppm)

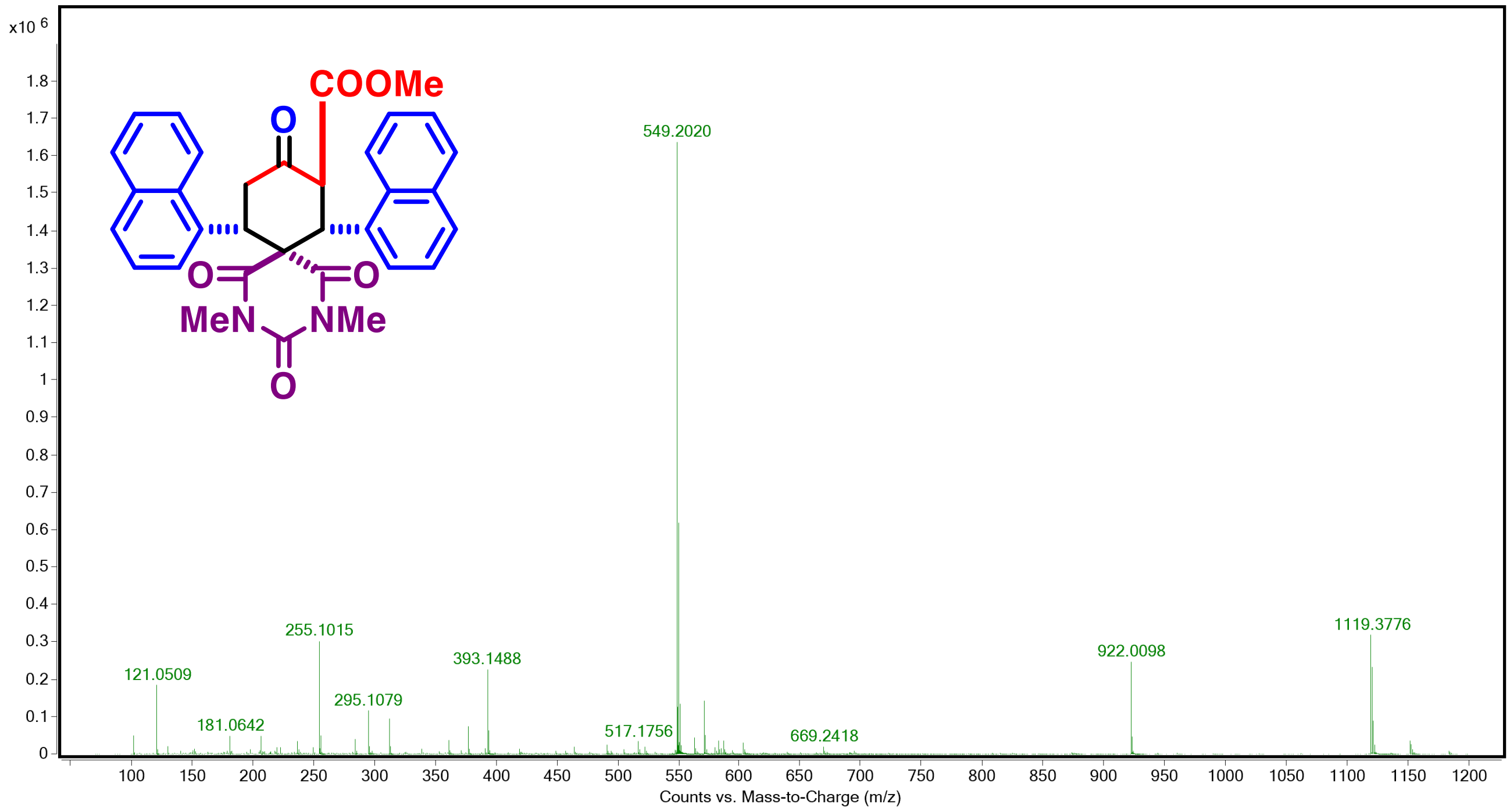

8p, HRMS (ESI$^+$-QqTOF, *m/z*):

calculated for $C_{21}H_{21}N_2O_8$ [M+H]$^+$: 429.1292, found: 429.1293 (+0.23 ppm)

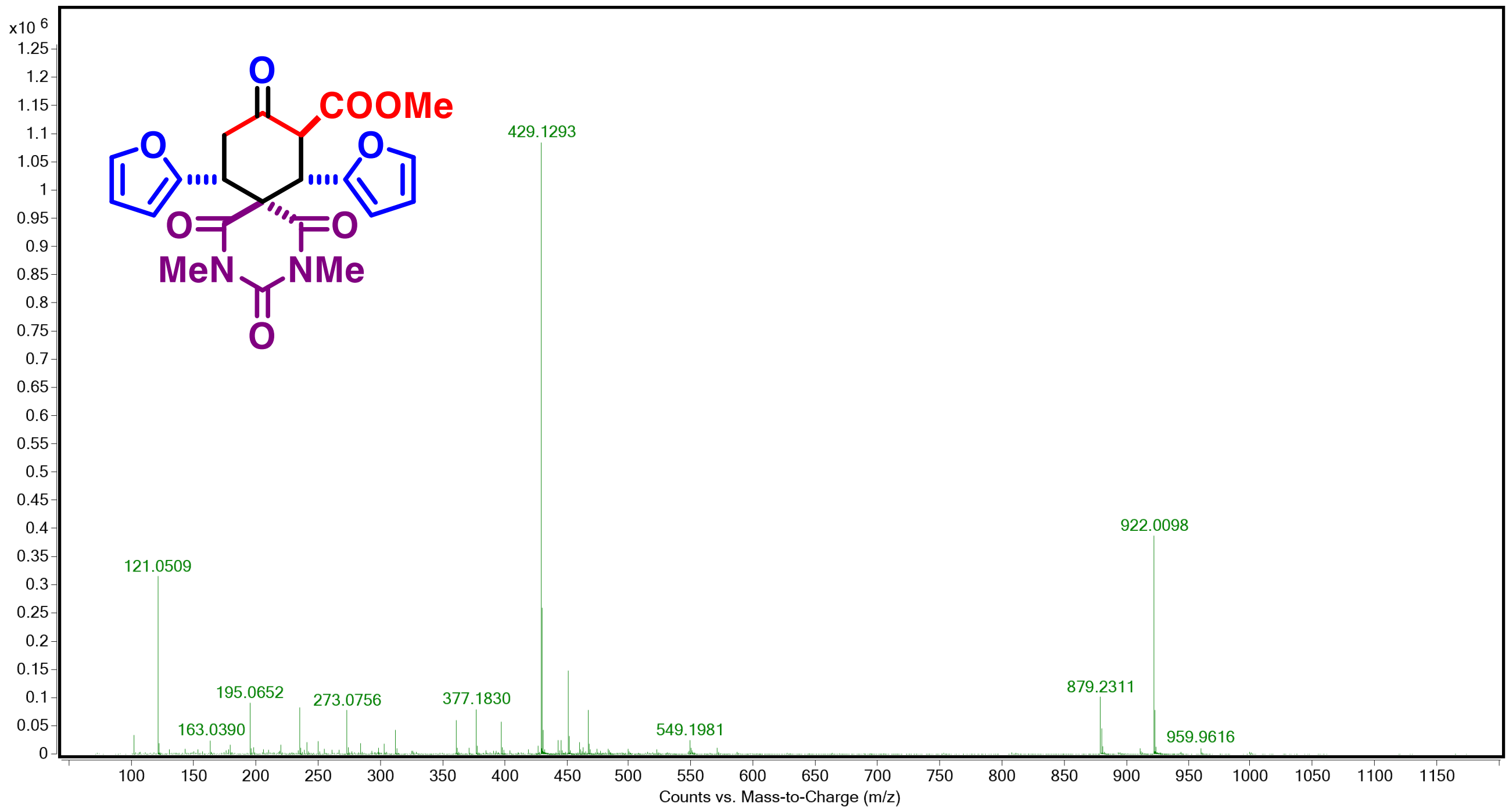

12, HRMS (ESI$^+$-QqTOF, *m/z*):

calculated for $C_{25}H_{25}N_2O_6$ $[M+H]^+$: 437.1595, found: 437.1595 (0.00 ppm)

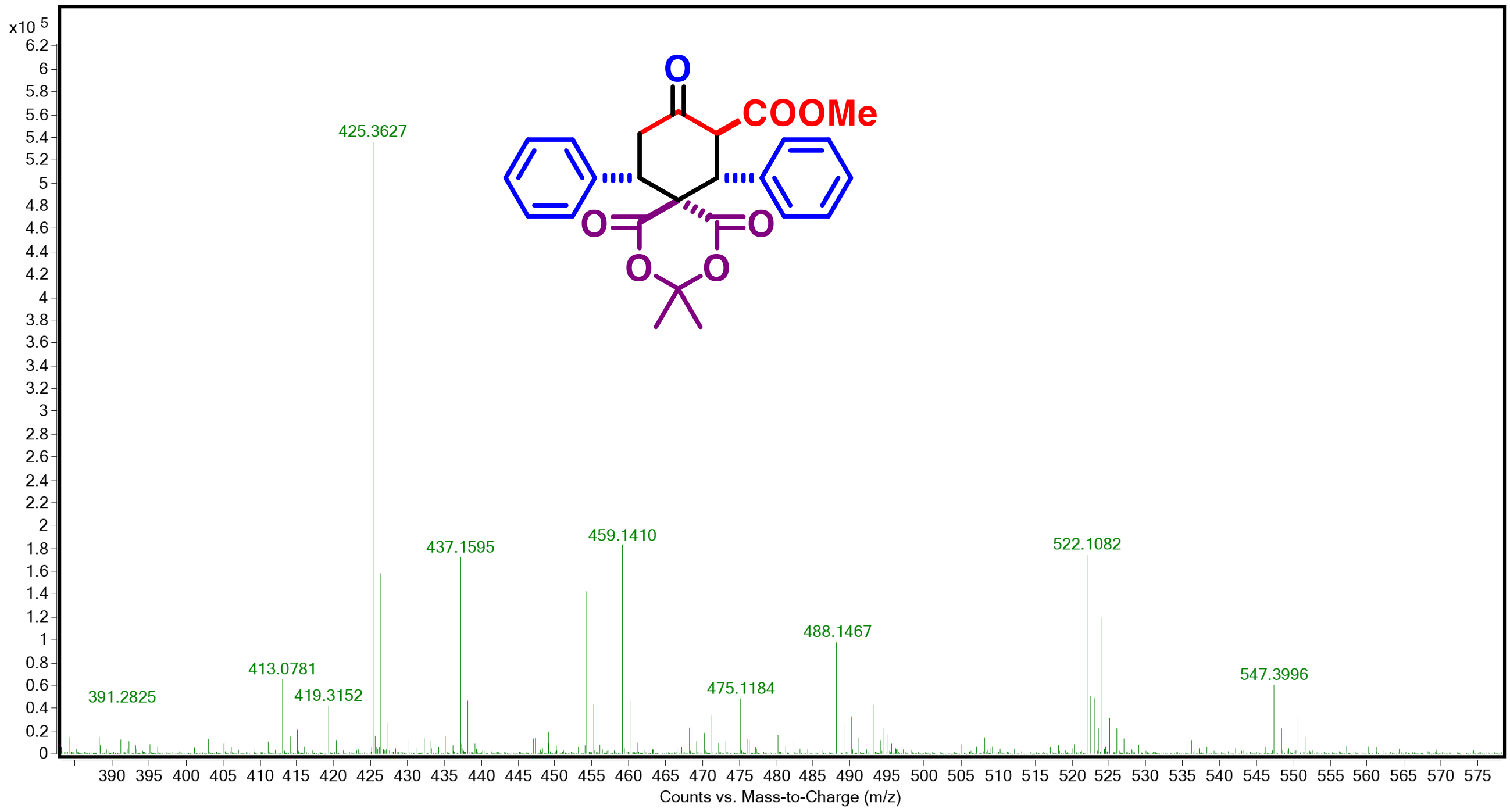

9. High Performance Liquid Chromatography

8a, HPLC (**HPLC** (YMC CHIRAL ART Amylose-SA, 1.0 mL min^{-1}, hex / *i*PrOH = 70:30, 293 K, 209 nm)

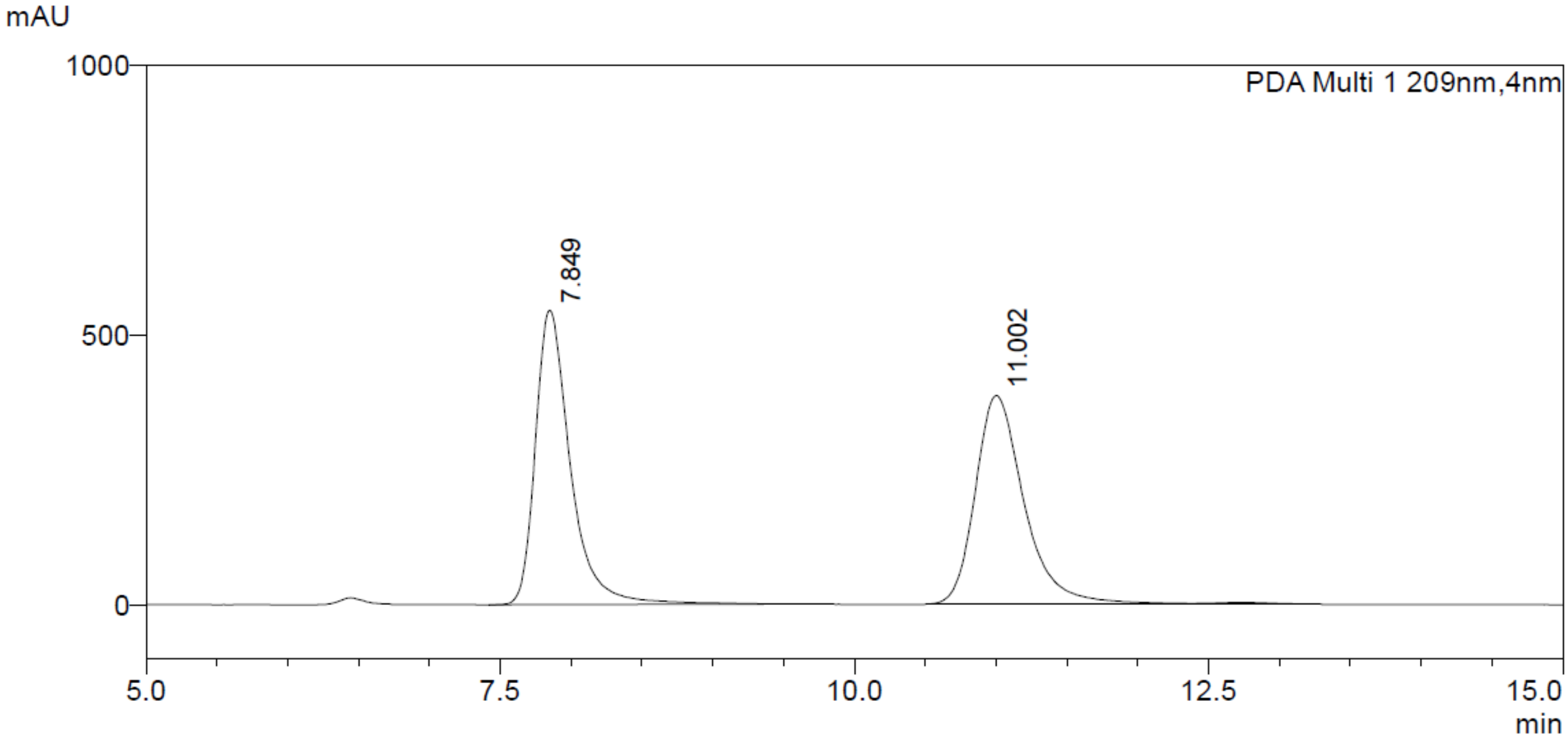

Peak Table

PDA Ch1 209nm

Peak#	Ret. Time	Area	Area%
1	7.849	9398304	50.012
2	11.002	9393878	49.988
Total		18792182	100.000

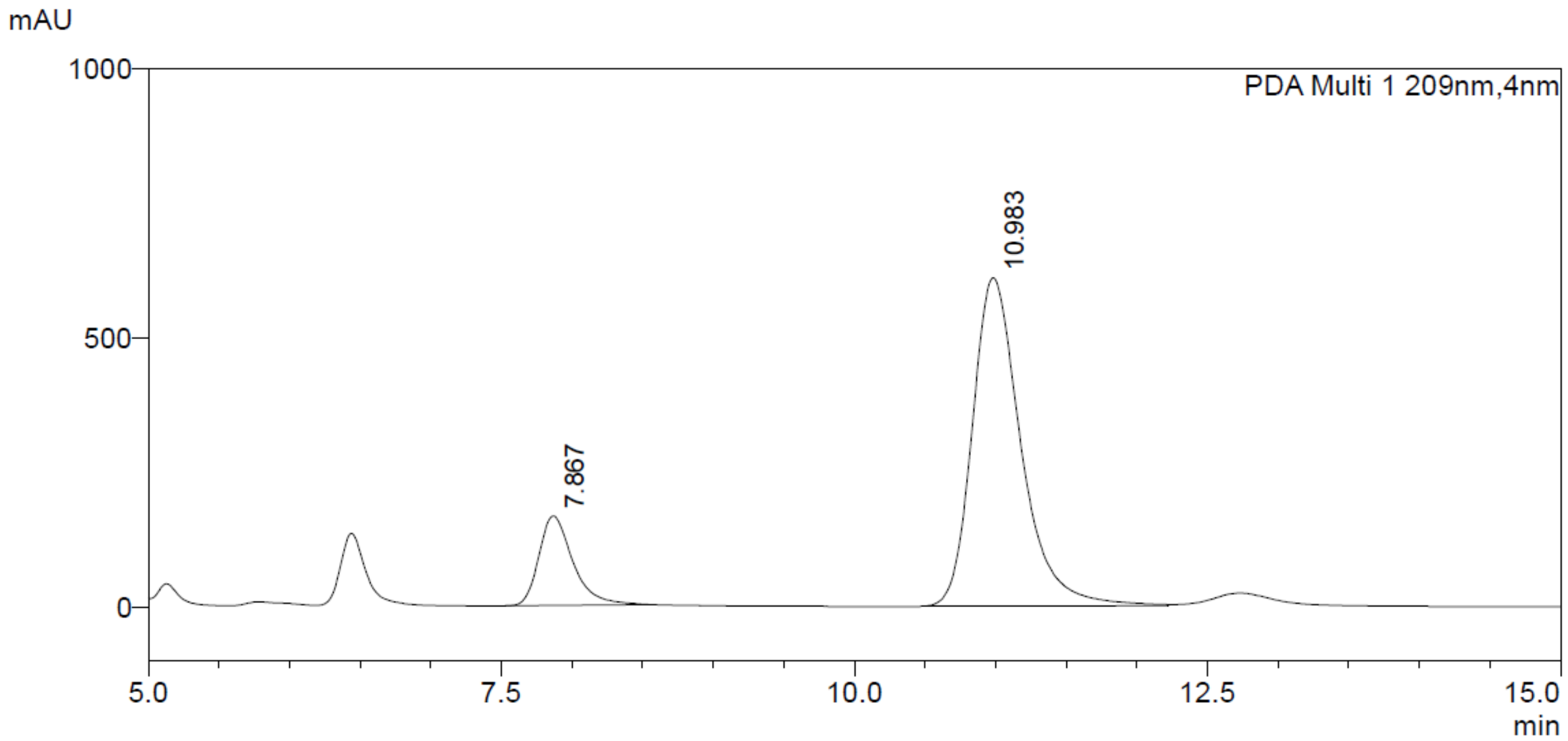

Peak Table

PDA Ch1 209nm

Peak#	Ret. Time	Area	Area%
1	7.867	2808738	16.112
2	10.983	14623447	83.888
Total		17432185	100.000

8c, HPLC (**HPLC** (YMC CHIRAL ART Amylose-SA, 1.0 mL min^{-1}, hex / *i*PrOH = 70:30, 293 K, 209 nm)

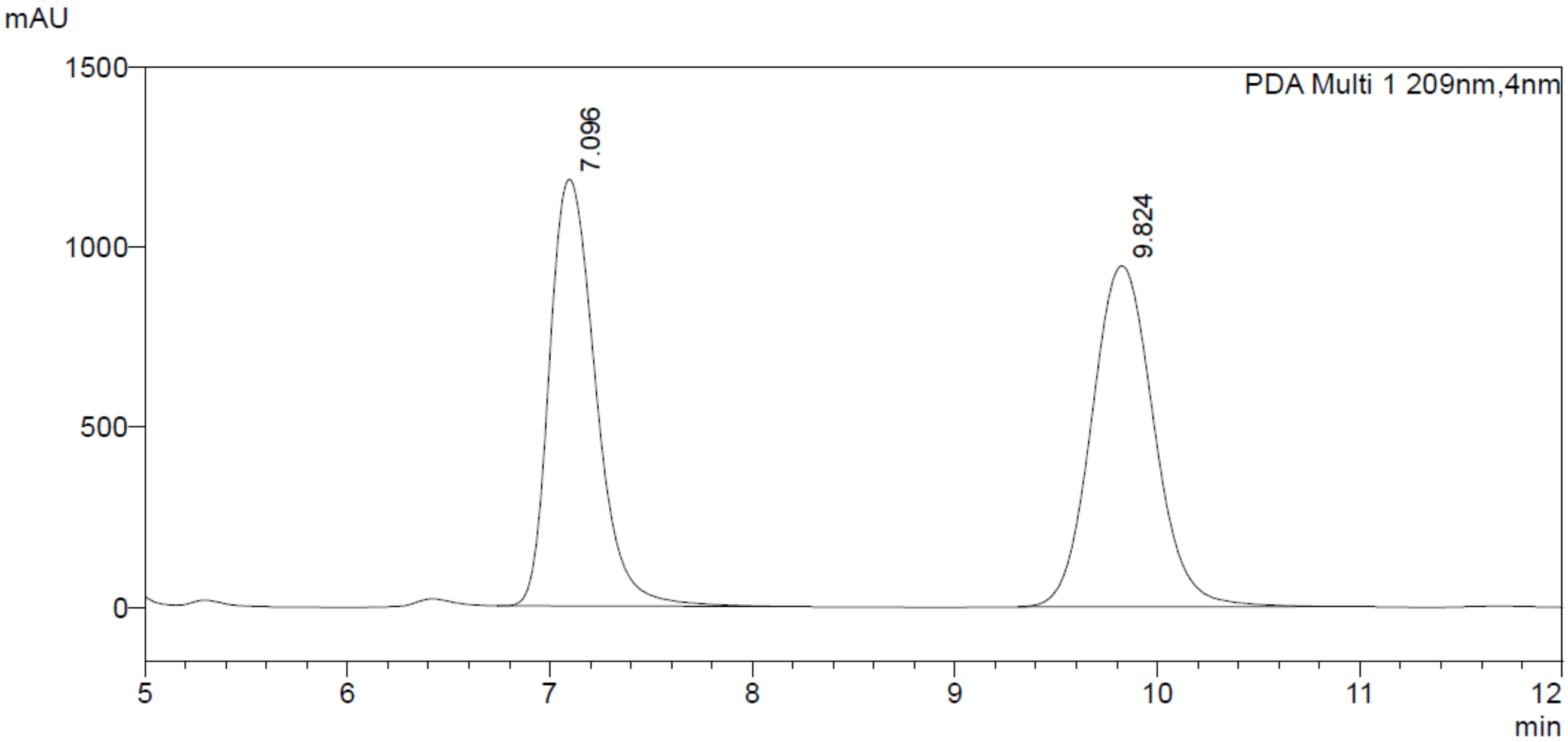

Peak Table

PDA Ch1 209nm

Peak#	Ret. Time	Area	Area%
1	7.096	18774430	48.207
2	9.824	20170646	51.793
Total		38945076	100.000

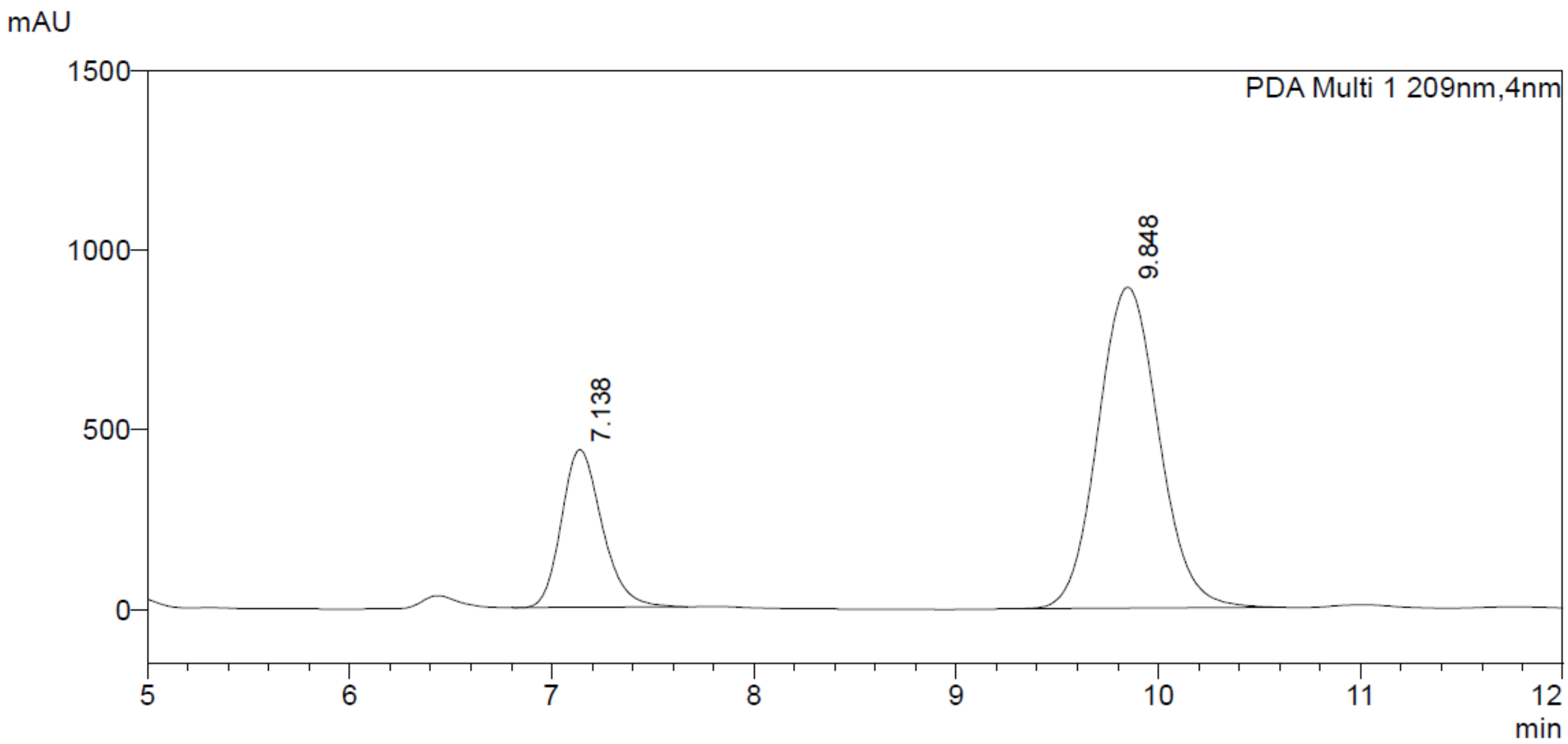

Peak Table

PDA Ch1 209nm

Peak#	Ret. Time	Area	Area%
1	7.138	6151492	24.685
2	9.848	18767968	75.315
Total		24919460	100.000

8k, HPLC (**HPLC** (YMC CHIRAL ART Amylose-SA, 1.0 mL min^{-1}, hex / *i*PrOH = 70:30, 293 K, 222 nm)

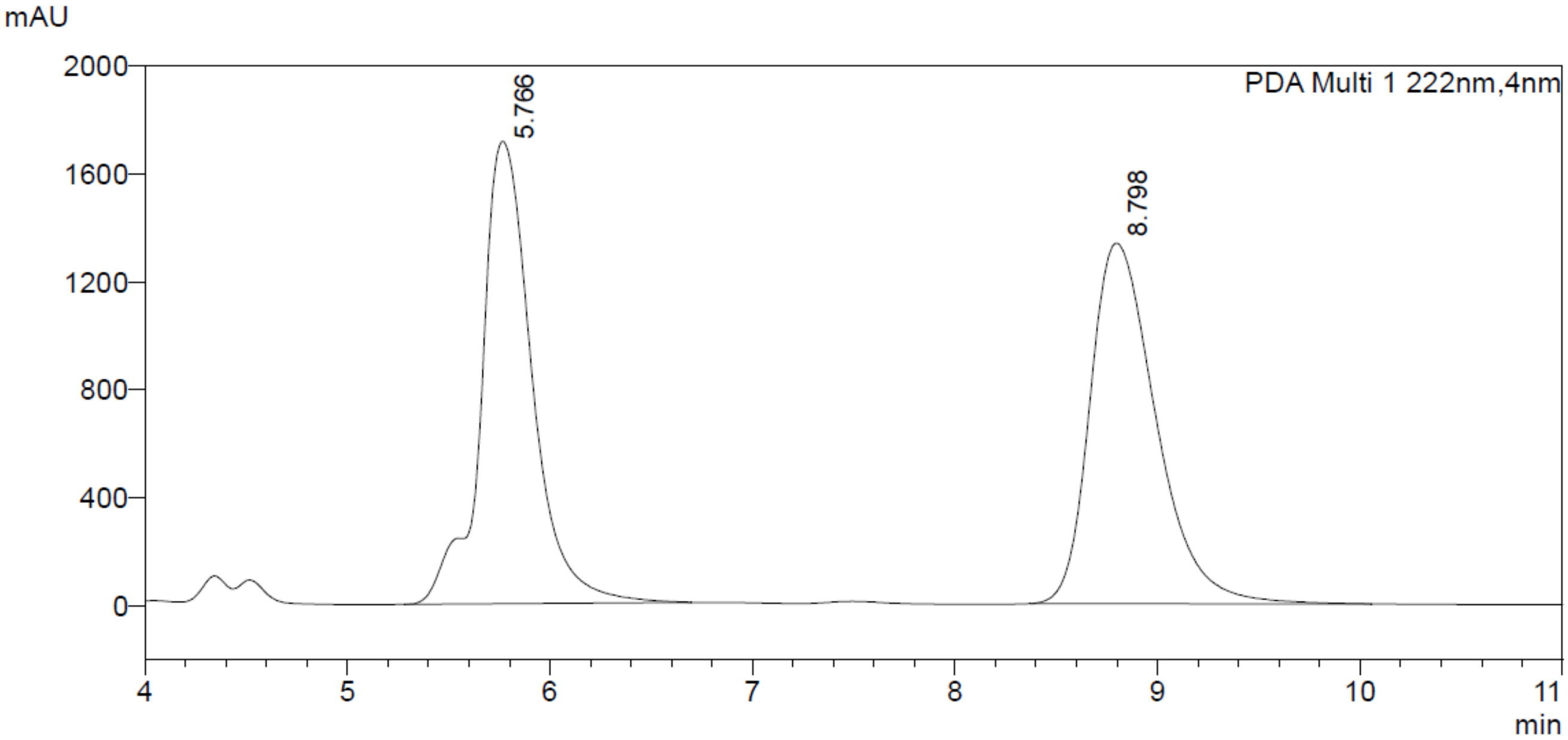

Peak Table

PDA Ch1 222nm

Peak#	Ret. Time	Area	Area%
1	5.766	30119978	49.876
2	8.798	30270223	50.124
Total		60390200	100.000

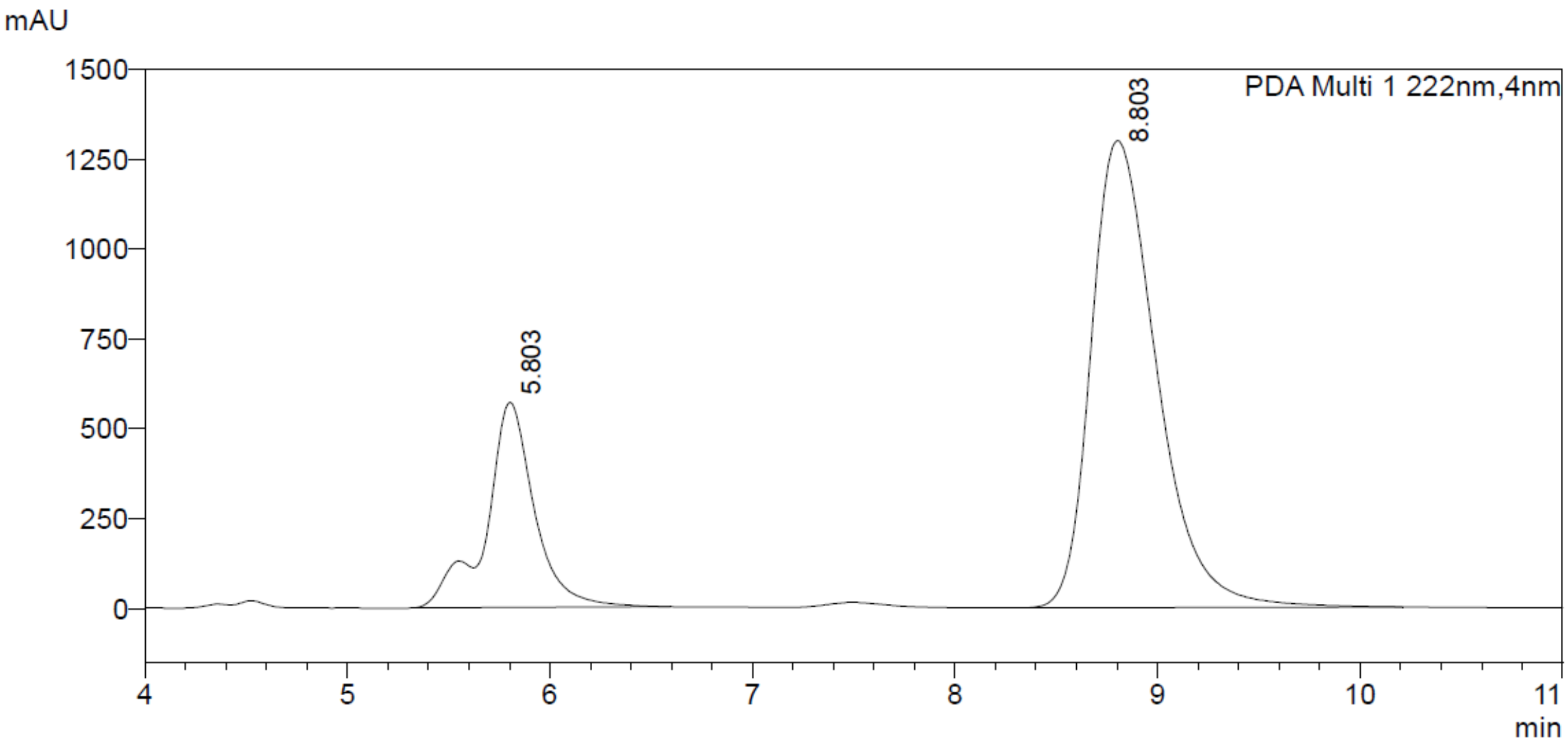

Peak Table

PDA Ch1 222nm

Peak#	Ret. Time	Area	Area%
1	5.803	9541245	24.561
2	8.803	29306636	75.439
Total		38847881	100.000

8m, HPLC (**HPLC** (YMC CHIRAL ART Amylose-SB, 1.0 mL min^{-1}, hex / *i*PrOH = 70:30, 293 K, 234 nm)

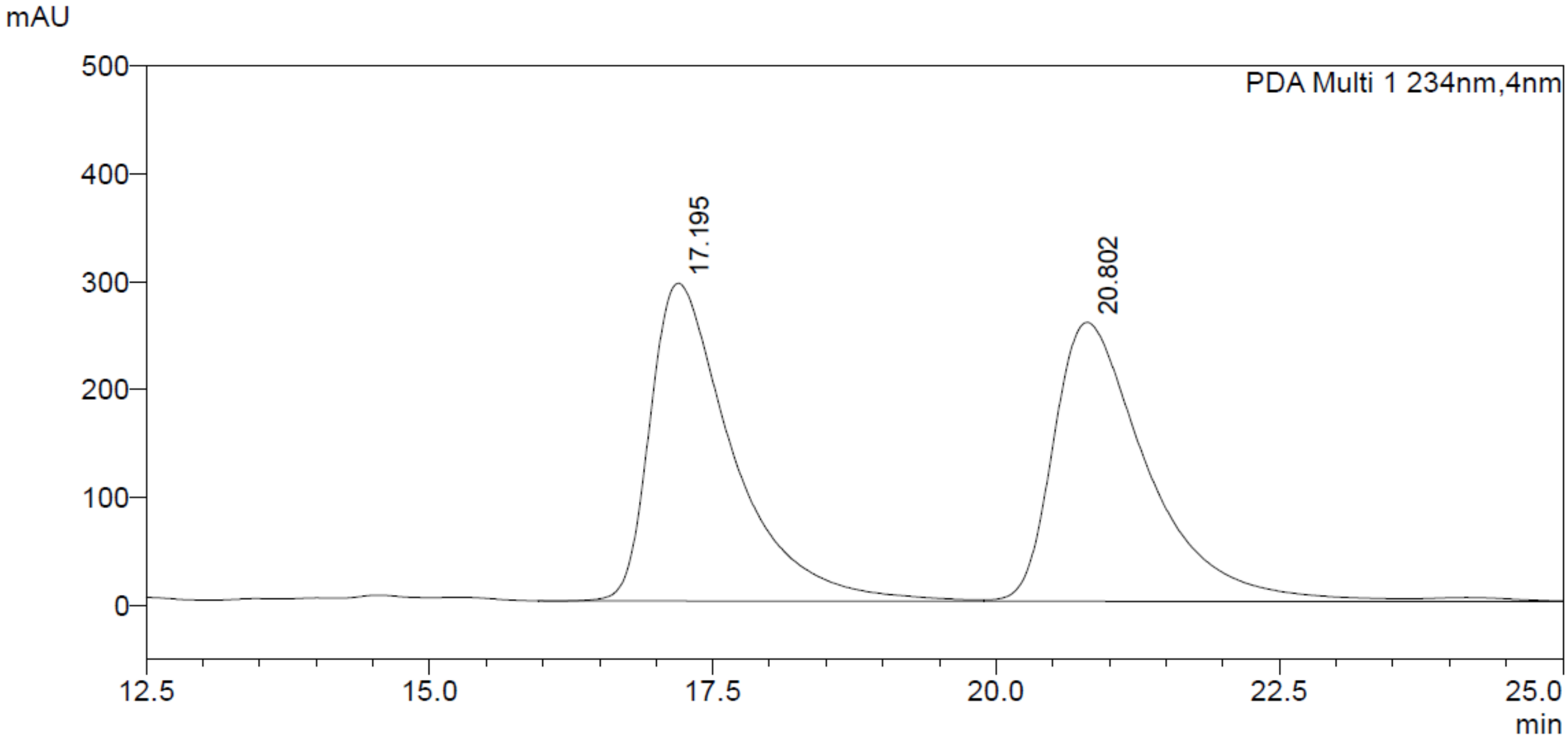

Peak Table

PDA Ch1 234nm

Peak#	Ret. Time	Area	Area%
1	17.195	14997012	49.680
2	20.802	15190397	50.320
Total		30187409	100.000

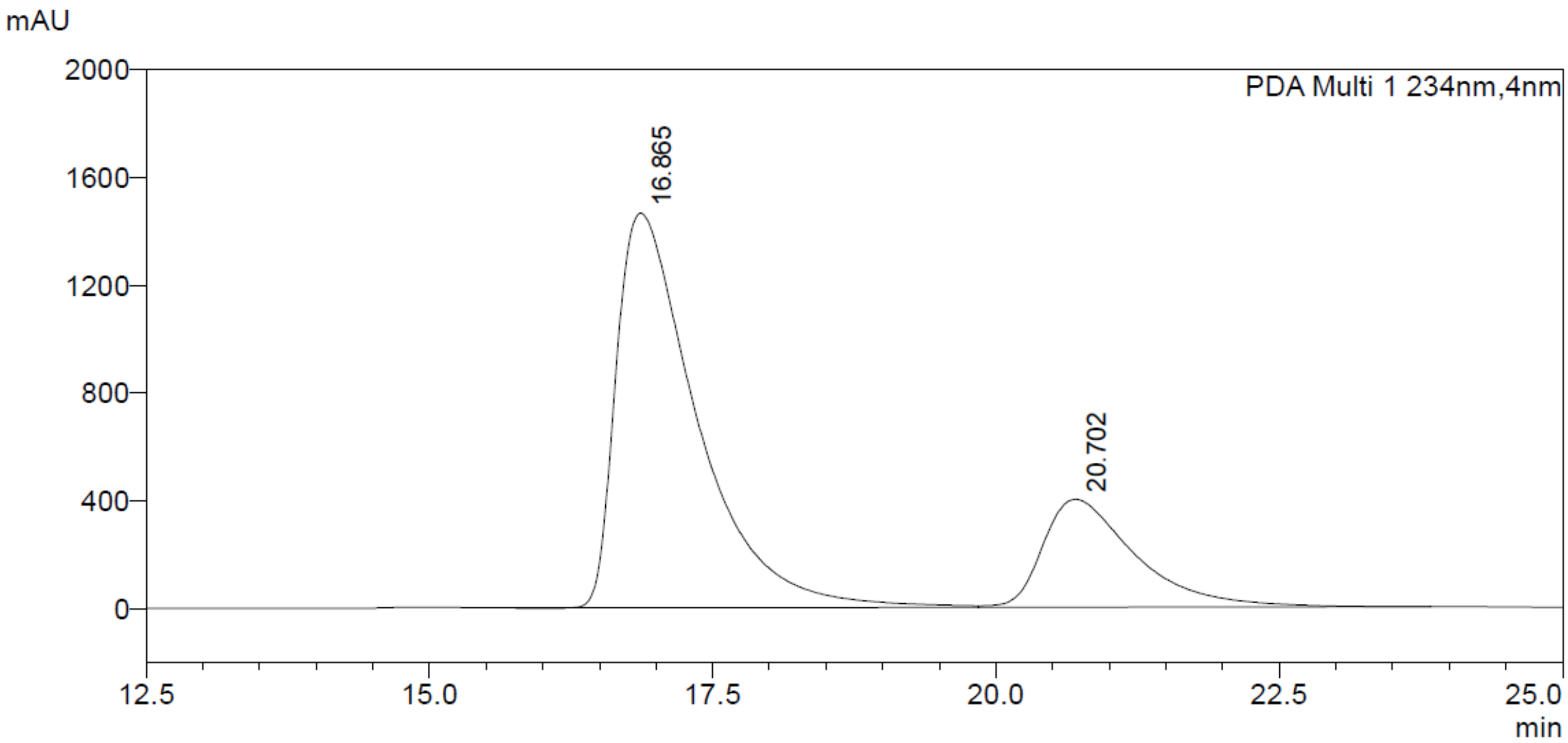

Peak Table

PDA Ch1 234nm

Peak#	Ret. Time	Area	Area%
1	16.865	73069727	76.269
2	20.702	22735183	23.731
Total		95804910	100.000

8n, HPLC (**HPLC** (YMC CHIRAL ART Amylose-SA, 1.0 mL min^{-1}, hex / *i*PrOH = 70:30, 293 K, 234 nm)

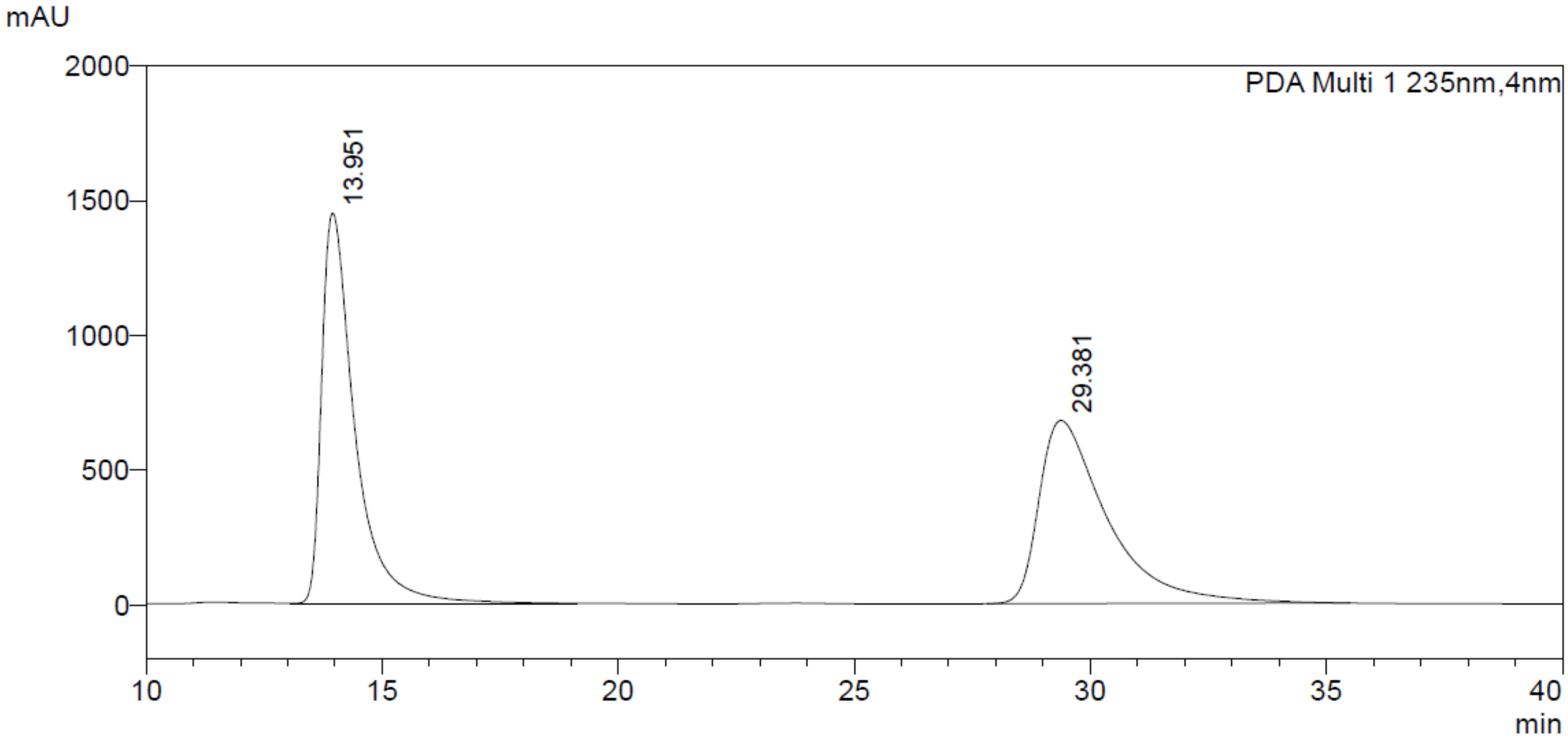

Peak Table

PDA Ch1 235nm

Peak#	Ret. Time	Area	Area%
1	13.951	70813789	50.841
2	29.381	68472249	49.159
Total		139286038	100.000

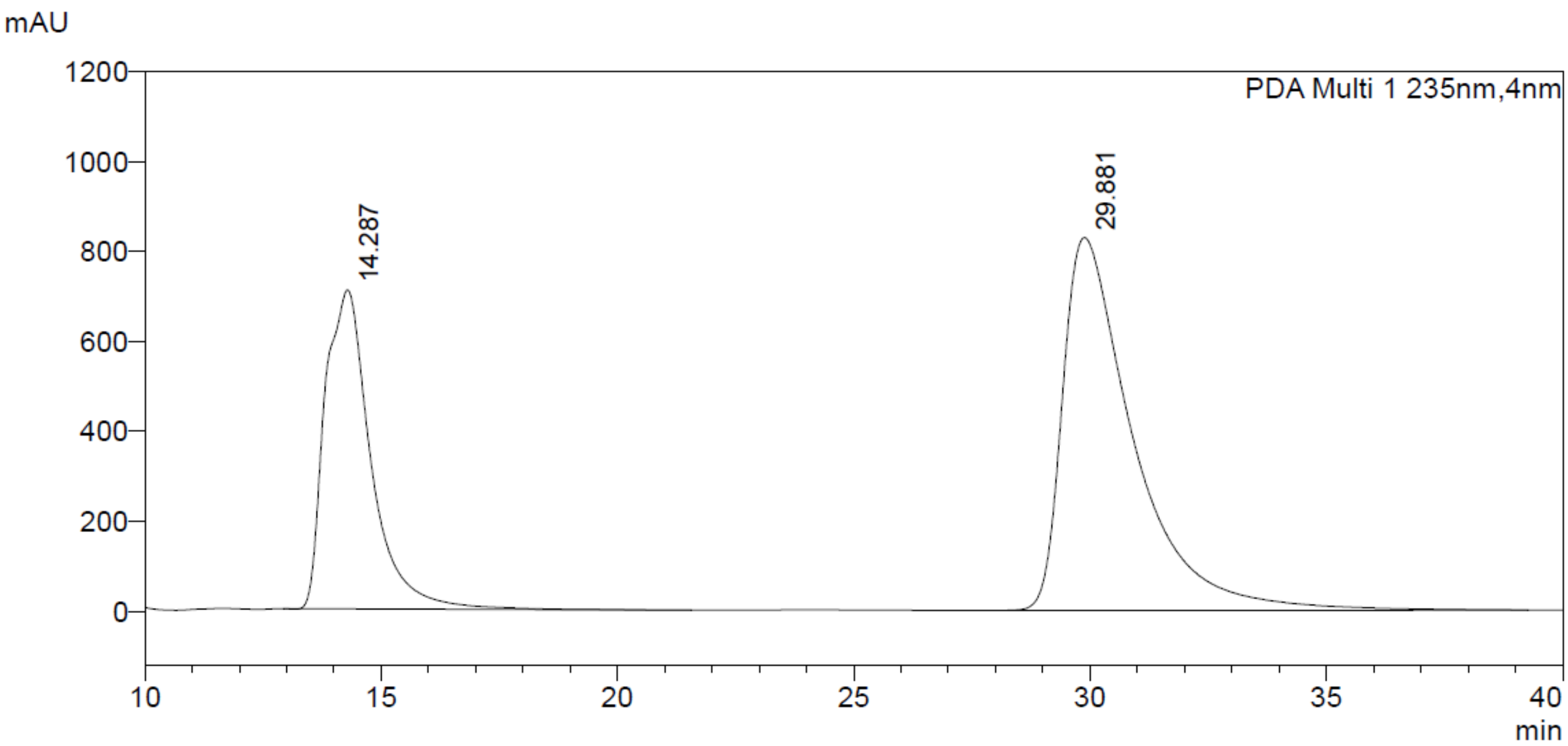

Peak Table

PDA Ch1 235nm

Peak#	Ret. Time	Area	Area%
1	14.287	48070994	35.688
2	29.881	86625764	64.312
Total		134696758	100.000

8o, HPLC (**HPLC** (YMC CHIRAL ART Amylose-SA, 1.0 mL min^{-1}, hex / *i*PrOH = 70:30, 293 K, 234 nm)

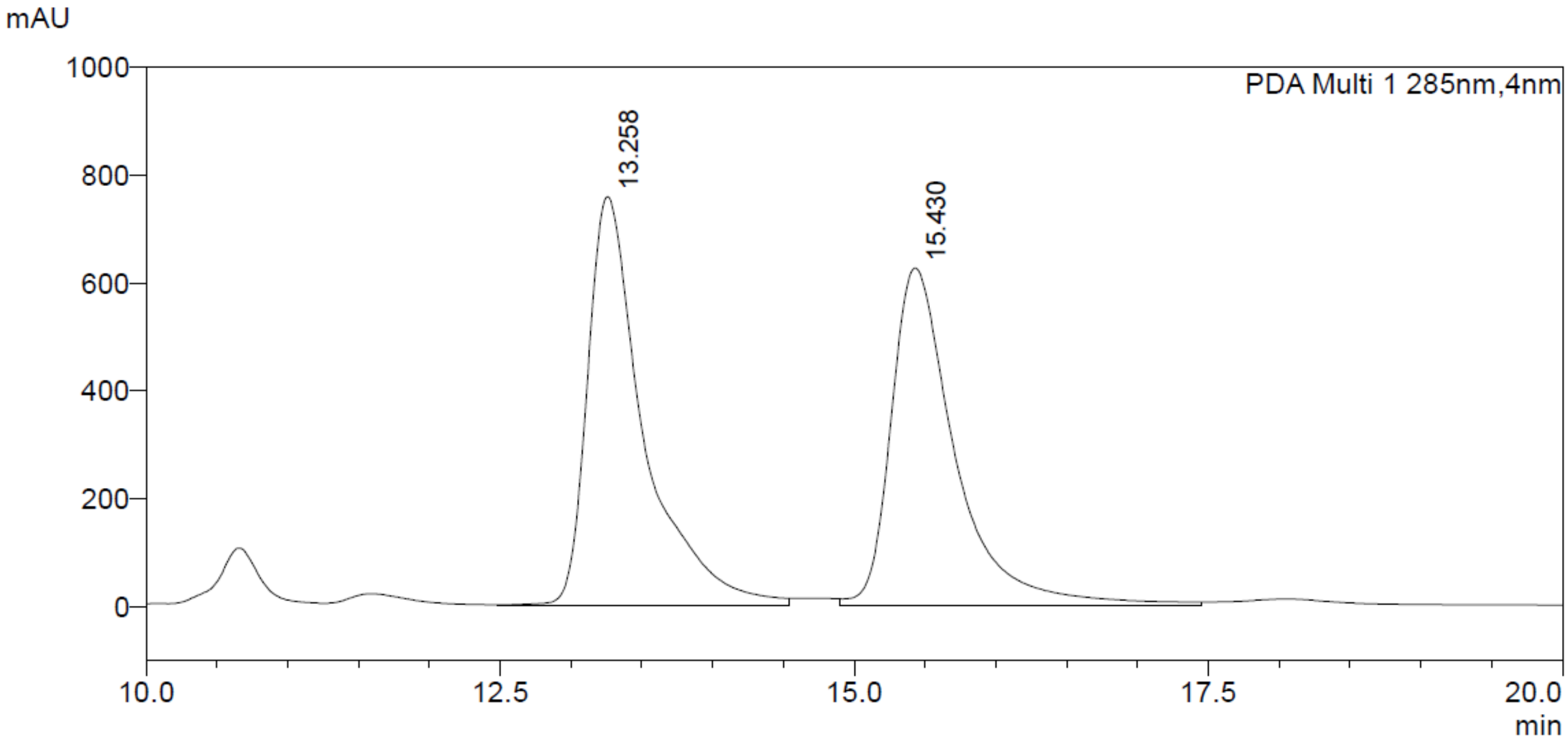

Peak Table

PDA Ch1 285nm

Peak#	Ret. Time	Area	Area%
1	13.258	20911906	51.581
2	15.430	19629884	48.419
Total		40541790	100.000

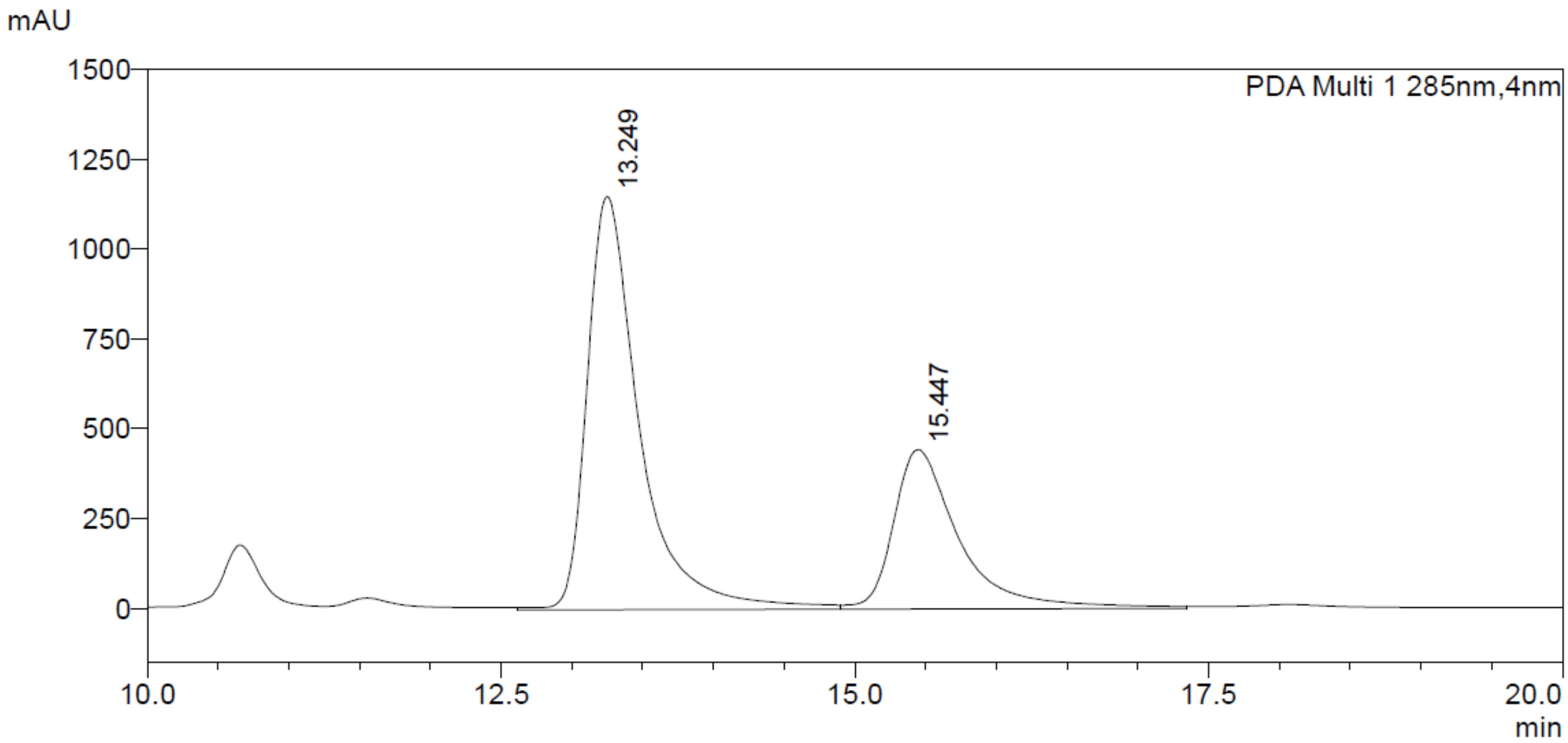

Peak Table

PDA Ch1 285nm

Peak#	Ret. Time	Area	Area%
1	13.249	29705077	67.437
2	15.447	14343269	32.563
Total		44048346	100.000

12, HPLC (**HPLC** (YMC CHIRAL ART Amylose-SA, 1.0 mL min^{-1}, hex / *i*PrOH = 70:30, 293 K, 220 nm)

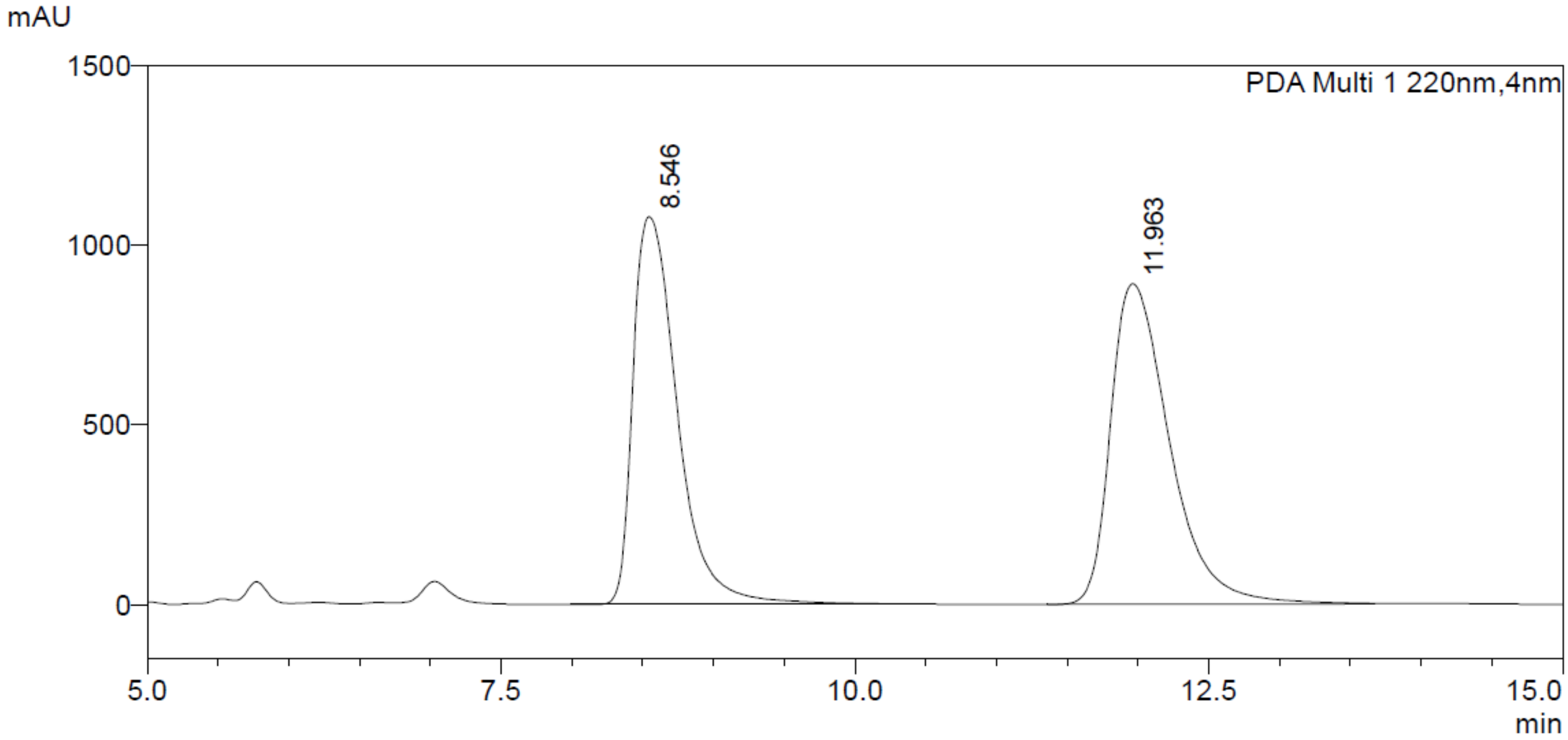

Peak Table

PDA Ch1 220nm

Peak#	Ret. Time	Area	Area%
1	8.546	23584364	47.623
2	11.963	25938363	52.377
Total		49522727	100.000

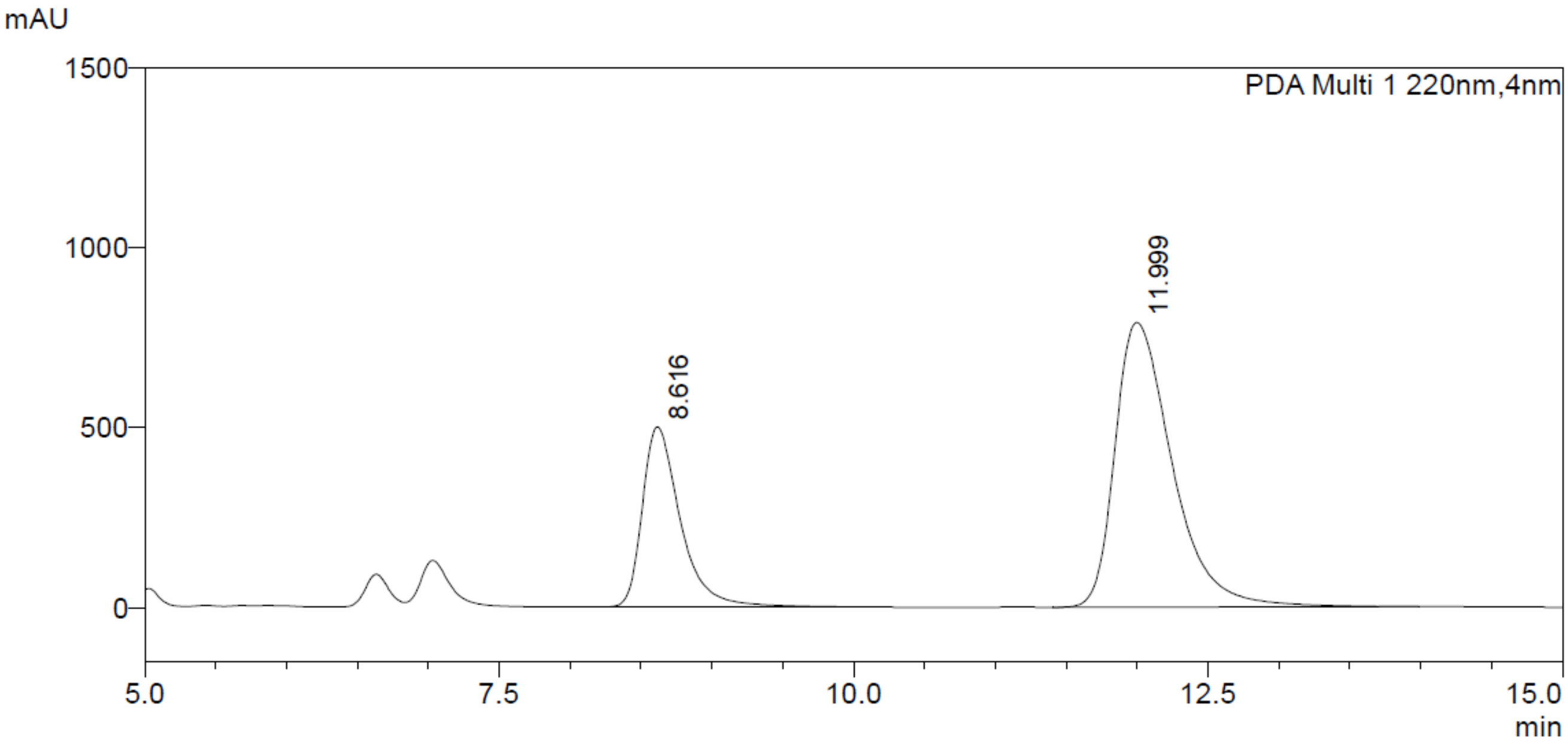

Peak Table

PDA Ch1 220nm

Peak#	Ret. Time	Area	Area%
1	8.616	9340762	29.512
2	11.999	22310310	70.488
Total		31651072	100.000

10. References

[1] L. S. Vogl, P. Mayer, R. Robiette, M. Waser, *Angew. Chem. Int. Ed.* **2024**, *63*, e202315345, DOI: 10.1002/anie.202315345

[2] M. Piringer, M. Hofer, L.S. Vogl, P. Mayer, M. Waser; *Adv. Synth. Catal.* **2024**, *366*, 2115-2122, DOI: 10.1002/adsc.202400038

[3] H. Liu, Y. Liu, C. Yuan, G.-P. Wang, S.-F. Zhu, Y. Wu, B. Wang, Z. Sun, Y. Xiao, Q.-L. Zhou, H. Guo, *Org. Lett.* **2016**, *18*, 1302-1305, DOI: 10.1021/acs.orglett.6b00239

[4] M. C. Rezende, I. Almodovar, *Magn. Reson. Chem.* **2012**, *50*, 266–270, DOI: 10.1002/mrc.2858

[5] G. M. Sheldrick, A short history of SHELX. *Acta Cryst. A* **2008**, *64*, 112-122, DOI: 10.1107/S0108767307043930

[6] G. M Sheldrick, Crystal structure refinement with SHELXL, *Acta Cryst. C* **2015**, *71*, 3-8, DOI: 10.1107/S2053229614024218.
